# Phosphoproteomic analyses reveal novel cross-modulation mechanisms between two signaling pathways in yeast

**DOI:** 10.15252/msb.20145112

**Published:** 2014-12-09

**Authors:** Stefania Vaga, Marti Bernardo-Faura, Thomas Cokelaer, Alessio Maiolica, Christopher A Barnes, Ludovic C Gillet, Björn Hegemann, Frank van Drogen, Hoda Sharifian, Edda Klipp, Matthias Peter, Julio Saez-Rodriguez, Ruedi Aebersold

**Affiliations:** 1 Department of Biology, Institute of Molecular Systems Biology, ETH ZürichZürich, Switzerland; 2 European Molecular Biology Laboratory (EMBL), European Bioinformatics Institute (EBI)Cambridge, UK; 3 Department of Biology, Institute of Biochemistry, ETH ZürichZürich, Switzerland; 4 Department of Biology, Theoretical Biophysics, Humboldt-Universität zu BerlinBerlin, Germany; 5 Faculty of Science, University of ZurichZurich, Switzerland

**Keywords:** cell signaling network, crosstalk, HOG pathway, pheromone pathway, phosphoproteomics

## Abstract

Cells respond to environmental stimuli via specialized signaling pathways. Concurrent stimuli trigger multiple pathways that integrate information, predominantly via protein phosphorylation. Budding yeast responds to NaCl and pheromone via two mitogen-activated protein kinase cascades, the high osmolarity, and the mating pathways, respectively. To investigate signal integration between these pathways, we quantified the time-resolved phosphorylation site dynamics after pathway co-stimulation. Using shotgun mass spectrometry, we quantified 2,536 phosphopeptides across 36 conditions. Our data indicate that NaCl and pheromone affect phosphorylation events within both pathways, which thus affect each other at more levels than anticipated, allowing for information exchange and signal integration. We observed a pheromone-induced down-regulation of Hog1 phosphorylation due to Gpd1, Ste20, Ptp2, Pbs2, and Ptc1. Distinct Ste20 and Pbs2 phosphosites responded differently to the two stimuli, suggesting these proteins as key mediators of the information exchange. A set of logic models was then used to assess the role of measured phosphopeptides in the crosstalk. Our results show that the integration of the response to different stimuli requires complex interconnections between signaling pathways.

## Introduction

Cell survival relies on specific and effective adaptations to environmental changes. Stimuli are detected by specialized receptors and signal transducers that trigger the appropriate responses by activating specific signaling pathways in the cell. These pathways are often comprised of proteins with dynamically changing phosphorylation states that allow for the transmission and integration of information that eventually leads to a functional, coordinated response to the stimulus. These signaling cascades are the core processing circuitries in the cell that allow for detection and transmission of a specific signal. Despite the linear depiction of these signaling cascades in the literature, molecular information actually flows through a highly interconnected cell signaling network that enables cells to make critical decisions based on the overall status of the network. Pathway interaction has been named crosstalk (Schwartz & Baron, 1999), and in the last decade, attempts have been made to measure (Binder & Heinrich, 2004; Komarova *et al*, 2005; Schaber *et al*, 2006; Tisch *et al*, 2014), numerically model (Papin & Palsson, 2004; Behar *et al*, 2007; Fey *et al*, 2012) and mechanistically investigate signaling crosstalk (Dumont *et al*, 2001; Somsen *et al*, 2002; Patterson *et al*, 2010; Waltermann & Klipp, 2010; Baltanas *et al*, 2013).

**Figure 1:**
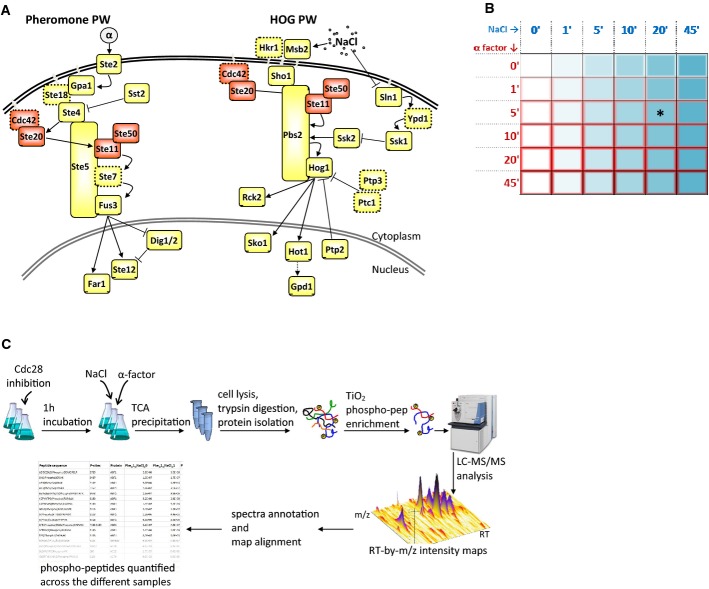
Model pathways and experimental workflow

A prototypic experimental model for studying signaling crosstalk is found in the budding yeast *Saccharomyces cerevisiae* in two of the four mitogen-activated protein kinase (MAPK) pathways, specifically between the high osmolarity glycerol (HOG) and the mating pheromone response pathways (Fig1A) (O'Rourke & Herskowitz, 1998; McClean *et al*, 2007; Westfall *et al*, 2008; Patterson *et al*, 2010; Saito, 2010). The HOG pathway is capable of sensing the increase in extracellular osmolarity by means of two transmembrane osmo-sensors, Sln1 and Sho1. These independently activate two downstream cascades that converge via two different MAPK kinase kinases (Ste11 and Ssk2/Ssk22) on Pbs2, which are both the scaffold protein and the MAPK kinase of the HOG pathway. Once activated, Pbs2 activates the MAPK Hog1. Most of the active Hog1 then relocates to the nucleus and phosphorylates several transcriptional regulators (Posas *et al*, 2000), while a small fraction of the active Hog1 remains in the cytoplasm and phosphorylates other enzymes (Mollapour & Piper, 2007; Westfall *et al*, 2008; Patterson *et al*, 2010). The main result of Hog1 response is an increased cytoplasmic concentration of glycerol, the most common osmolyte in budding yeast (Saito & Posas, 2012), allowing cells to quickly compensate for the increase in extracellular osmotic pressure. Within the pheromone pathway, the mating response in haploid budding yeast cells is triggered upon the detection of pheromones released by cells belonging to the opposite mating type—MATa or MATα. The mating signal is transmitted to the MAPK cascade via a G protein-coupled receptor (Ste2 for MATa, Ste3 for MATα) that activates Ste20, which in turn activates the MAPK cascade comprising Ste11, Ste7 and the MAPK Fus3, which are all bound to Ste5, the pheromone pathway scaffold protein (Elion, 2000; Bardwell, 2004; Dohlman & Slessareva, 2006). A fraction of the active Fus3 then relocates to the nucleus to affect the expression of several genes. Similar to Hog1, part of the active enzyme remains cytoplasmic to phosphorylate cytoplasmic targets (Choi *et al*, 1999; Elion, 2001; Parnell *et al*, 2005).

- The pheromone and HOG pathways (PW) of *Saccharomyces cerevisiae*. In MATa budding yeast cells, the pheromone pathway is activated by α-factor binding to Ste2, which induces the release of the Ste18–Ste4 complex from Gpa1. Ste4 binds Ste20 and Ste5 to initiate signaling. Active Ste20 phosphorylates the MAPK kinase kinase Ste11, which phosphorylates the MAPK kinase Ste7, which phosphorylates the MAPK Fus3. Ste11, Ste7, and Fus3 are all bound to the scaffold protein Ste5. Active Fus3 partially relocates to the nucleus and phosphorylates a number of target proteins (Far1, Ste12, Dig1, Dig2, etc.). The HOG pathway responds to an increase in the external osmolarity by means of two receptors, Sln1 and Sho1, which initiates the response by separately activating the two branches of the pathway. Activated Sho1 recruits the MAPK kinase kinase Ste11 which, together with the MAPK Hog1, is bound to the scaffold protein and MAPK kinase Pbs2. Ste20 phosphorylates Ste11, which phosphorylates Pbs2. Sln1, Ypd1, and Ssk1 constitute a phospho-relay system that inhibits Ssk2 during normal osmotic conditions. During a hyper-osmotic shock, Sln1 is dephosphorylated, thus interrupting the phospho-relay system, and Ssk1 is therefore prevented from inhibiting the other MAPK kinase kinase of the HOG pathway, Ssk2. The latter auto-phosphorylates and then phosphorylates Pbs2. Activated by both branches, Pbs2 phosphorylates Hog1, which partially relocates to the nucleus and phosphorylates several target proteins (Sko1, Hot1, etc.). A set of phosphatases is known to dephosphorylate Hog1 (Ptp2, Ptp3, Ptc1).
- The stimulation matrix for the NaCl and pheromone co-stimulation time course experiment. The stimulation times, indicated in minutes for both NaCl and pheromone, correspond to the total application of either stimulation. As an example, the cell cultures (three biological replicates) relative to the square highlighted by an asterisk were stimulated as follows: NaCl was added to the cultures, which were then placed back in the incubator; 15 min after, pheromone was added to the same cultures, which were then placed again in the incubator; after another 5 min, the cells were harvested. Total duration of NaCl stimulation: 20 min. Total duration of pheromone stimulation: 5 min.
- Workflow for the shotgun mass spectrometry-based label-free quantification of phosphoproteome changes induced by NaCl and pheromone co-stimulation. Cdc28-as budding yeast cells were grown to mid exponential phase. Then, cell cycles were arrested by Cdc28-as inhibition. After 1 h, cells were stimulated by both NaCl and pheromone according to the stimulation matrix (B). Biological processes were stopped by fast ice-cold TCA precipitation. Cells were lysed by bead-beating. Proteins were digested overnight by trypsin. Isolated peptides were enriched for phosphorylated peptides (P-peps) by TiO_2_ resin and then analyzed by an LTQ-Orbitrap XL mass spectrometer. The retention time (RT) versus mass-to-charge ratio (m/z) intensity maps measured were aligned across samples by OpenMS and annotated by Sequest. A list of P-peps quantified across the different stimulation time points was obtained.

The HOG and the pheromone pathways share several components and, as a result, display signaling crosstalk. Specifically, the upstream pheromone pathway and the Sho1-branch of the HOG pathway share Cdc42, Ste20, Ste11, and Ste50 (Fig1A). In particular, Ste20 activates Ste11 in both pathways. It has been hypothesized that response specificity may be achieved by means of scaffold proteins (Patterson *et al*, 2010), kinetic insulation (Behar *et al*, 2007), protein relocation (Yamamoto *et al*, 2010), and/or mutual inhibition (McClean *et al*, 2007). A set of phosphatases is known to de-phosphorylate the two MAPKs in order to inhibit their activity, while another set of proteins, such as Ssk1, Sst2, Dig1, and Dig2, is known to inhibit the activity of different components of the two pathways in other ways. These signaling modules are additionally involved in the complex regulatory mechanisms that generate positive and negative feedback loops, which are used by pathways to modulate the duration and the intensity of their own signals (Hao *et al*, 2008; Schaber *et al*, 2012). While it is well established that the hyper-osmotic stress response inhibits the mating response (Patterson *et al*, 2010), it was more recently reported that also a long pheromone stimulation could reduce Hog1 activation (Yamamoto *et al*, 2010). This phenomenon has been hypothesized to be due to the activity of Ste50—one of the components shared between the HOG and the pheromone pathways. A recent study also reported that pheromone stimulation can activate the HOG pathway in osmo-adapted cells (Baltanas *et al*, 2013). While all of these studies support the existence of mutual modulation between these two pathways, the actual crosstalk mechanisms are still poorly understood.

To systematically investigate the co-modulatory signaling flux between the HOG and the pheromone pathways, we used quantitative shotgun mass spectrometry (MS)-based phosphoproteomics to measure the phosphorylation changes occurring within the cell that are produced by a matrix of time-dependent co-stimulations. For this, we used a double time course experiment in which budding yeast cell cultures were stimulated, both by NaCl and by pheromone, for different times ranging between 0 and 45 min. We then employed label-free quantification MS to measure the phosphorylation changes across the different stimulation times. Our results offer unprecedented and time-resolved details of signal integration within and between these two pathways. In particular, we observed that, for certain components, different phosphorylation sites (P-sites) within the same protein responded to NaCl and pheromone stimulation with idiotypic dynamics, a feature that makes these proteins the key nodes of information exchange and integration. Additionally, our data confirmed that the pheromone pathway is repressed by the HOG pathway, and offer new details on how this is mediated. Interestingly, we detected a significant down-regulation of active Hog1 by pheromone stimulation. By investigating this phenomenon, we propose a few mechanisms to be acting in concert: Shared components (Ste20, Ste11, and Ste50), negative feedback loops (Gpd1), and phosphatases (Ptp2 and Ptc1) are all affecting the time-resolved behavior of active Hog1. To put these multiple observations into a common framework, we developed a set of 23 logic models where each measured phosphopeptide (P-pep) was simulated based on the available prior knowledge of the respective phosphoprotein, and the MS measurements. The model aided the elucidation of the P-peps involved in the crosstalk between the HOG and the pheromone pathways, and assessed the importance for the new mechanisms proposed here. While investigating the dynamics interlacing our two model pathways, we observed a close functional connection between the HOG, the pheromone, and the Target of Rapamycin Complex 2 (TORC2) pathways. Our approach therefore allowed us to predict novel functional interactions between proteins belonging to two model pathways within the cell signaling network, the HOG, and the pheromone pathways, and between these and other components of the network.

## Results

### Measuring the effects of NaCl and α-factor stimulation on the yeast phosphoproteome

To investigate signals integration in the cell signaling network, we studied the response of budding yeast to two stimuli: hyper-osmotic shock by NaCl and α-factor pheromone. The stimuli were applied in a double time course schema consisting of a stimulation matrix, built on two distinct but overlapping stimulation timelines, one relative to NaCl and the other to pheromone stimulation. Both timelines consisted of six time points, ranging between 0 and 45 min, for a total of 36 NaCl_stimulation_period/pheromone_stimulation_period combinations (Fig1B). These stimulation conditions were optimized by a set of preliminary time course experiments where cells were stimulated separately, either by NaCl or by pheromone. Accordingly, early time points after stimulation were favored over later ones, as the transient state of the activation of the two MAPK cascades occurred within 0–10 (salt) or 10–20 (pheromone) min upon stimulation (Supplementary Fig S1).

We employed a shotgun MS-based label-free analytical strategy to quantify P-peps across samples. While budding yeast cells are immediately responsive to osmotic shock, this is not usually the case for pheromone stimulation. To also make cells immediately responsive to pheromone stimulation, a 1 h long Cdc28 analog-sensitive inhibition (Shokat & Velleca, 2002) was employed (Oehlen & Cross, 1994; Colman-Lerner *et al*, 2005; Strickfaden *et al*, 2007). 0.4 M NaCl and 1 μM α-factor were then concurrently or sequentially added to the cell cultures according to the experimental design matrix shown in Fig1B. For each stimulation condition of the matrix, three biological replicates were generated. For practical reasons, the whole perturbation experiment was split into six sub-experiments, one for each row of the matrix, each one corresponding to a NaCl time course at a fixed pheromone stimulation period. All the P-peps were enriched with titanium dioxide, and the samples were measured in batch on an LTQ-Orbitrap using a shotgun approach (Fig1C).

### Dataset validation

To account for both sample and technical variability, and to ensure a robust quantification across samples, the extracted ion intensities of each detected P-pep were first integrated and aligned across all conditions (Sturm *et al*, 2008) and then normalized by the total ion current of the corresponding MS run (Supplementary Fig S2).

**Figure 2:**
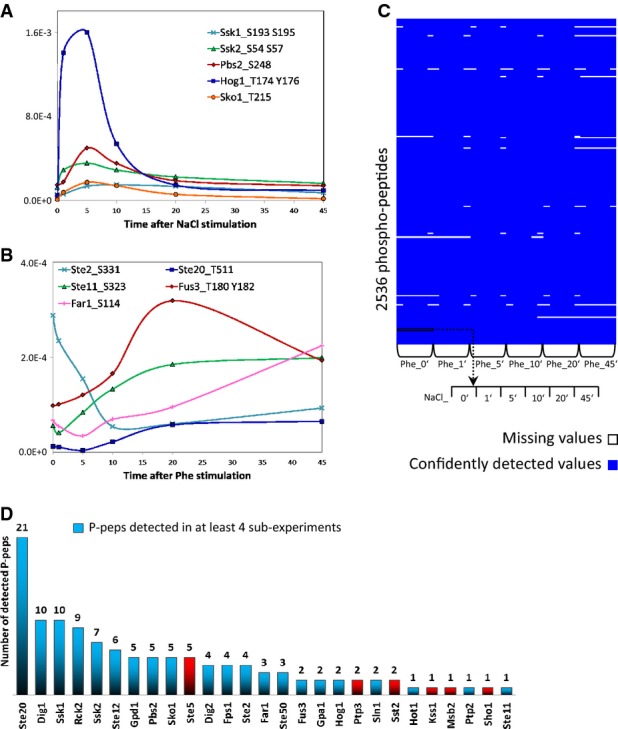
Data overview and validation

To assess the reproducibility of the measured dynamic profiles and their agreement with published data, we performed NaCl-only and pheromone-only time course experiments in duplicate. The known P-peps indicating activation of Hog1 and Fus3, the two MAPKs of the respective pathways, were detectable with a high degree of reproducibility (Supplementary Fig S1), and their activation dynamics were in agreement with published data (Yu *et al*, 2008; Muzzey *et al*, 2009). In particular, Hog1 was quickly phosphorylated at T174 and Y176, reached a maximal phosphorylation level within the first 5′, and was then quickly dephosphorylated. While the curves obtained from the two experiments slightly differ in their shape (there is a secondary mild up-regulation of doubly phosphorylated Hog1), the main spikes are highly reproducible both in shape and in intensity. Fus3, by contrast, exhibited a slower dynamic, as it was gradually phosphorylated at T180 and Y182 during the first 15′, the level of phosphorylation peaked around 20′, and then, it steadily decreased. Furthermore, whereas Fus3 activation curves in the two experiments had similar shapes, their overall intensities differed. This difference may be due to a dissimilar starting amount either of doubly phosphorylated Fus3 or of the activating kinases upstream to Fus3 (such as Ste7). The phosphorylation dynamics of further key components of the two MAPK cascades followed patterns similar to those of their corresponding MAPK (Fig2A and B). These results confirm that the conditions used correctly reproduced the expected dynamics of the measured P-peps.

- A, B Dynamic curves of a representative set of phosphopeptides (P-peps) of the HOG pathway after NaCl stimulation only (A) and of the pheromone pathway after α-factor stimulation only (B). The data points of the curves in these two panels correspond, respectively, to the first row and the first column of the matrix in Fig1B.
- C 2,536 P-peps, belonging to 1,015 unique proteins, were quantified in at least one of the three biological replicates, of at least four of the six NaCl and pheromone time points with a FDR < 1%. In this panel, we highlight the missing values of our dataset in white.
- D Number of P-peps detected for each component of the HOG and pheromone pathway. Proteins with blue-coded bars have at least one P-pep with enough confidently detected values (as explained for C), while proteins with red-coded bars do not.

### Computation of the observed phosphopeptide dynamic profiles and qualitative exploration of the HOG and pheromone pathways dataset

Within our normalized dataset, we focused our analyses on those P-peps that were confidently detected with a false discovery rate < 1% in at least one of the three biological replicates of at least four of the six NaCl and pheromone time points (Elias & Gygi, 2007). These filters produced a final dataset of 2,536 P-peps, belonging to 1,015 unique proteins (Fig2C), thus covering about 17% of the budding yeast proteome. Given the complexity of the signaling network, here we exclusively studied P-peps derived from the proteins associated with the HOG and/or pheromone pathways, with the purpose of investigating how these two model interconnected signaling cascades transmit and integrate information within and between themselves.

A large majority of the known proteins associated with the HOG (15 out of 20) and pheromone (12 out of 15) pathways were measured. At least one phosphopeptide of 82% of these proteins could be detected in at least one of the six sub-experiments. (Dotted-lined proteins in Fig1A could not be detected). On average, we could quantify 4 P-peps for each protein. The protein with the highest number of P-peps was Ste20 with 21 quantified sites (Fig2D). The proteins that we could not detect are from challenging segments of the proteome, such as membrane-bound, low molecular weight, or low copy number. The full time course dataset of all the confidently detected P-peps relative to the HOG and pheromone pathways is available in Supplementary Table S1 where, for each P-pep and for each NaCl–pheromone co-stimulation periods, we indicate the number of detected biological replicates, their average intensity, and their standard deviation.

Interestingly, 58% of the naked sequences (i.e. backbone sequences without phosphorylations) that were identified contain either the SP or the TP motifs (Supplementary Table S1), which have been identified as the recognition motifs of all yeast MAP kinases (Mok *et al*, 2010). This is consistent with many of the detected P-sites being direct targets of Hog1 and/or Fus3.

**Figure 3:**
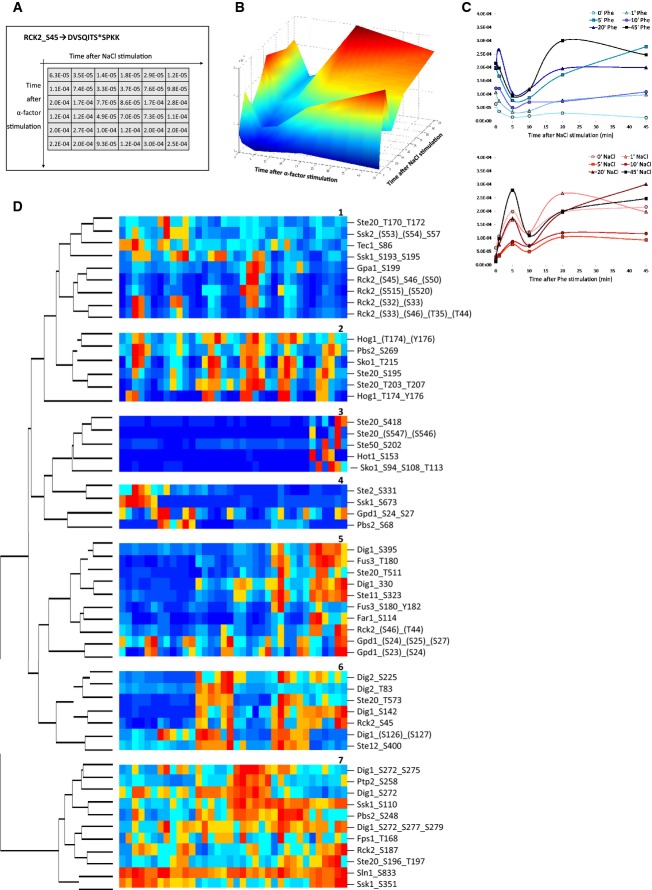
Data representation and clustering

To investigate changes in phosphorylation, we represented the P-peps measured dynamics (Fig3A) as a 3D graph (Fig3B) and as a combination of 2D charts (Fig3C) representing sections through the 3D graph. In the 2D representation, each curve follows the dynamic profile of the corresponding P-pep along one of the two stimuli's axis (Stimulus_1). Each curve differs from the other superimposed curves in the same graph by the period of the application of the second stimulus (Stimulus_2).

- A–C For each quantified phosphopeptide (P-pep), a matrix of intensities was computed (A), where missing values were estimated by cubic spline data interpolation. This can be represented as a 3-dimensional surface (B), and as a set of 2-dimensional curves (C) where each curve, while following the dynamic of the corresponding P-pep along one of the two stimuli's timelines on the *x*-axis (Stimulus_1), differs from the other superimposed curves by the time of the application of the other stimulus (Stimulus_2).
- D Hierarchical clustering with Minkowski distance performed on the intensity data of the quantified P-peps of the HOG and pheromone pathways generated seven main clusters. Some of the most interesting P-peps of each cluster are reported to the right.

We performed hierarchical clustering of the intensities for all the 36 NaCl/pheromone stimulation combinations to obtain a global view of the regulation of the P-peps that are known to associate with the HOG and the pheromone pathways, respectively. In Fig3D, we highlight the seven main clusters that arose from this analysis. Clusters 1, 2, and 4 mostly include P-peps of HOG pathway components, while clusters 5 and 6 include P-peps that mostly associate with the pheromone pathway. Clusters 3 and 7 contain P-peps that either associate with the HOG pathway or are shared with the pheromone pathway.

This clustering approach showed that almost all components of both pathways are affected by both stimuli. This is a surprising finding, given that pathway crosstalk has so far been linked to very few components. Cluster 2 contains the HOG pathway's P-peps that show cross-stimulation dynamics similar to Hog1_T174_Y176 (ppHog1). All these P-peps appear to be affected by pheromone-only 1′ after pheromone stimulation: They were all down-regulated when cells were harvested 1′ after pheromone stimulation. In contrast, P-peps of clusters 1 and 4 were down-regulated by long pheromone stimulation even though they are associated with HOG pathway proteins. In contrast, the P-peps belonging to cluster 3 were up-regulated by long pheromone stimulation. Cluster 6 P-peps were up-regulated after 5 min of pheromone stimulation, while those belonging to cluster 5 were up-regulated 10′–20′ after pheromone stimulation. Interestingly, cluster 7 consisted of several P-peps from components of both pathways, most of which have inhibitory effects over other pathway components. Ssk1_S110 and Pbs2_S248, which fall into cluster 7, were found to have similar dynamics and were also up-regulated by pheromone despite their association with the HOG pathway. We also observed that Ste20's P-peps appeared in all clusters except cluster 4, indicating that P-peps of Ste20 are differentially regulated by the two stimuli, therefore presumably playing multiple roles in the signal integration within and between the two pathways.

Overall, this analysis allowed us to identify different classes of behavior within the pathway components. Interestingly, the majority of the detected P-peps responded to both NaCl and pheromone. This suggests that the HOG and the pheromone pathways are tightly interconnected, exchanging information at multiple levels.

### A classification of NaCl- or pheromone-induced effects on dynamic P-pep patterns

**Figure 4:**
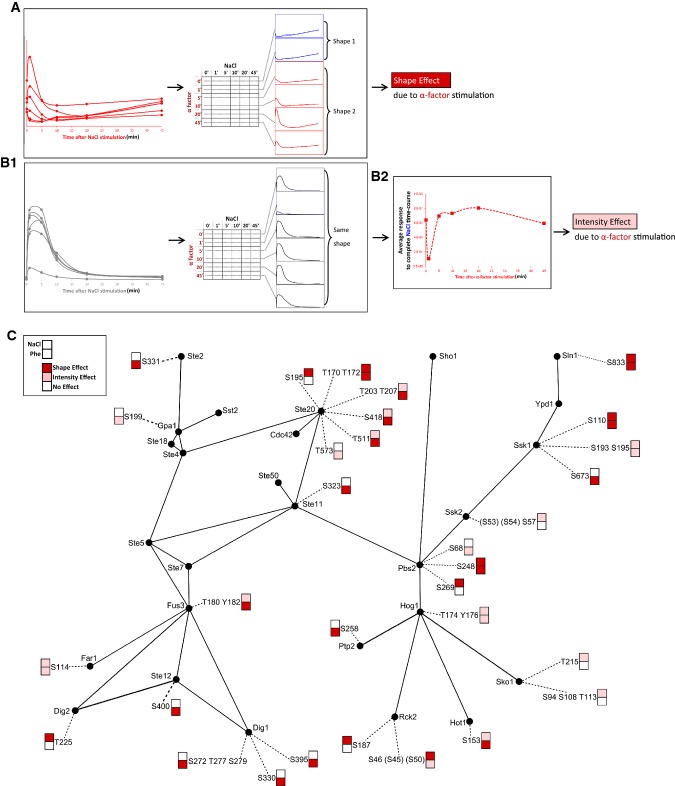
Classification of phosphopeptides (P-peps) according to how NaCl and pheromone affect the shape of their dynamic curves

To better understand how the co-stimulation affected the dynamic P-pep patterns, we manually investigated their 2D representations (Fig3C) along the time-axes for both stimuli. This analysis showed that, in the case of some P-peps, the length of the application of Stimulus_2 (either NaCl or pheromone) significantly changed the shape of the curves plotted against the time following the application of Stimulus_1 (pheromone or NaCl, respectively). In the following, we call this the “Shape Effect” of Stimulus_2 (Fig4A). Most of the P-pep changes following this pattern occurred in the first 5′ following Stimulus_1 application, and they mostly appeared as changes in curve concavity, as an increase/decrease in the number of maximums and minimums of the curves (e.g. a biphasic curve becomes triphasic), or as a change in curve shape with earlier or later onset. All these patterns suggest that Stimulus_2 significantly affected the dynamics of these P-peps by altering their behavior along Stimulus_1 time-axis.

- The “Shape Effect” induced by Stimulus_2 significantly changes the shape of the curves along the timeline of Stimulus_1.
- The “Intensity Effect” induced by Stimulus_2 does not affect the shape (B1) but the intensity (B2) of the curves along the timeline of Stimulus_1. In both (A) and (B), Stimulus_2 is pheromone and Stimulus_1 is NaCl.
- The most significant results of the Shape and Intensity Effects classification are reported here, for the HOG and pheromone pathway P-peps, using a protein topology derived from previous knowledge (Fig1A). For each P-pep, we display two color-coded squares: The top one shows the effect of NaCl on the P-pep pheromone dynamics, and the bottom one shows the effect of pheromone on the P-pep NaCl dynamics.

The dynamics of a second group of P-peps, once plotted against Stimulus_1 time-axis (for instance), while displaying unvarying curve shapes, exhibited overall significant intensity variability modulated by Stimulus_2 (Fig4B). Here, we call this the “Intensity Effect” of Stimulus_2, as it does not alter the behavior of the P-peps but it significantly increases or decreases its overall phosphorylation. Each P-pep was thus classified by a Shape Effect, an Intensity Effect, or No Effect after co-stimulation with NaCl, pheromone, or both (Fig4C).

By analyzing the distribution of the two stimuli's Effects across conditions, we observed that many P-peps, which are not presently known as shared components of the two pathways, were affected by both stimuli. We also noted some P-peps that were only affected by the stimulus not previously linked to its own specific pathway. For example, Ssk1_S673 and Pbs2_S68, both associated with the HOG pathway, were significantly affected only by pheromone. Interestingly, the majority of the Shape Effects within the pathway components can be attributed to pheromone (because for pheromone, 55% of the P-peps undergo a Shape Effect, 22.5% undergo an Intensity Effect, and 22.5% are not affected), while the majority of the Intensity Effects are due to NaCl (because for NaCl, 35.5% of the P-peps undergo an Intensity Effect, 29% undergo a Shape Effect, and 35.5% are not affected). This finding implies that, while pheromone stimulation qualitatively changes the way the pathway components respond, NaCl affects pathway components quantitatively.

As most of the Shape Effects and, to a certain extent, also the Intensity Effects, occurred in the earliest time points (within 5′), we wondered whether these might be induced artifactually by the handling of the cultures which, in the case of very early time points, was temporally very close to culture harvesting. We therefore performed two mock time course experiments. The first was in relation to row 2 of the matrix: An equivalent volume PBS, instead of pheromone, was administered to the cultures 1 min before harvesting (mock_1′_Phe time course). The second was in relation to column 2 of the matrix (mock_1′_NaCl time course) where, instead of 4M SD medium, an equivalent volume of NaCl-free SD medium was administered to the cells 1 min before harvesting. We then compared the normalized intensities measured in the mock_1′_Phe time course experiment to those of the first row of the matrix, and the intensities of mock_1′_NaCl time course to the first column of the matrix. We considered the difference between the matrix time course data and its corresponding mock experiment to be negligible if one value was < 1.5 times higher/smaller than the other one. Within the HOG and pheromone pathways components, 83% of the detected P-peps did not exhibit significant differences between the 0′_Pheromone_matrix time course and the mock_1′_Phe time course, and 89% did not exhibit a significant difference between the 0′_NaCl_matrix time course and the mock_1′_NaCl time course. We can therefore conclude that the detected dynamics are generated by the stimulation rather than by culture handling. All the results of the mock experiments are reported in Supplementary Table S2.

### Stimuli crosstalk is causing Hog1 and Fus3 P-peps down-regulation

**Figure 5:**
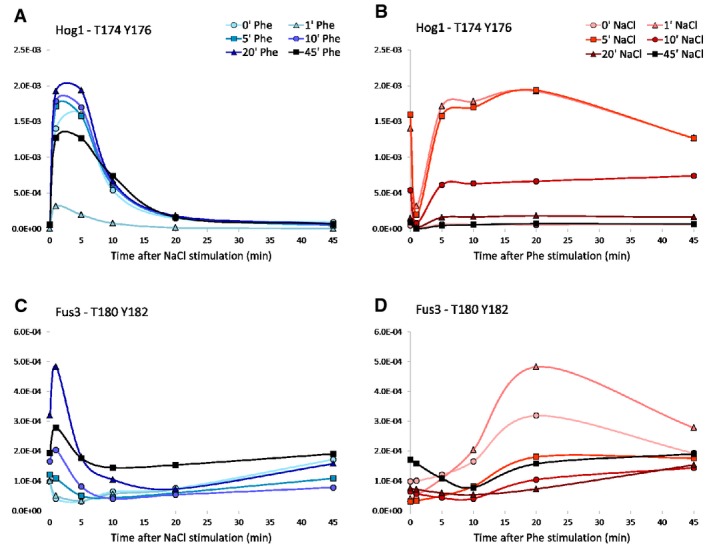
Hog1 and Fus3 activation sites

According to the classification introduced above, we analyzed the behavior of the pathway MAPKs after co-stimulation with the respective opposite pathway: ppHog1 from pheromone and Fus3_T180_Y182 (ppFus3) from NaCl stimulation (Fig4C). Surprisingly, ppHog1 underwent an Intensity Effect displayed by a strong and short-lived down-regulation 1′ after pheromone stimulation (Fig5A), before recovering its full intensity within the next 4 min (Fig5B). This brief inhibitory effect of pheromone on the HOG pathway MAPK is an unexpected behavior, since the osmotic shock response is of higher priority for the cell compared to the mating response. ppHog1 maximum intensity was not reduced when cells were harvested 1 min after mock pheromone stimulation (Supplementary Fig S3). The intensity reached by ppHog1 in the mock_1′_Phe time course was, indeed, comparable to those measured for all the time courses of our matrix experiments, except for the one relative to 1′ pheromone stimulation. When comparing the dynamics of the mock_1′_Phe time course to that of the first row of our matrix, where no pheromone stimulation was applied, we observed similar curves both reaching comparable intensities (Supplementary Fig S3). These results suggest that the down-regulation of ppHog1 observed 1′ after pheromone stimulation is due to the stimulation itself rather than to a stress response induced by culture handling.

- A–D The 2-dimensional dynamic curves (see Fig3C) of the doubly phosphorylated forms of the activation peptides of the Hog1 and Fus3 MAP kinases: Hog1_T174_Y176 (A, B) and Fus3_T180_Y182 (C, D). Missing values were estimated by cubic spline data interpolation (see Supplementary Table S1 for all details).

The dynamic profile of ppFus3 did not change along the pheromone timeline (Fig5C) but, as expected (O'Rourke & Herskowitz, 1998; McClean *et al*, 2007), the maximum intensity reached by the P-pep was down-regulated by NaCl stimulation (Fig5D). This phenomenon does not prevent Fus3 activation, but slightly slows the initial response. Interestingly, however, when the system was pre-stimulated by pheromone for at least 10′ prior to NaCl stimulation, NaCl transiently further activated ppFus3 (Fig5C). These data show that, although both MAPKs influence each other as expected, the response dynamics during short time spans can vary from the final pathway output.

We further analyzed whether the observed mutual influence of the two MAPKs is also carried to their respective downstream targets. Among Fus3 targets, only Dig2_T225 and Far1_S114 were influenced by NaCl, while both Ste12_S400 and the Dig1 P-peps were affected only by pheromone, and all with a Shape Effect (Fig4C). The observation that Dig2_T225 was not modulated by pheromone suggests that this particular P-site is not the one targeted by Fus3. Lastly, Hot1_S153 is the only Hog1 target influenced by pheromone with a Shape Effect, while NaCl induces an Intensity Effect over the same P-site.

These data show that the HOG and the pheromone pathway MAPKs are both affected by the crosstalk induced by the co-stimulation by NaCl and pheromone. In particular, with specific stimulation conditions, crosstalk is down-regulating the phosphorylation of the activating P-sites of the two MAPKs.

### Particular P-sites of upstream components show NaCl or pheromone idiotypic patterns

Among the components that are upstream of the MAPKs in the respective pathways, the only ones whose P-peps were influenced by both signals are Pbs2, Ssk1, and Sln1 from the HOG pathway, and Ste20, a kinase shared between the two pathways (Fig4C). Interestingly, in many cases, several P-peps of the same protein responded exclusively to one or the other stimulus, while others were affected by both stimuli.

Ste20, which had the highest number of detected P-peps in our study, has been reported to be phosphorylated at different sites, many of which are cell cycle related (Drogen *et al*, 2000; Gruhler *et al*, 2005; Holt *et al*, 2009). Among the sites detected in our dataset, Ste20_S195 was affected only by NaCl and Ste20_T573 only by pheromone, while the majority of the sites were affected by both stimuli with a Shape Effect following pheromone and an Intensity Effect following NaCl stimulation (Ste20_T203_T207, Ste20_S418, and Ste20_T511). Of note, Ste20_S195 was only affected by NaCl and followed the spike-like behavior typical of Hog1 (Supplementary Fig S4A), while Ste20_T573 showed a bi-phasic pattern following pheromone treatment with peaks at 5′ and 20′ (Supplementary Fig S4B). Ste20_T203_T207 was up-regulated by NaCl, and long pheromone stimulation had the effect of accelerating this up-regulation (Supplementary Fig S4C). Finally, Ste20_T511 followed the same behavior of ppFus3 (Fig5C and 5D), both along the NaCl and the pheromone timelines (Supplementary Fig S4D and E). Similar to ppFus3, Ste20_T511 was first up-regulated and then immediately down-regulated by NaCl when the pheromone pre-stimulation lasted minimally 5′.

Another interesting case is Pbs2. Among the P-peps we could detect, Pbs2_68 was twice up-regulated by pheromone, at 1′ and at 20′, and did not appear to be affected by NaCl (Supplementary Fig S5A), while Pbs2_S269 was only affected by NaCl and followed the spike-like dynamics of the main HOG pathway P-peps (Supplementary Fig S5B). Lastly, Pbs2_S248 was affected by both signals with a Shape Effect (Supplementary Fig S5C and D).

These data show that different P-sites are reacting to the stimuli in different ways. How different phosphorylation configurations and dynamics affect the signal transmission and the crosstalk remains to be further investigated.

### Quantification of the NaCl- or the pheromone-induced influence on the P-pep behavior

**Figure 6:**
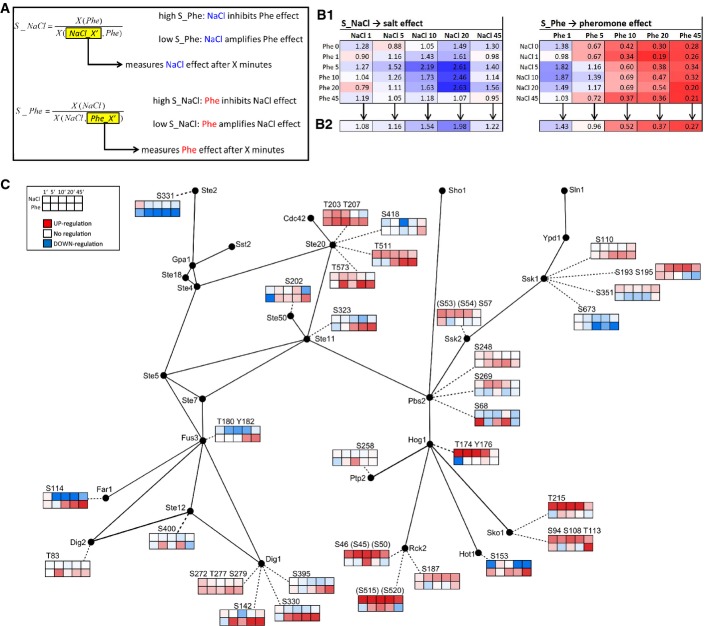
Quantification of the NaCl- and the pheromone-induced influence on each phosphopeptide

To better understand how and to what extent each stimulus affected each specific P-pep both within the known pathways and from the opposite stimulus, we computed the specificity for each stimulus and each peptide (Schaber *et al*, 2006). In this context, the term specificity is defined as the ratio between the response of a P-pep to Stimulus_1 alone (i.e. the time-resolved dynamic of a P-pep when only Stimulus_1 is being applied) and the response of the same P-pep to the combination of Stimulus_1 and Stimulus_2. A specificity ratio below 1 indicates that Stimulus_2 amplifies the effect of Stimulus_1. A ratio above 1 indicates that Stimulus_2 inhibits the effect of Stimulus_1. If the ratio is around 1, then Stimulus_2 has no significant influence on the effect of Stimulus_1 (Fig6A). For each P-pep, we therefore computed two specificity matrixes, one for each stimulus (Fig6B.1). We then collapsed the specificity matrixes by computing the averages of the specificity measures column-wise, thus obtaining 2 specificity vectors for each stimulus (Fig6B.2). The specificity for NaCl (S_NaCl) measures the effect of NaCl over a pheromone-induced response, while the specificity for pheromone (S_Phe) measures the effect of pheromone over a NaCl-induced response. As specificity vectors retain the main information provided by the specificity matrices, we chose to report all the most significant results in Fig6C as specificity vectors, while all specificity matrices are reported in Supplementary Table S3.

- Implementation of the specificity measure (Schaber *et al*, 2006) for the quantification of the NaCl- and the pheromone-induced effects over the HOG and pheromone pathway phosphopeptides (P-peps). We named the resulting specificity measures S_NaCl and S_Phe, respectively.
- For each P-pep, we computed both the matrix (B.1) and the vector (B.2) of both S_NaCl and S_Phe. Each value of the matrix was computed as explained in (A). Each value of a vector corresponds to the average of the values of the corresponding matrix taken column-wise: Each value of the vector S_NaCl, for instance, is the average of all the matrix values corresponding to a single time after NaCl stimulation.
- Representation of the most significant specificity vectors for the HOG and pheromone pathways. On top, S_NaCl is shown, and on the bottom S_Phe.

### The activation of Hog1 is subject to complex modulation by both pheromone and NaCl

**Figure 7:**
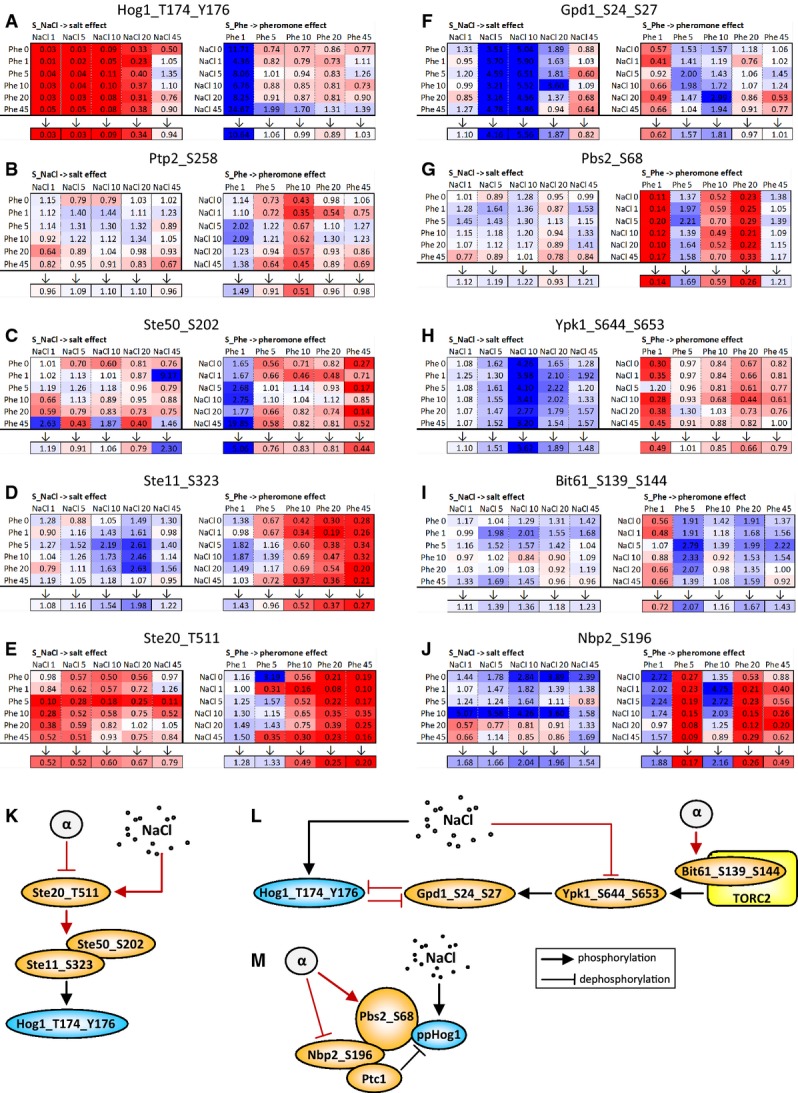
Modulation of Hog1 activation by pheromone and NaCl

The down-regulation of ppHog1 (Figs5B and 7A) could have two sources: Either a Hog1 inhibitory component is activated or an activating component is down-regulated. Using the specificity matrices, we could now search our dataset for components that either directly or inversely follow the ppHog1 dynamic pattern. In the following, we present two possibilities that account for how pheromone could inactivate ppHog1.

- A–J Specificity matrices of the following phosphopeptides: (A) Hog1_T174_Y176, (B) Ptp2_S258, (C) Ste50_S202, (D) Ste11_S323, (E) Ste20_T511, (F) Gpd1_S24_S27, (G) Pbs2_S68, (H) Ypk1_S644_S653, (I) Bit61_S139_S144, and (J) Nbp2_S196.
- K–M Predicted mechanism for the cross-modulation of Hog1 double-phosphorylation by the co-stimulation with NaCl and pheromone. The connections that are known from already published studies are indicated in black; our predicted connections are in red.

First, we looked for P-peps among the pathway components (excluding the Hog1-targeted transcription factors) that display the same phosphorylation pattern as ppHog1 within the first 10′ of pheromone stimulation. The only P-peps with similar behavior to ppHog1 were Ptp2_S258 (Fig7B), Ste50_202 (Fig7C), Ste11_S323 (Fig7D), and Ste20_T511 (Fig7E and Supplementary Fig S4D and E). Of these, Ptp2 is a nuclear tyrosine-phosphatase known to down-regulate Hog1 (Wurgler-Murphy *et al*, 1997). However, Ptp2 regulation is unknown. We find that NaCl has little if any influence on Ptp2_S258 phosphorylation, while pheromone down-regulates this site (Figs4C and 7B). In particular, a 1′ long pheromone stimulation down-regulates Ptp_S258, following a pattern that is similar to the one observed for ppHog1. This suggests that Ptp2_S258 is not targeted by Hog1 but rather by a pheromone-dependent signal. It is thus possible that the observed pheromone-induced down-regulation of Ptp2_S258 activates Ptp2, thus leading to Hog1 dephosphorylation. Among the other P-peps that mimicked the ppHog1 specificity pattern, Ste50_S202 is most strongly affected by the 1′ pheromone treatment, and Ste20_T511 appears to be significantly affected by both NaCl and pheromone and might be therefore the key P-pep, among all the P-peps of Ste20, mediating the crosstalk between the two stimuli.

We next searched for P-peps that displayed the opposite phosphorylation pattern as ppHog1: up-regulated 1′ after pheromone stimulation and then immediately down-regulated. In doing so, we found that Gpd1_S24_S27 (ppGpd1) displayed this pattern (Fig7F), while Pbs2_S68 does so only during the first 5′ (Fig7G).

As Hog1 is the main known mediator of the osmo-sensing response, we wanted to differentiate Hog1-dependent phosphorylation events from those independent of Hog1. For this, we performed a time course experiment in a yeast strain where the gene coding for Hog1 had been replaced by an inhibitable ATP-analog-sensitive version, Hog1-as (Shokat & Velleca, 2002). We observed that after Hog1-as inhibition, ppGpd1 was twofold up-regulated while the subsequent NaCl stimulation induced a quick down-regulation of the respective phosphorylation event (Supplementary Fig S6A). This result indicates that ppHog1 basal activity is necessary to maintain Gpd1 in a dephosphorylated state and therefore active at a basal level. NaCl stimulation, however, induces a fast down-regulation of ppGpd1. This process was shown to be catalyzed by the two TORC2-dependent kinases, Ypk1 and Ypk2, by Lee *et al* (2012) who showed that NaCl inhibits Ypk1/2 phosphorylation thus down-regulating ppGpd1. In our data, Ypk1_S644_S653 appears to be also briefly up-regulated 1′ after pheromone stimulation and then mildly down-regulated (Fig7H). As Ypk1/2 are phosphorylated by TORC2, we searched for P-peps belonging to this complex that also display the same S_Phe Matrix as ppGpd1 and Ypk1_S644_S653. Such behavior was indeed observed for Bit61_S139_S144 (Fig7I), a P-pep belonging to Bit61, one of the subunits of TORC2 (De Virgilio & Loewith, 2006; Cybulski & Hall, 2009). These results indicate that pheromone promotes an early phosphorylation of certain P-sites of both Ypk1 and Bit61, while NaCl appears to have the opposite effect (Fig7H and I, Supplementary Fig S6B and C).

A similar behavior to ppGpd1 could also be observed for Pbs2_S68 in our Hog1-as experiment (Fig7G and Supplementary Fig S6D), where Pbs2_S68 down-regulation also depended on ppHog1 basal activity, while NaCl down-regulated it in a Hog1-independent way. To identify P-peps that are functionally connected to Pbs2_S68, we searched the complete dataset for P-peps that have an S_Phe Matrix that is similar or opposite to the one of Pbs2_S68, especially during the first 5′–10′ after pheromone stimulation. Twenty-five such P-peps were found, some of which have already been reported in other osmotic shock studies. Interestingly, among the peptides with an opposite behavior to Pbs2_S68, we found Nbp2_S196 (Fig7J). Nbp2 is an adaptor protein that has been shown to bind Pbs2 and to recruit Ptc1, a Ser/Thr phosphatase that down-regulates ppHog1 (Warmka *et al*, 2001; Mapes & Ota, 2004). These results show that Nbp2_S196 is down-regulated by pheromone 1′ and then again 10′ after stimulation, while Pbs2_S68 has the opposite behavior. We therefore suggest that these two P-sites are responsible for the known Pbs2 and Nbp2 functional connection.

### Mathematical modeling of the newly reported mechanisms captures signal integration dynamics

We next set out to investigate how the above described P-peps and crosstalk mechanisms are integrated in the global context of the HOG and pheromone pathways. We thus addressed the question whether the dynamic measures of the P-peps we detected are consistent both with the signaling network known to regulate the response to NaCl and pheromone (Fig1A) and with the mechanisms proposed here.

We built a dynamic mathematical model of the pathways and variations thereof to include the mechanisms proposed above. The model was implemented as a set of Ordinary Differential Equations (ODEs). Because our mechanistic understanding of the P-sites measured in this study is very limited, we used a logic-based model rather than one based on the underlying biochemical reactions. The resulting model and its variations were trained to the P-pep intensities confidently detected here (Fig2C, and Supplementary Table S1) and evaluated in terms of how well they explain the data summarized by its mean squared error (MSE). To test whether a more complex model fits the data better simply because of the higher number of parameters, we then computed the Akaike information criterion (AIC), which takes into account the performance of the model while penalizing the number of parameters (Burnham & Anderson, 2002).

The application of logic-based modeling to an MS dataset posed a number of challenges. Specifically, these are related to: (i) the complexity of the dataset, (ii) the representation of peptides with unknown biological function, and (iii) the need to develop a model at the P-pep level instead of the more established protein level. To build the model, we first selected the P-peps with the most consistent behavior. We computed the coefficient of variation of each P-pep across experiments, and we selected those with a coefficient below 25%. Subsequently, the P-peps for which 25% or more of the data points had not been detected were discarded. Next, we merged P-peps with very similar trajectories, which may have been wrongly resolved or have a redundant biological function, as they are indistinguishable to the purpose of modeling. Affinity propagation clustering revealed that a small number of P-peps indeed behaved similarly. In such cases, the P-pep with previously known function was selected. If a cluster of similar P-peps consisted exclusively of previously unknown members, a cluster representative was chosen. This filtering process rendered a final dataset of 33 P-peps. The full list of P-peps and the filtering criteria are summarized in Supplementary Tables S4 and S5. Subsequently, the proteins of the starting signaling network (Fig1A) were replaced by these P-peps. If two interacting proteins were replaced by multiple P-peps each, all the possible combinations of interactions were therefore implemented. We thus obtained a “state-of-the-art” logic model of 45 nodes, that is, 33 measured P-peps (mostly with unknown function) and 12 proteins which could not be detected in a high enough number of time points, and 93 interactions (Supplementary Fig S7A and B).

**Figure 8:**
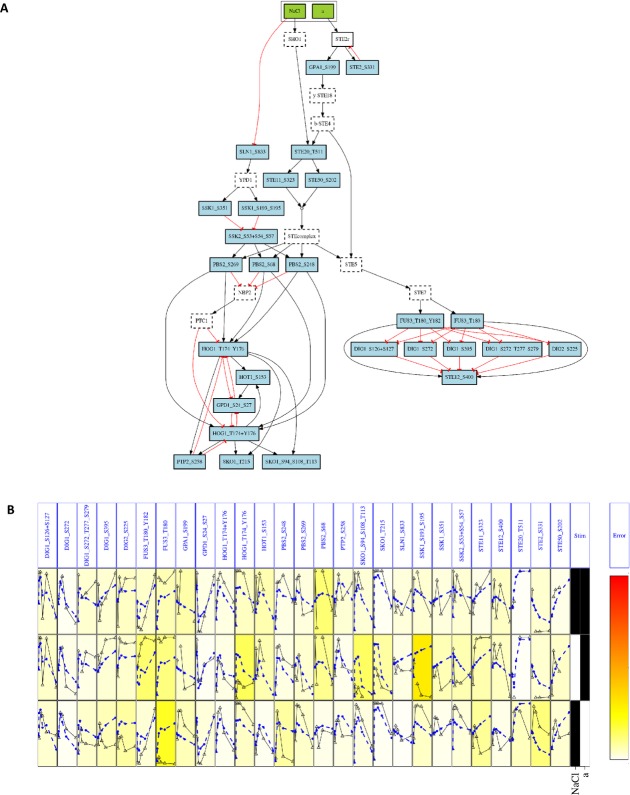
Hypothesis validation by logic modeling

To test some of the novel mechanisms described above, we then developed a set of modified versions of our logic model either by implementing Ste20_T511 as the main Ste20 P-pep mediating crosstalk (Fig7E), and/or by introducing the double-negative inhibition between ppGpd1 and ppHog1. The model including both proposed mechanisms consisted of 39 nodes and 73 interactions (Fig8A). Since a common formalism to build dynamic models is ODEs (Kholodenko *et al*, 2010), we next transformed our logic models into logic ODEs (Wittmann *et al*, 2009) by means of CellNOpt (Terfve *et al*, 2012). For details regarding P-pep modeling, please see Materials and Methods. We then trained all the resulting models, within CellNOpt, to the three time course data corresponding to the stimulation with NaCl only, pheromone only, and both stimuli at the same time, that is, the first row, the first column, and the diagonal of the stimulation matrix (Fig1B), respectively.

Logic modeling shows that the detected phosphopeptides (P-peps) and proposed crosstalk mechanisms orchestrate the response to NaCl and pheromone stimulation. The protein signal integration network shown in Fig1A was transformed into a P-pep logic model (Supplementary Fig S8).

- An ensemble of 23 model variants was generated to include the novel mechanisms proposed above, here showing the model including Ste20_T511 as the main Ste20 P-pep regulating crosstalk and the ppHog1–ppGpd1 double inhibition.
- The proposed model accurately simulated the dynamic behavior of a large number of the detected P-peps, thus supporting the proposed mechanisms (normalized measurements in black, simulations in blue, and MSE disagreement between data and simulation in background color).

We used our models to assess the likelihood of the proposed novel mechanisms based on the experimental data. Our results show that the model, extended with our proposed mechanisms (Fig8B), performs better than the prior knowledge-based one shown in Supplementary Fig S7. Specifically, with respect to the state-of-the-art model (MSE = 0.06, AIC = −837), by reducing the crosstalk mediators to only Ste20_T511, we observed no fitness loss (MSE = 0.059) and a large improvement in AIC (AIC = −955). This result suggests that Ste20_T511 indeed mediates the crosstalk, while all the other P-peps of Ste20 are non-essential crosstalk mediators under these stimulatory conditions. The further addition of the ppGpd1–ppHog1 reciprocal inhibition mechanism also showed no significant increase of fitness (MSE = 0.059) and, accordingly, a slight decrease in AIC (AIC = −937) due to the extra complexity. Since the analysis of the specificity matrices (Fig7F) indicates that ppGpd1 and ppHog1 are involved in each other's down-regulation, these two observations suggest that this feedback loop might be enhancing the signaling integration at the data points excluded from models training (i.e. co-stimulation by both NaCl and pheromone, but not simultaneous).

Finally, we assessed whether certain co-stimulated P-peps do not affect the shape of the dynamic curves. We therefore generated models where the interactions labeled as “No Effect” upon a specific stimulation (Fig4C), if present in the stimulated pathway according to literature, were removed. This amounts to removing from the model shown in Fig8A the interactions that have No Effect upon NaCl stimulation, namely the interactions between Sho1 and Ste20_T573, Ssk2_S53_S57 and Pbs2_S68, the Ste11_Ste50_complex and Pbs2_S269, and the singly and doubly phosphorylated forms of Hog1 and Ptp2_S258. We tested the effect of removing these four interactions in all possible combinations, with the model shown in Fig8A, by developing four additional models. Compared to the model shown in Fig8A, the model exhibited a loss of accuracy (MSE = 0.099, AIC = −714), indicating that at least one of the removed interactions indeed played an important role in the network. We therefore generated a final set of 15 models by removing all single interactions, one at a time. We observed that in all the models with a loss of performance, the interactions between Hog1 and Ptp2 had been removed. This indicates that, as previously known, the regulation of Hog1's phosphorylation by the phosphatase Ptp2 is essential also during NaCl stimulation. We therefore suggest that, according to our data, the interaction between Hog1 and Ptp2 is mediated by the phosphorylation of Ptp2 at Ser258.

Altogether, we investigated an ensemble of 23 different models with all possible combinations of our proposed mechanisms. The ability of the model represented in Fig8A to reproduce the data trend for most of the measured P-peps (Fig8B) suggests that the signal propagation and crosstalk, upon NaCl and pheromone stimulation, are indeed mediated by an important number of P-peps. The performance of the full ensemble of models is summarized in Supplementary Fig S8 and in Supplementary Table S6. The main models, processed and filtered data, estimated parameters, and documented scripts are available online at http://www.cellnopt.org/data/yeast/.

## Discussion

We investigated the mechanisms governing yeast cell signaling pathway interactions by measuring the phosphorylation dynamics of two interconnected signal transduction cascades, the HOG and the pheromone pathways. The method proved robust, reproducible, and accurate for the P-peps detected by our quantitative label-free shotgun proteomic strategy. We could confidently detect P-peps from 82% of proteins in the HOG or the pheromone pathways. We were also able to identify several additional proteins that, to our knowledge, have not yet been associated with either of these MAPK cascades. A fraction of the P-sites of the HOG and pheromone pathways that was identified by previous studies was not detected. One reason for this could be that the protein digestion employed for sample preparation generated peptides that were either too low or too high in mass-to-charge ratio to be detected by the mass spectrometer. Also, the very nature of a shotgun approach is such that only the most frequently detected peptide ions can be sequenced and therefore annotated. Such shortcoming of this approach was, however, largely compensated by the measurement of a large number of novel P-sites that we then showed to be involved in the signaling integration.

Given the complexity of the system and the dimensions of our global phosphoproteome dataset, in this work, we restricted our analyses to those P-peps that are known proteins of either the HOG or the pheromone pathways. We applied exploratory computational tools to: (i) identify which P-peps are significantly responding to a specific stimulus and to (ii) quantify the effect of a stimulus on the dynamic response of the detected P-peps. These detailed time-resolved and quantitative data allowed us to connect specific sets of P-sites that are likely to be involved in signal transmission and integration within and between the two stimuli response mechanisms. As a result, we find that the HOG and the pheromone pathways are much more extensively interconnected than previously thought. Based on our findings, we were able to generate new hypotheses for how pathway component interactions allow faithful signal transmission and integration, which were supported by a mathematical model of the underlying pathways. Our findings prompt specific testable questions for follow-up functional analysis.

### P-sites within the same protein show distinct and specific responses to stimulus

Our data highlight how most P-sites play unique roles in the control of each protein function to convey a specific signal to subsequent targets. Some proteins with multiple P-sites showed a complex phosphorylation response when stimulated by NaCl or pheromone individually, or when co-stimulated. As an example, let us consider Ste20, a shared component between the HOG and the pheromone pathways. The majority of the Ste20 P-peps that we could detect responded to both stimuli, but with varying dynamics. Moreover, our clustering analysis showed that P-peps of Ste20 fell into clusters representing distinct response dynamics. These combined results identify Ste20 as one of the key proteins regulating the cross-modulation between the two pathways. Another interesting finding was the unexpected involvement of certain Pbs2 and Ssk1 P-peps in both the HOG and the pheromone pathways responses. Pbs2 and Ssk1 have been described as exclusive components of the HOG pathway, and yet, we observed a significant phosphorylation response to pheromone stimulation as well. These results suggest that, besides Ste20, also Pbs2 and Ssk1 may be important links between the two inputs, thus allowing for pheromone-induced modulation of the HOG response. Taken together, our data illustrate how proteins with multiple P-sites that are differentially phosphorylated upon varied stimuli are capable of connecting and modulating two or more converging signals, allowing for signal integration within the cell signaling network.

### ppFus3 has a biphasic response to NaCl stimulation

Since hyper-osmotic shock is a life-threatening condition, cells need to prioritize the HOG pathway response over less urgent signals. For example, the activation of the pheromone MAPK, Fus3, is known to be down-regulated by NaCl (Patterson *et al*, 2010). With our data, we could indeed observe a pattern of down-regulation in ppFus3. However, since our method allows for detailed, quantitative, time-resolved phosphorylation profiles, we could refine the description of the response. In pheromone pre-stimulated cells, ppFus3 phosphorylation was increased by NaCl stimulation within the first minute, and then, phosphorylation was down-regulated after 1′ (Fig5C). This biphasic phenomenon is probably due to the NaCl-induced activation of the shared components. Once the pheromone pathway's machinery has been assembled and recruited to the membrane by pheromone pre-stimulation, NaCl stimulation further activates Ste20, Ste11, and Ste50, thus boosting Fus3 phosphorylation before the NaCl-induced ppFus3 down-regulation is triggered. How ppFus3 is down-regulated by the HOG pathway is still unclear. However, as ppFus3 down-regulation happens 1′ after NaCl stimulation when ppHog1 reaches its maximum intensity, we speculate a role for ppHog1 enzymatic activity. Indeed, Patterson *et al* (2010) showed that Hog1 inhibition, following osmotic shock by 1 M sorbitol stimulation, induces the activation of the pheromone pathway by crosstalk, which supports our hypothesis.

### Hog1 phosphorylation is transiently down-regulated by pheromone

Surprisingly, we observed that pheromone treatment induces down-regulation of ppHog1. We are aware of only one report of pheromone-induced ppHog1 down-regulation by Yamamoto *et al* (2010), who observed that long pheromone pre-stimulation (44′) followed by a 6′ 0.4 M NaCl stimulation leads to a significantly reduced ppHog1 up-regulation. While we also observed a down-regulation in Hog1 phosphorylation in similar conditions (after 45′ of pheromone and 5′ of NaCl stimulation (Figs5B and 7A)), we unexpectedly found ppHog1 to be strongly and transiently down-regulated after only 1′ of pheromone stimulation (Figs5B and 7A). This down-regulation is greater than that observed with 45′ prolonged pheromone stimulation. We therefore used our specificity matrices to identify those P-peps whose behavior can be functionally linked to ppHog1.

### Shared pathway components modulate MAPK activation

Several P-peps displayed a phosphorylation response that correlated with ppHog1, including Ste20_T511, Ste11_S323, and Ste50_S202. These P-peps are from proteins known to interact and to be shared between the two pathways. Ste50, for example, has been shown to play an important role in negative feedback control and in ppHog1 down-regulation (Yamamoto *et al*, 2010; Nagiec & Dohlman, 2012). Fus3, Hog1, and Kss1 are known to phosphorylate and inactivate Ste50, suggesting a molecular link between the HOG and the pheromone pathways.

This early pheromone-induced down-regulation of the shared MAPK cascade components may be important for the cell to prioritize responses. Since the HOG pathway activation is much faster, shorter lived, and for survival purposes more urgent than mating, pheromone could induce a short-lived down-regulation of Ste20_T511 and subsequently affect all of the downstream components of the pheromone pathway. The resulting delay in the activation of Fus3 would allow for the HOG pathway response to fully initiate (Fig7K). Since Ste20_T511 down-regulation occurs both with and without NaCl stimulation, we can deduce that pheromone always delays Fus3 activation without compromising the pheromone response. In this study, we included a modeling effort to assess how the mechanisms here presented could integrate the responses to NaCl and pheromone stimulation. While this proved to be informative when comparing models representing different variants of those mechanisms, we anticipate that further insight will be gained by extending the optimization procedure to include the data measured upon varying combinations of length in NaCl and pheromone stimulation.

### Gpd1 and Hog1 promote their mutual inhibition

The primary and quickest negative feedback mechanism predicted by Schaber *et al* (2012) involves the glycerol production machinery that is available in cells before osmotic shock, which they believe to be regulated at post-translational level. Such mechanism would promote a down-regulation of Hog1's activity that is inversely proportional to the amount of the already available glycerol-producing machinery. Gpd1, whose transcription is induced by active Hog1, catalyzes glycerol production in response to osmotic stress, and it is inactivated by phosphorylation at S24 and S27 (Oliveira *et al*, 2012). Within our specificity matrices dataset, ppGpd1 had a response opposite to ppHog1 with pheromone stimulation (Fig7F S_Phe). Consequently, ppGpd1 may be responsible for the early ppHog1 down-regulation. We also observed that ppGpd1 is down-regulated by both NaCl and ppHog1 in two partially independent ways (Fig7F S_NaCl and Supplementary Fig S6A), which quickly boost the activity of the available glycerol production machinery. Interestingly, the P-pep Ypk1_S644_S653 of a kinase that is known to phosphorylate Gpd1 also behaves like ppGpd1 during NaCl and pheromone stimulation and may be functionally linked to ppHog1.

All together, these observations lead to a few possible hypotheses. First, ppGpd1 promotes the down-regulation of ppHog1 by reducing the amount of active Hog1. Second, both NaCl and ppHog1 promote the dephosphorylation of ppGpd1. Third, while pheromone promotes an early phosphorylation of Ypk1, NaCl causes its de-phosphorylation, thus antagonizing the Ypk1 regulatory effect. Ypk1_S644_S653 is down-regulated by NaCl so that it cannot inactivate Gpd1 in osmo-stress conditions, whereas it is up-regulated by pheromone to increase the modulating activity of Gpd1 over ppHog1. In its doubly phosphorylated form, Gpd1 is incapable of catalyzing glycerol production, but it may be able to indirectly promote ppHog1 down-regulation instead. The mechanisms through which this is achieved, specifically the question which phosphatase(s) perform(s) the actual dephosphorylation, need to be further investigated. This regulatory mechanism is summarized in Fig7L.

### The TORC2 pathway modulates Hog1 activity in response to pheromone stimulation

As Ypk1/2 are known targets of TORC2, we looked within our specificity matrix dataset for TORC2 subunits that may also be functionally linked to ppHOG1 activity based on similarities of their dynamic phosphorylation patterns. We found that Bit61_S139_S144 is up-regulated after 1′ of pheromone stimulation before being down-regulated immediately afterward, like Ypk1_S644_S653, and ppGpd1. Also, Bit61_S139_S144 was found to be down-regulated by NaCl stimulation in a Hog1-independent way. These results, together with our observations relative to Ypk1_S644_S653, suggest a close link between the TORC2, the HOG, and the pheromone pathways.

The TORC2 pathway is mainly known to regulate actin polarization (De Virgilio & Loewith, 2006), even though its function is still not as clear as that of TORC1. As actin polarization is fundamental for the mating response (Ayscough & Drubin, 1998), it is no surprise that pheromone may also affect the TORC2 pathway. On the other hand, the NaCl-induced down-regulation clearly observed only in our Hog1-as experiment (Supplementary Fig S6) may be due to a crosstalk between the HOG and the pheromone pathways, which would have normally been prevented by ppHog1. We therefore suggest that pheromone affects the activity of the TORC2 pathway via the upstream components of the pheromone pathway (Fig7L) in order to initiate the TORC2-induced actin polymerization necessary for the mating response.

### A role for phosphatases in Hog1 down-regulation

The previously discussed mechanisms offer new insights into the combined NaCl–pheromone regulation of Hog1 activity, but the components responsible for dephosphorylating this kinase are unknown. Ptp2, a phospho-Tyr-specific protein phosphatase, is one of the phosphatases that are known to regulate the MAPK activity (Wurgler-Murphy *et al*, 1997). In our study, we measured the activity of one of its P-peps, Ptp2_S258, which also behaves like ppHog1 (Fig7B). We therefore suggest that one of the means for ppHog1 down-regulation 1′ after pheromone stimulation is the pheromone-induced short-lived activation of Ptp2 by its dephosphorylation at S258.

Ptc1 is a phospho-Ser/Thr-specific phosphatase, which is known to bind Pbs2 through the adaptor protein Nbp2 to down-regulate ppHog1 and to be regulated by pheromone stimulation (Malleshaiah *et al*, 2010). Within the first 5′ of pheromone stimulation, Nbp2_S196 behaves like ppHog1, while Pbs2_S68 shows the opposite behavior. Additionally, our Hog1-as time course shows that NaCl-dependent down-regulation of Pbs2_S68 depends on Hog1 activity. Even though Ptc1 was not detected in our experiment, the behavior of Nbp2_S196 and Pbs2_S68, together with the established knowledge of their interaction with Ptc1, lead us to propose that pheromone could exert a negative regulation of Hog1 through the Ptc1 phosphatase (Fig7M).

### Concluding Remarks

Like every known signaling pathway, the HOG and the pheromone pathways are activated by two specific signals that trigger two particular responses. Every pathway, however, consists of a set of components that ultimately belong to the large pool of cell molecules that, through complex functional interconnections, comprise the cell signaling network. Pathways operating in such a complex environment cannot be considered as isolated signaling units. Aside from the most direct responses to the triggering stimuli, we can expect pathways to also react in less obvious ways to multi-stimulations and to stimuli recognized by receptors of other pathways. Such a scenario is important for cell survival, since all the cell's actions must take into account all the available information regarding their environment (nutrients, oxygen, stress/harm conditions, signals sent by other cells, etc.) in order for the cell to decide on an optimal overall response.

In our study, osmo-stress is a high-priority issue that threatens survival. Cells need to react fast, also by preventing, delaying, or just down-regulating other cellular processes that are less urgent or that require safe conditions to be completed—including DNA replication during cell budding or pheromone response. Hence, the core HOG pathway quickly activates or deactivates a set of components in order to prevent cell death and, at the same time, to inhibit or modulate other cellular responses. Pheromone activation, in turn, also affects several processes of the cell (e.g. progression through the cell cycle and cytoskeleton reorganization during shmooing) (Dumont *et al*, 2001). Since one of the first stages of the mating response to pheromone signaling is shmooing, which consists in the formation of a long cellular bulge, the cell wall integrity pathway is consequently also activated (Buehrer & Errede, 1997; Baltanas *et al*, 2013), and the cytoskeleton needs to be thoroughly reorganized, thus demonstrating yet another example of the integration within the overall cell signaling network.

The framework of defining specific signaling cascades as isolated pathways has certainly proven to be a useful simplification of the cell circuitry when studying the response to a particular input stimulus. However, to investigate the complex signaling integration used by cells to process multi-faceted environmental stimuli, and to therefore understand the underlying physiological and pathological mechanisms, we need to extend our analysis to the whole cell signaling network.

## Materials and Methods

### Yeast strain, cell cultures, and stimulation

The *Saccharomyces cerevisiae* strain used for all the double time course experiments was a BY4741 with a MATa cdc28::KanMX + pJU1203 (pRS 416; CDC28as1 = F88G) LYS2-met15 genotype, which is provided with a Cdc28-as allele that can be inhibited by means of 1-NA-PP1, the ATP analog “PP1 analog 8” (D'Aquino *et al*, 2005). The efficiency of this inhibitor on Cdc28-as was preliminarily tested on our cells by halo assay.

Cells were grown in 50 ml SD medium, within 500-ml shaking flasks, at 30°C, to an OD_600_ of 0.6 (exponential growth). The ATP analog 1-NA-PP1 was then added to all cultures to a final concentration of 10 μM (from a 10 mM stock solution in DMSO). One hour after inhibition, cells were stimulated according to the stimulation matrix (Fig1B): NaCl was added to 0.4 M final concentration (from a 4 M NaCl stock solution in SD medium), α-factor to a final concentration of 1 μM (from a 5 mg/ml stock solution in DMSO). Cell biochemical activities were quickly arrested by the addition of ice-cold 100% (w/v) trichloroacetic acid, to a final concentration of 6.25%, directly into the cell cultures. After 30′ of TCA incubation on ice, cell pellets were washed twice with 10 ml ice-cold acetone and then stored at −80°C after complete acetone removal.

For every NaCl/pheromone stimulation time point (i.e. for each square in the matrix shown in Fig1B), three biological replicates were prepared. For practical reasons, the complete matrix experiment was split into six sub-experiments, each one covering only one row of the matrix.

### Protein extraction, enzymatic digestion, and phosphopeptide enrichment

Cells were lysed by bead-beating. Acid-washed glass beads were added to the pellet in an amount equal to the pellet itself (about 250 μl). Each cell pellet was then re-suspended into 400 μl of a buffer consisting of 8 M urea, 50 mM ammonium bicarbonate, and 5 mM EDTA. Bead-beating was performed for 5′ at 4°C, for four times, thus producing 1.6 ml cell lysate.

Protein concentration was measured by BCA assay. For each biological replicate, 3 mg of total protein was reduced by 5 mM TCEP (45′), alkylated by 12 mM iodoacetamide (1 h), and then digested overnight by trypsin (1:125 w/w). Peptides were then cleaned by reverse phase chromatography. P-pep isolation was performed by titanium dioxide resin (GL Science), 1.25 mg resin for each sample. P-peps were then again cleaned by reverse phase chromatography. The detailed procedure has been thoroughly described by Bodenmiller and Aebersold (2010).

### MS analysis

All the P-pep-enriched samples were analyzed on a hybrid LTQ-Orbitrap XL (Thermo Scientific), a high mass-accuracy, and high sensitivity mass spectrometer, interfaced with a nano-electrospray ion source. In order to reduce the effect of physiological variability on the mass spectrometric measurements, the samples were analyzed in batch, using the same liquid chromatography (LC) system so as to ensure a good reproducibility of the chromatographic retention times. A 90-min gradient (starting with 3% and ending with 23% acetonitrile) was used for liquid chromatography elution. The four most intense ions detected in each MS1 measurement were selected for MS2 fragmentation.

### Data analysis

The acquired data were searched against an SGD target/decoy database (Elias & Gygi, 2007) for yeast proteins using the Sorcerer Sequest version 4.2.0 search algorithm (Eng *et al*, 1994; Lundgren *et al*, 2009). Search results were evaluated with the Trans Proteomic Pipeline (Keller *et al*, 2005) using the Peptide Prophet version 4.5.2 (Keller *et al*, 2002). Based on a decoy search (Kall *et al*, 2008), maximum false discovery rate was set to 1%. OpenMS version 1.9 (Sturm *et al*, 2008) was used to detect MS1 features (sets of spectra that OpenMS recognizes as belonging to the same peptide), annotate them, and align them between the different MS runs. Probability scores from analysis of peptides by Peptide Prophet were used to filter OpenMS results at a false discovery rate threshold < 1%. Only the phosphorylated peptides were considered for further analysis. P-peps feature with identical sequence and P-sites, but different charge states, retention times, or mass-to-charge ratios were merged together (that is, their intensities were summed). P-peps with the same amino acid sequence and the same number of phosphate groups were also merged, as the MS2 spectrum of a P-pep does not always provide the information necessary to assign a phosphate to its correct P-site. Uncertain P-sites are reported within brackets, while the actual number of P-sites within each peptide can be deduced by the P-pep sequence, since only one representative sequence is reported.

All the MS intensities were normalized by the total ion current (TIC) of each MS run. As the TIC is the sum of all the intensities detected within the linear elution gradient, it accounts both for sample concentration discrepancies and for LC-MS variability. This method was chosen as, to our knowledge, it is the most unbiased. Only P-peps detected in at least one of the three biological replicates of at least four of the six NaCl and pheromone time points were further considered for the analysis. Biological replicate values were averaged to condense the dataset. Missing values were estimated by cubic spline data interpolation.

The P-peps belonging to the HOG and the pheromone pathways have been classified by a hierarchical clustering (Fraley & Raftery, 2002), using the Minkowski distance (Karakoc *et al*, 2006). This clustering analysis was performed by means of the software R (http://www.r-project.org), while all of the analyses described in the next section as well as any data (2D and 3D) representation were performed by means of MatLab version R2013 (http://www.mathworks.com).

### Shape and Intensity Effects

The P-peps NaCl time-curves and pheromone time-curves were clustered in two separate sessions. We used K-means clustering, with the Euclidean distance, in order to keep the number of clusters to a minimum. The number of clusters generated was 6 for the NaCl time-curves and 8 for the pheromone time-curves. For each P-pep, we then observed how many different clusters were assigned to its NaCl and to its pheromone time-curves. When these numbers were equal or exceeding 3, then we classified the relative behaviors as Shape Effects.

All of the P-peps whose curves belonged to < 3 clusters were further analyzed as follows. As their curves were very similar, they were averaged: For each NaCl (and pheromone, but separately) time point, the average intensity was computed. Each P-pep was then scored by subtracting the resulting minimum average intensity from the maximum one and by dividing the result by the average of all the intensities. The behavior of P-peps that scored above or equal to 0.7 was classified as an Intensity Effect.

### Phosphopeptide selection, data integration, and logic modeling

MS-DAS (https://pypi.python.org/pypi/msdas) was used to process the MS dataset and enable logic-based modeling with CellNOpt. For each P-pep, we used only the measurements acquired upon stimulation with NaCl, pheromone, and both stimuli at the same time, for a total of 16 experiments out of the 36 (Fig1B). Next, we calculated the coefficient of variation across replicates for each P-pep, selecting only those below a 0.25 threshold. P-peps for which 25% or more of the data points were missing were discarded. In the dataset used for modeling, two single data points, that is FUS3_T180_Y182 and HOG1_T174_Y176 where both pheromone and NaCl were absent, were interpolated using a cubic spline as initial conditions are necessary for modeling. All P-peps belonging to the same protein were clustered to identify redundant trajectories using affinity propagation via the scikit python tool (http://scikit-learn.org/stable/), as described in the main text. To enable modeling using CellNOpt, the data were saved in MIDAS format (Saez-Rodriguez *et al*, 2008). Finally, proteins in the logic model corresponding to Fig1A were replaced by the P-peps that passed the filtering process, and thereby, a model of the state-of-the-art role of the measured P-peps within the HOG and the pheromone pathways was assembled.

Next, we implemented a system of equations where each equation represents the level of one signaling intermediate in the model. To that end, the logic ODE approach (Wittmann *et al*, 2009) allows us to express the change over time in the normalized abundance of each P-pep as a function of its regulatory P-peps, that is, its inputs. Consider, for example, that Hot1 is phosphorylated at S153 by Hog1 doubly phosphorylated at T174 and Y176. The change over time in abundance of Hot1_S153 can be therefore represented as: 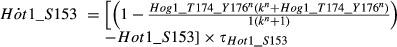
where the level of Hot1_S153 depends on the abundance of Hog1_T174_Y176 and on a degradation rate that assumes that dephosphorylation is proportional to the abundance of Hot1_S153. The parameter τ is a time-scale of the activation of Hot1_S153, and both *n* and *k* are the parameters of a Hill function for normalization.

For logic modeling, the data were normalized between 0 and 1. We used a nonlinear normalization via a Hill function with a Hill coefficient of 4. The IC50 coefficient of the Hill function was determined by selecting the middle point of the cumulative distribution function using all data points for each P-pep. This normalization prevents very large values from biasing the model. Finally, each model described in the text was fit to the normalized data using the logic ODE formalism of CellNOpt embedded in the CNORode R package available in bioconductor. As a global optimization procedure, a scatter search algorithm was used, included in the R meigor package (Egea *et al*, 2014). Each optimization problem was run for 48 h 50 times. Most cases converged on a very similar fit (Supplementary Fig S8).

For model selection, the AIC (Burnham & Anderson, 2002), a measure where higher values indicate increased information loss, was computed using the MSE as accuracy signature. To enable comparison of models where selected P-peps were removed, the standard MSE computed by CNORode (Terfve *et al*, 2012) was corrected to be calculated only in the performance of the nodes present in all models. In order to account for model fit and number of data points while penalizing an increase in the number of parameters, the AIC was defined as shown in the following equation: 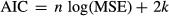
where *k* is the number of parameters and *n* the number of data points.

The logic models both in SIF Saito & Posas (2012) and in SBML-qual formats (Chaouiya *et al* 2013) are available online at http://www.cellnopt.org/data/yeast/ and in the Supplementary Model Files. The processed and filtered phosphopeptide measurements in MIDAS format, estimated parameters, and a documented script are provided as well and can be used via the CellNOptR and CNORode R packages. The models including the state-of-the-art and the mechanisms’ crosstalk, ppGpd1–ppHog1 regulation, and “No Effect”, as well as the phosphopeptide measurements selected and normalized as MIDAS, are also provided for modeling using the CellNOptR and CNOrode R packages.

### Data Availability

The matrix mass spectrometry data have been deposited to the ProteomeXchange Consortium (http://proteomecentral.proteomexchange.org; Vizcaíno *et al*, 2014) via the PRIDE partner repository with the dataset identifier PXD001445.

## Supporting Information

Supplementary Table S6

Supplementary Model Files

Review Process File

## References

[b1] Ayscough KR, Drubin DG (1998). A role for the yeast actin cytoskeleton in pheromone receptor clustering and signalling. Curr Biol.

[b2] Baltanas R, Bush A, Couto A, Durrieu L, Hohmann S, Colman-Lerner A (2013). Pheromone-induced morphogenesis improves osmoadaptation capacity by activating the HOG MAPK pathway. Sci Signal.

[b3] Bardwell L (2004). A walk-through of the yeast mating pheromone response pathway. Peptides.

[b4] Behar M, Dohlman HG, Elston TC (2007). Kinetic insulation as an effective mechanism for achieving pathway specificity in intracellular signaling networks. Proc Natl Acad Sci USA.

[b5] Binder B, Heinrich R (2004). Interrelations between dynamical properties and structural characteristics of signal transduction networks. Genome Inform.

[b6] Bodenmiller B, Aebersold R (2010). Quantitative analysis of protein phosphorylation on a system-wide scale by mass spectrometry-based proteomics. Methods Enzymol.

[b7] Buehrer BM, Errede B (1997). Coordination of the mating and cell integrity mitogen-activated protein kinase pathways in *Saccharomyces cerevisiae*. Mol Cell Biol.

[b8] Burnham KP, Anderson DR (2002). Model Selection and Multimodel Inference: a Practical Information-Theoretic Approach.

[b501] Chaouiya C, Bérenguier D, Keating SM, Naldi A, van Iersel MP, Rodriguez N, Dräger A, Büchel F, Cokelaer T, Kowal B, Wicks B, Gonçalves E, Dorier J, Page M, Monteiro PT, von Kamp A, Xenarios I, de Jong H, Hucka M, Klamt S (2013). SBML qualitative models: a model representation format and infrastructure to foster interactions between qualitative modelling formalisms and tools. BMC Syst Biol.

[b9] Choi KY, Kranz JE, Mahanty SK, Park KS, Elion EA (1999). Characterization of Fus3 localization: active Fus3 localizes in complexes of varying size and specific activity. Mol Biol Cell.

[b10] Colman-Lerner A, Gordon A, Serra E, Chin T, Resnekov O, Endy D, Pesce CG, Brent R (2005). Regulated cell-to-cell variation in a cell-fate decision system. Nature.

[b11] Cybulski N, Hall MN (2009). TOR complex 2: a signaling pathway of its own. Trends Biochem Sci.

[b12] D'Aquino KE, Monje-Casas F, Paulson J, Reiser V, Charles GM, Lai L, Shokat KM, Amon A (2005). The protein kinase Kin4 inhibits exit from mitosis in response to spindle position defects. Mol Cell.

[b13] De Virgilio C, Loewith R (2006). Cell growth control: little eukaryotes make big contributions. Oncogene.

[b14] Dohlman HG, Slessareva JE (2006). Sci. STKE.

[b15] Drogen F, O'Rourke SM, Stucke VM, Jaquenoud M, Neiman AM, Peter M (2000). Phosphorylation of the MEKK Ste11p by the PAK-like kinase Ste20p is required for MAP kinase signaling *in vivo*. Curr Biol.

[b16] Dumont JE, Pecasse F, Maenhaut C (2001). Crosstalk and specificity in signalling. Are we crosstalking ourselves into general confusion?. Cell Signal.

[b17] Egea JA, Henriques D, Cokelaer T, Villaverde AF, MacNamara A, Danciu DP, Banga JR, Saez-Rodriguez J (2014). MEIGO: an open-source software suite based on metaheuristics for global optimization in systems biology and bioinformatics. BMC Bioinformatics.

[b18] Elias JE, Gygi SP (2007). Target-decoy search strategy for increased confidence in large-scale protein identifications by mass spectrometry. Nat Methods.

[b19] Elion EA (2000). Pheromone response, mating and cell biology. Curr Opin Microbiol.

[b20] Elion EA (2001). The Ste5p scaffold. J Cell Sci.

[b21] Eng JK, McCormack AL, Yates JR (1994). An approach to correlate tandem mass spectral data of peptides with amino acid sequences in a protein database. J Am Soc Mass Spectrom.

[b22] Fey D, Croucher DR, Kolch W, Kholodenko BN (2012). Crosstalk and signaling switches in mitogen-activated protein kinase cascades. Front Physiol.

[b23] Fraley C, Raftery AE (2002). Model-based clustering, discriminant analysis, and density estimation. J Am Stat Assoc.

[b24] Gruhler A, Olsen JV, Mohammed S, Mortensen P, Faergeman NJ, Mann M, Jensen ON (2005). Quantitative phosphoproteomics applied to the yeast pheromone signaling pathway. Mol Cell Proteomics.

[b25] Hao N, Nayak S, Behar M, Shanks RH, Nagiec MJ, Errede B, Hasty J, Elston TC, Dohlman HG (2008). Regulation of cell signaling dynamics by the protein kinase-scaffold Ste5. Mol Cell.

[b26] Holt LJ, Tuch BB, Villen J, Johnson AD, Gygi SP, Morgan DO (2009). Global analysis of Cdk1 substrate phosphorylation sites provides insights into evolution. Science.

[b27] Kall L, Storey JD, MacCoss MJ, Noble WS (2008). Assigning significance to peptides identified by tandem mass spectrometry using decoy databases. J Proteome Res.

[b28] Karakoc E, Cherkasov A, Sahinalp SC (2006). Distance based algorithms for small biomolecule classification and structural similarity search. Bioinformatics.

[b29] Keller A, Nesvizhskii AI, Kolker E, Aebersold R (2002). Empirical statistical model to estimate the accuracy of peptide identifications made by MS/MS and database search. Anal Chem.

[b30] Keller A, Eng J, Zhang N, Li XJ, Aebersold R (2005). A uniform proteomics MS/MS analysis platform utilizing open XML file formats. Mol Syst Biol.

[b31] Kholodenko BN, Hancock JF, Kolch W (2010). Signalling ballet in space and time. Nat Rev Mol Cell Biol.

[b32] Komarova NL, Zou X, Nie Q, Bardwell L (2005). A theoretical framework for specificity in cell signaling. Mol Syst Biol.

[b33] Lee YJ, Jeschke GR, Roelants FM, Thorner J, Turk BE (2012). Reciprocal phosphorylation of yeast glycerol-3-phosphate dehydrogenases in adaptation to distinct types of stress. Mol Cell Biol.

[b34] Lundgren DH, Martinez H, Wright ME, Han DK (2009). Protein identification using Sorcerer 2 and SEQUEST. Curr Protoc Bioinformatics.

[b35] Malleshaiah MK, Shahrezaei V, Swain PS, Michnick SW (2010). The scaffold protein Ste5 directly controls a switch-like mating decision in yeast. Nature.

[b36] Mapes J, Ota IM (2004). Nbp2 targets the Ptc1-type 2C Ser/Thr phosphatase to the HOG MAPK pathway. EMBO J.

[b37] McClean MN, Mody A, Broach JR, Ramanathan S (2007). Cross-talk and decision making in MAP kinase pathways. Nat Genet.

[b38] Mok J, Kim PM, Lam HY, Piccirillo S, Zhou X, Jeschke GR, Sheridan DL, Parker SA, Desai V, Jwa M, Cameroni E, Niu H, Good M, Remenyi A, Ma JL, Sheu YJ, Sassi HE, Sopko R, Chan CS, De Virgilio C (2010). Deciphering protein kinase specificity through large-scale analysis of yeast phosphorylation site motifs. Sci Signal.

[b39] Mollapour M, Piper PW (2007). Hog1 mitogen-activated protein kinase phosphorylation targets the yeast Fps1 aquaglyceroporin for endocytosis, thereby rendering cells resistant to acetic acid. Mol Cell Biol.

[b40] Muzzey D, Gomez-Uribe CA, Mettetal JT, van Oudenaarden A (2009). A systems-level analysis of perfect adaptation in yeast osmoregulation. Cell.

[b41] Nagiec MJ, Dohlman HG (2012). Checkpoints in a yeast differentiation pathway coordinate signaling during hyperosmotic stress. PLoS Genet.

[b42] Oehlen LJ, Cross FR (1994). G1 cyclins CLN1 and CLN2 repress the mating factor response pathway at Start in the yeast cell cycle. Genes Dev.

[b43] Oliveira AP, Ludwig C, Picotti P, Kogadeeva M, Aebersold R, Sauer U (2012). Regulation of yeast central metabolism by enzyme phosphorylation. Mol Syst Biol.

[b44] O'Rourke SM, Herskowitz I (1998). The Hog1 MAPK prevents cross talk between the HOG and pheromone response MAPK pathways in *Saccharomyces cerevisiae*. Genes Dev.

[b45] Papin JA, Palsson BO (2004). Topological analysis of mass-balanced signaling networks: a framework to obtain network properties including crosstalk. J Theor Biol.

[b46] Parnell SC, Marotti LA, Kiang L, Torres MP, Borchers CH, Dohlman HG (2005). Phosphorylation of the RGS protein Sst2 by the MAP kinase Fus3 and use of Sst2 as a model to analyze determinants of substrate sequence specificity. Biochemistry.

[b47] Patterson JC, Klimenko ES, Thorner J (2010). Single-cell analysis reveals that insulation maintains signaling specificity between two yeast MAPK pathways with common components. Sci Signal.

[b48] Posas F, Chambers JR, Heyman JA, Hoeffler JP, de Nadal E, Arino J (2000). The transcriptional response of yeast to saline stress. J Biol Chem.

[b49] Saez-Rodriguez J, Goldsipe A, Muhlich J, Alexopoulos LG, Millard B, Lauffenburger DA, Sorger PK (2008). Flexible informatics for linking experimental data to mathematical models via DataRail. Bioinformatics.

[b50] Saito H (2010). Regulation of cross-talk in yeast MAPK signaling pathways. Curr Opin Microbiol.

[b51] Saito H, Posas F (2012). Response to hyperosmotic stress. Genetics.

[b52] Schaber J, Kofahl B, Kowald A, Klipp E (2006). A modelling approach to quantify dynamic crosstalk between the pheromone and the starvation pathway in baker's yeast. FEBS J.

[b53] Schaber J, Baltanas R, Bush A, Klipp E, Colman-Lerner A (2012). Modelling reveals novel roles of two parallel signalling pathways and homeostatic feedbacks in yeast. Mol Syst Biol.

[b54] Schwartz MA, Baron V (1999). Interactions between mitogenic stimuli, or, a thousand and one connections. Curr Opin Cell Biol.

[b55] Shokat K, Velleca M (2002). Novel chemical genetic approaches to the discovery of signal transduction inhibitors. Drug Discov Today.

[b56] Somsen OJ, Siderius M, Bauer FF, Snoep JL, Westerhoff HV (2002). Selectivity in overlapping MAP kinase cascades. J Theor Biol.

[b57] Strickfaden SC, Winters MJ, Ben-Ari G, Lamson RE, Tyers M, Pryciak PM (2007). A mechanism for cell-cycle regulation of MAP kinase signaling in a yeast differentiation pathway. Cell.

[b58] Sturm M, Bertsch A, Gröpl C, Hildebrandt A, Hussong R, Lange E, Pfeifer N, Schulz-Trieglaff O, Zerck A, Reinert K, Kohlbacher O (2008). OpenMS – an open-source software framework for mass spectrometry. BMC Bioinformatics.

[b59] Terfve C, Cokelaer T, Henriques D, MacNamara A, Goncalves E, Morris MK, van Iersel M, Lauffenburger DA, Saez-Rodriguez J (2012). CellNOptR: a flexible toolkit to train protein signaling networks to data using multiple logic formalisms. BMC Syst Biol.

[b60] Tisch D, Schuster A, Schmoll M (2014). Crossroads between light response and nutrient signalling: ENV1 and PhLP1 act as mutual regulatory pair in Trichoderma reesei. BMC Genom.

[b61] Vizcaíno JA, Deutsch EW, Wang R, Csordas A, Reisinger F, Ríos D, Dianes JA, Sun Z, Farrah T, Bandeira N, Binz PA, Xenarios I, Eisenacher M, Mayer G, Gatto L, Campos A, Chalkley RJ, Kraus HJ, Albar JP, Martinez-Bartolomé S (2014). ProteomeXchange provides globally co-ordinated proteomics data submission and dissemination. Nat Biotechnol.

[b62] Waltermann C, Klipp E (2010). Signal integration in budding yeast. Biochem Soc Trans.

[b63] Warmka J, Hanneman J, Lee J, Amin D, Ota I (2001). Ptc1, a type 2C Ser/Thr phosphatase, inactivates the HOG pathway by dephosphorylating the mitogen-activated protein kinase Hog1. Mol Cell Biol.

[b64] Westfall PJ, Patterson JC, Chen RE, Thorner J (2008). Stress resistance and signal fidelity independent of nuclear MAPK function. Proc Natl Acad Sci USA.

[b65] Wittmann DM, Krumsiek J, Saez-Rodriguez J, Lauffenburger DA, Klamt S, Theis FJ (2009). Transforming Boolean models to continuous models: methodology and application to T-cell receptor signaling. BMC Syst Biol.

[b66] Wurgler-Murphy SM, Maeda T, Witten EA, Saito H (1997). Regulation of the *Saccharomyces cerevisiae* HOG1 mitogen-activated protein kinase by the PTP2 and PTP3 protein tyrosine phosphatases. Mol Cell Biol.

[b67] Yamamoto K, Tatebayashi K, Tanaka K, Saito H (2010). Dynamic control of yeast MAP kinase network by induced association and dissociation between the Ste50 scaffold and the Opy2 membrane anchor. Mol Cell.

[b68] Yu RC, Pesce CG, Colman-Lerner A, Lok L, Pincus D, Serra E, Holl M, Benjamin K, Gordon A, Brent R (2008). Negative feedback that improves information transmission in yeast signalling. Nature.

