# Supplementary material for: Phosphoproteomic analyses reveal novel cross-modulation mechanisms between two signaling pathways in yeast
Source: Mol Syst Biol. 2014 Dec 9;10(12):767. doi: 10.15252/msb.20145112 (PMC4300490; doi:10.15252/msb.20145112)
Supplement: Supplementary file 1 [file msb0010-0767-sd1.pdf]

## Supplementary figures

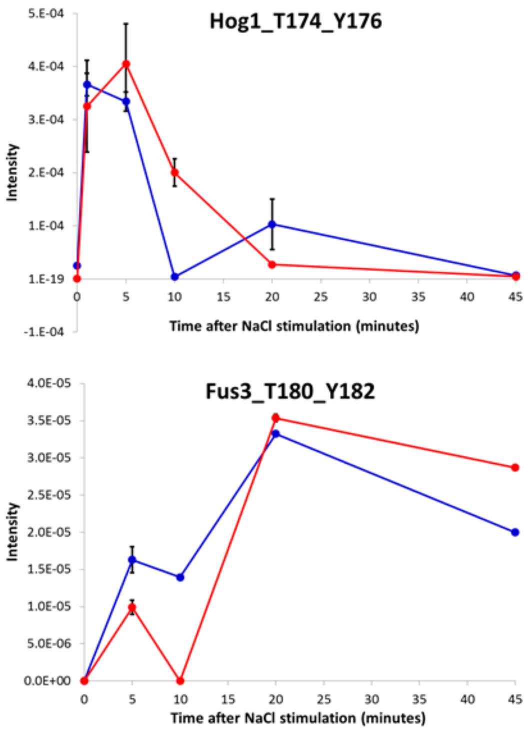


**Supplementary Figure S1** Cross-experiments reproducibility of the workflow used in this study. We show here the dynamic curves obtained, in two separate but identical time-course experiments, for Hog1_T174_Y176 after NaCl stimulation only (top), and Fus3_T180_Y182 after pheromone stimulation only (bottom). The error bars indicate the variability between the biological triplicates produced for each timepoint.

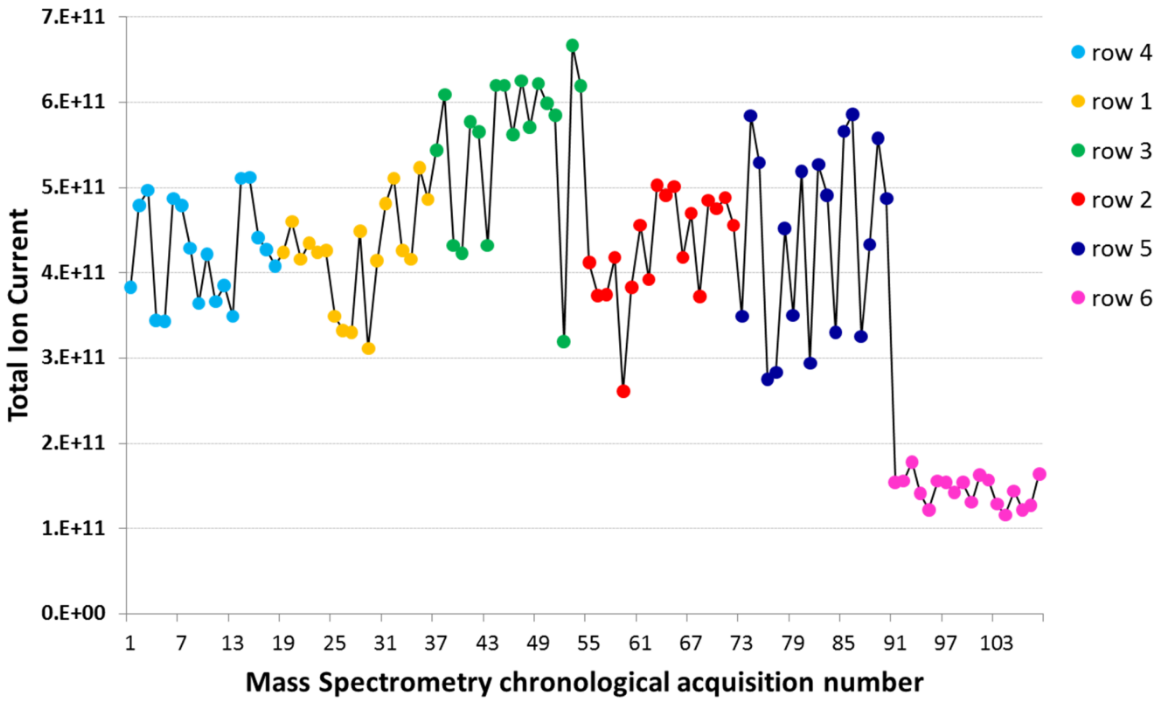


**Supplementary Figure S2** Total ion current (TIC) values, for each mass spectrometry measurement, chronologically ordered according to their acquisition time. Samples have been measured all in one batch, one row of the Matrix (Figure 1B) after the other, with the same liquid chromatography setup. By the last acquisitions (in pink), the system had lost in efficiency, and the TIC of the last measurement were thus significantly lower than the previous measurements. Other oscillation may be also due to sample preparation variability.

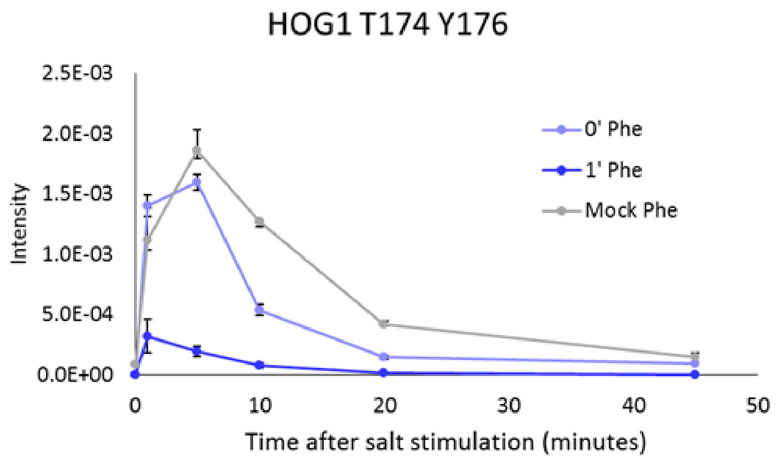


**Supplementary Figure S3** NaCl time-course results for the first (0' Phe) and second (1' Phe) rows of the Matrix, and the NaCl time-course after mock 1' pheromone stimulation for the phospho-peptide Hog1_T174_Y176.

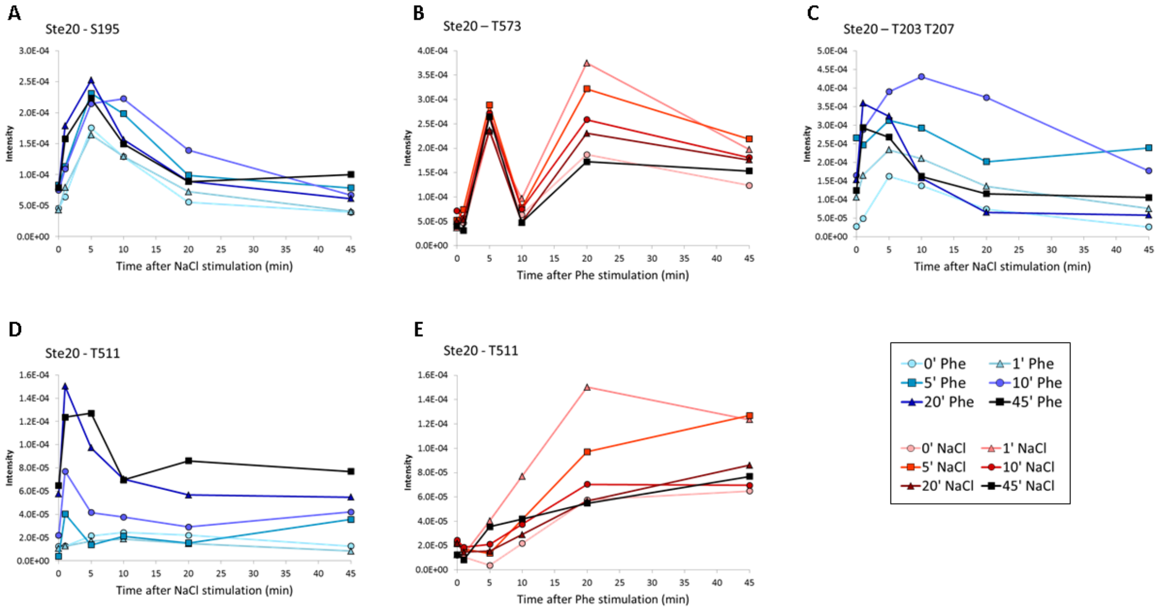


**Supplementary Figure S4** The 2-dimensional dynamics curves of some phospho-peptides of Ste20: (**A**) Ste20_S195, (**B**) Ste20_T573, (**C**) Ste20_T203_T207, (**D** and **E**) Ste20_T511.

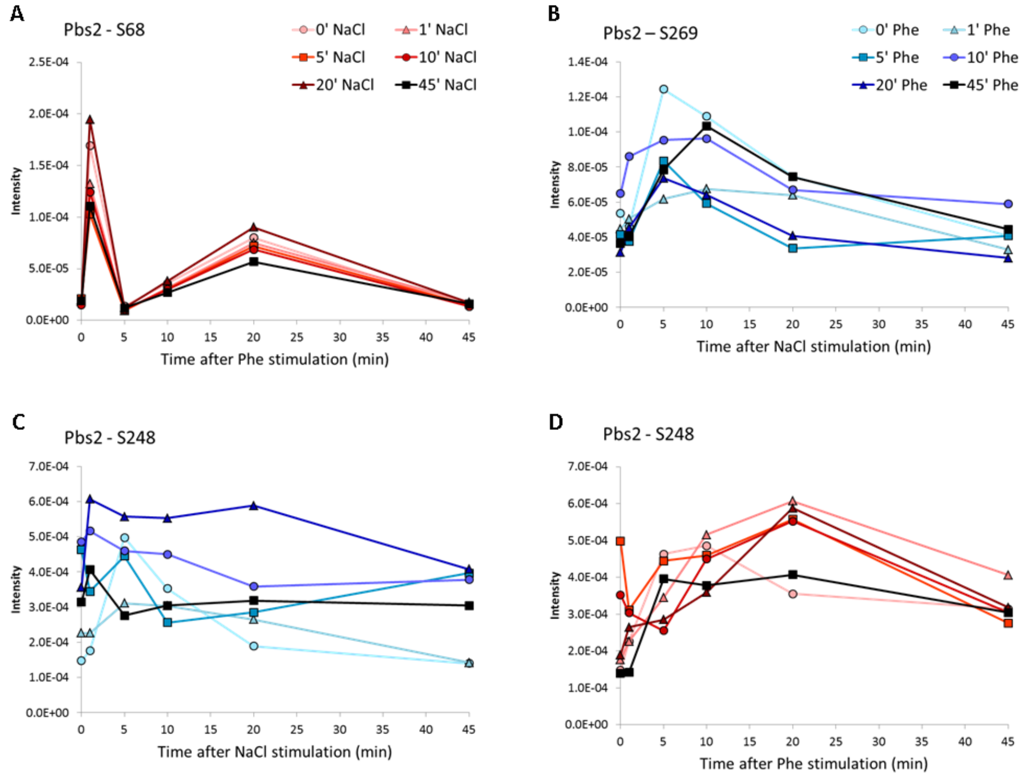


**Supplementary Figure S5** The 2-dimensional dynamics curves of some phospho-peptides of Pbs2: (**A**) Pbs2_S68, (**B**) Pbs2_S269, (**C** and **D**) Pbs2_S248.

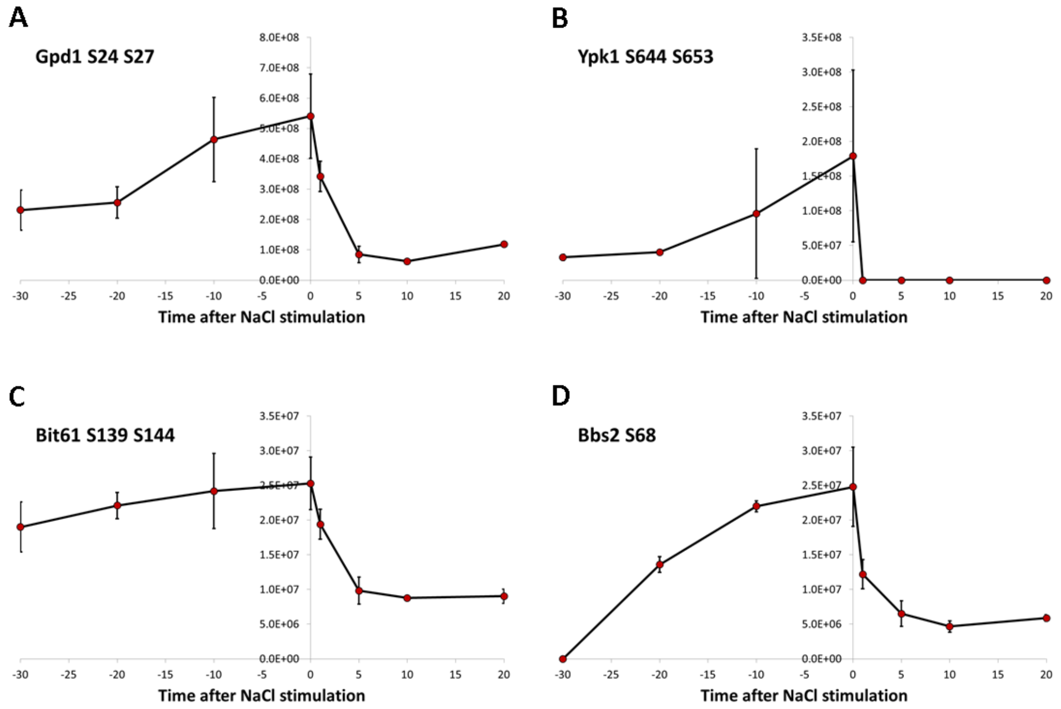


**Supplementary Figure S6** Dynamics curves of (**A**) Gpd1_S24_S27, (**B**) Ypk1_S644_S653, (**C**) Bit61_S139_S144, and (**D**) Pbs2_S68 when NaCl stimulation (at time 0) is applied 30’ after Hog1-as inhibition.

A

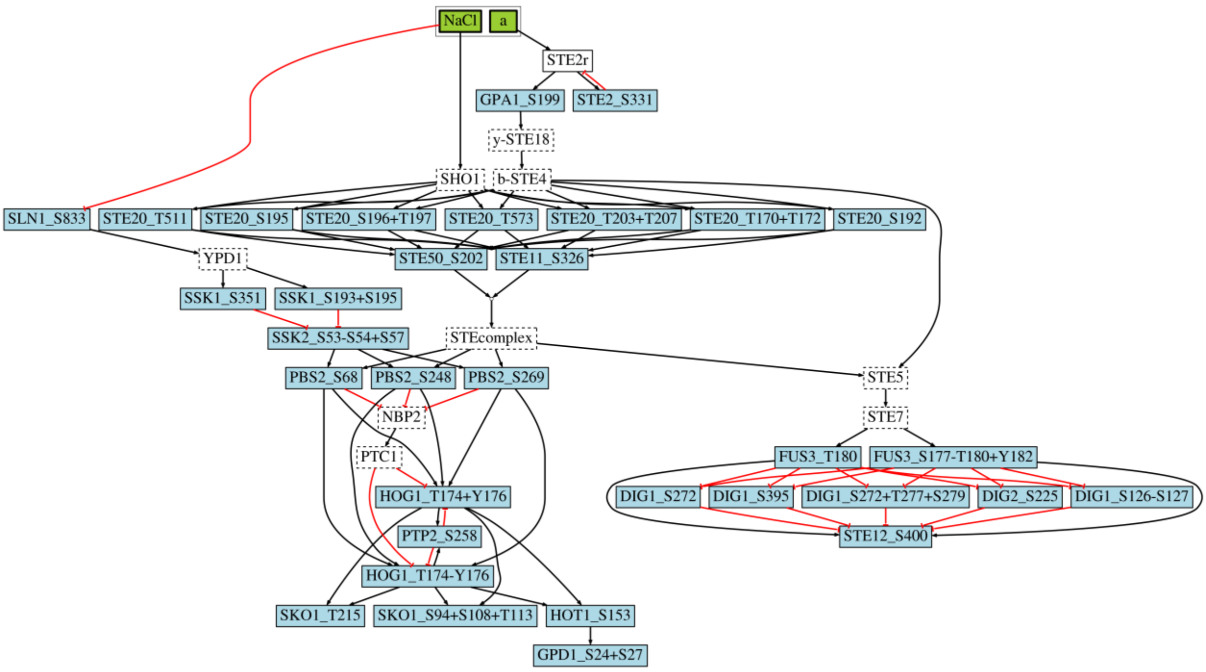


B

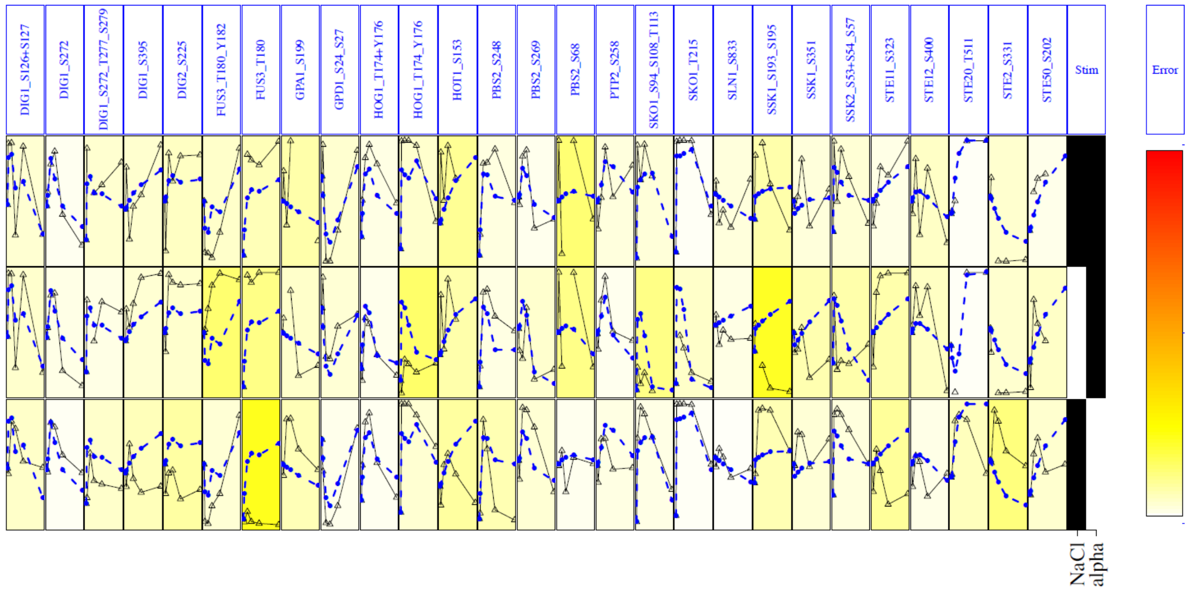


**Supplementary Figure S7** Logic model simulates HOG and pheromone signaling pathways at phosphopeptides level. (**A**) A logic model was developed using as initial hypothesis the state of the art knowledge on signal transduction and HOGpheromone crosstalk. The detected phosphopeptides were selected according to their dynamic behavior and used to replace the proteins in Figure 1A. (**B**) Simulation of the model (blue) as compared to the normalised data (black) shows disagreement model-data as MSE (background colour), suggesting that some phosphopeptides are consistent with literature, while many reveal an unknown role and require further investigation.

A

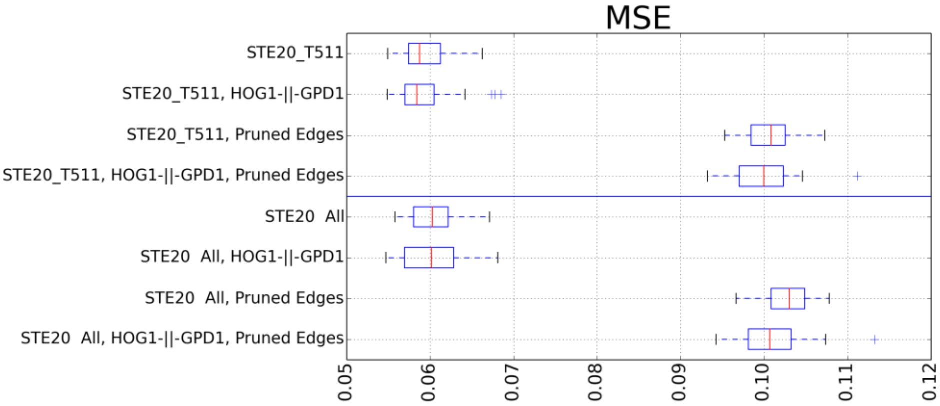
MSE
STE20_T511
STE20_T511, HOG1-||-GPD1
STE20_T511, Pruned Edges
STE20_T511, HOG1-||-GPD1, Pruned Edges
STE20 All
STE20 All, HOG1-||-GPD1
STE20 All, Pruned Edges
STE20 All, HOG1-||-GPD1, Pruned Edges
0.05
0.06
0.07
0.08
0.09
0.10
0.11
0.12

B

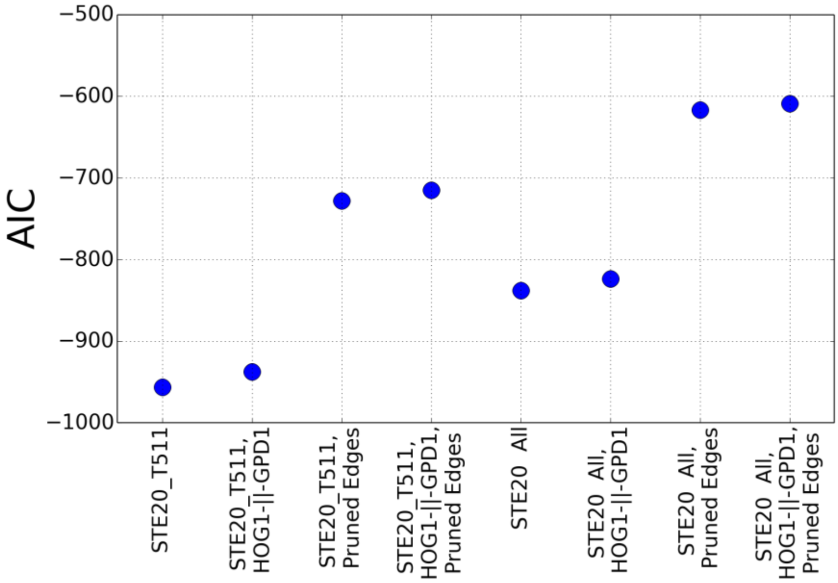
AIC
−500
−600
−700
−800
−900
−1000
STE20_T511
STE20_T511, HOG1-||-GPD1
STE20_T511, Pruned Edges
STE20_T511, HOG1-||-GPD1, Pruned Edges
STE20 All
STE20 All, HOG1-||-GPD1
STE20 All, Pruned Edges
STE20 All, HOG1-||-GPD1, Pruned Edges

C

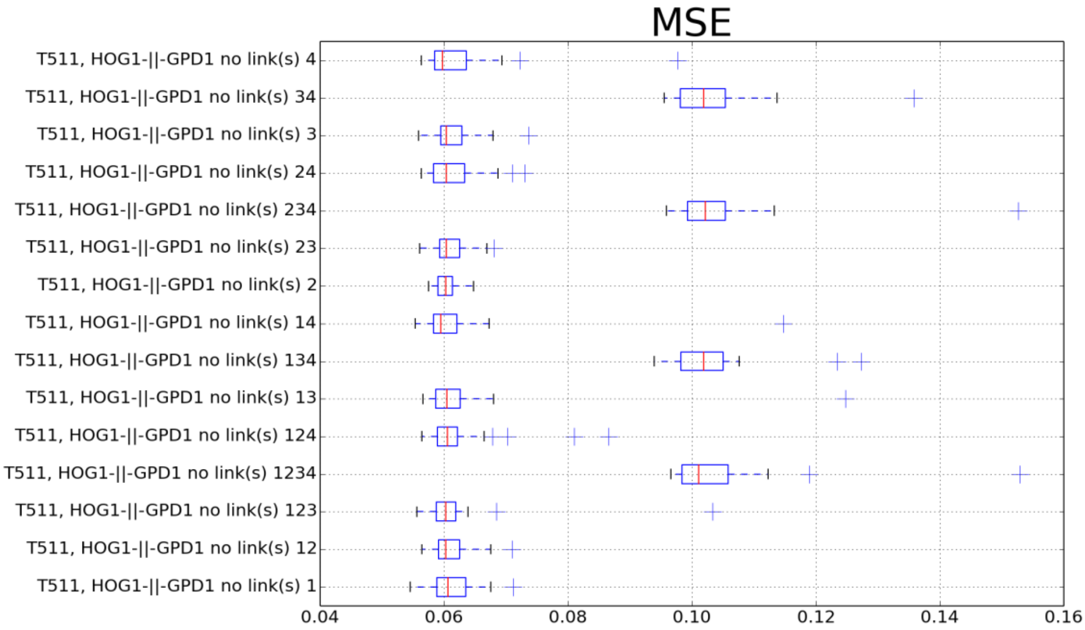
MSE
T511, HOG1-||-GPD1 no link(s) 4
T511, HOG1-||-GPD1 no link(s) 34
T511, HOG1-||-GPD1 no link(s) 3
T511, HOG1-||-GPD1 no link(s) 24
T511, HOG1-||-GPD1 no link(s) 234
T511, HOG1-||-GPD1 no link(s) 23
T511, HOG1-||-GPD1 no link(s) 2
T511, HOG1-||-GPD1 no link(s) 14
T511, HOG1-||-GPD1 no link(s) 134
T511, HOG1-||-GPD1 no link(s) 13
T511, HOG1-||-GPD1 no link(s) 124
T511, HOG1-||-GPD1 no link(s) 1234
T511, HOG1-||-GPD1 no link(s) 123
T511, HOG1-||-GPD1 no link(s) 12
T511, HOG1-||-GPD1 no link(s) 1
0.04
0.06
0.08
0.10
0.12
0.14
0.16

**D**

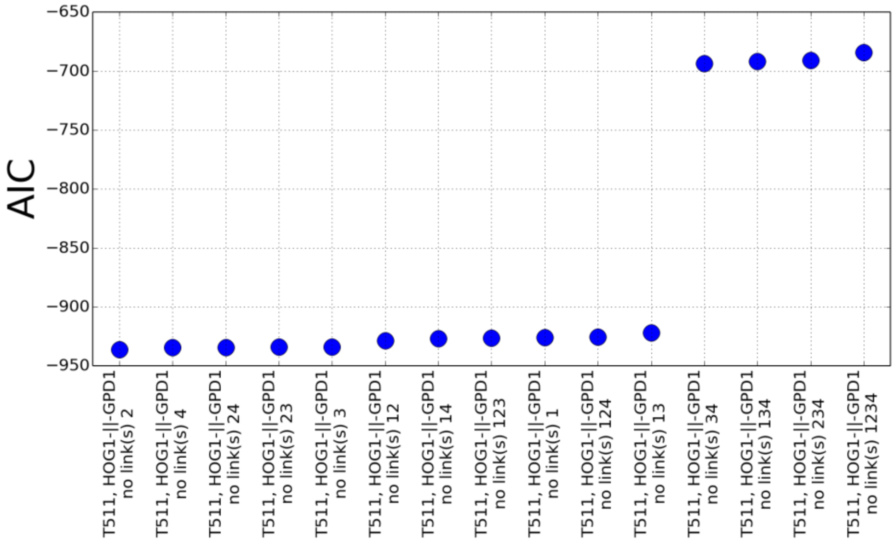


**Supplementary Figure S8** Model selection plot. Accuracy measures in supp table modelSelection are visualized here. (**A**) MSE for each model containing the proposed mechanisms, i.e. STE20 T511 as main crosstalk regulator, HOG1-GPD1 double inhibition , and p-peps with no effect on the shape of the dynamic curves shown in Figure 4 were removed (labelled pruned edges). To compare the data and the model, RMSE was computed upon 40 scatter search optimization runs during 48 hours for each model (red: median of the error, whiskers: standard deviation of the model MSE). (**B**) The AIC for each model in (A) is shown here. (**C**) The model suggested that at least one of the pruned edges was essential, hence we investigated the 15 combinations removing individually each edge and recalculate the MSE and the AIC (**D**).

| Identifier | Reason for being discarded | Number of peptides removed |
|---|---|---|
| GPD1 | Clustering of 3 peptides into 1 | 2 |
| DIG2 | Clustering 3 peptides into 1 | 2 |
| DIG1 | Clustering 7 peptides into 4 | 3 |
| FAR1 | 1 peptide with no time 0’ | 1 |
| SSK1_S110 and SSK1_S673 | Data with 6 missing values out of 16 points | 2 |
| STE20_ST413+S418 | 6 missing values out of 16 points | 1 |
| STE20_T546+S547 | 6 missing values out of 16 points | 1 |

**Supplementary Table S4 -** Phospho-peptides that were either compressed due to redundant function or removed due to data sanity check.

| |
|---|
| DIG1_S126-S127 |
| DIG1_S272 |
| DIG1_S272+T277+S279 |
| DIG1_S395 |
| DIG2_S225 |
| FUS3_T180 |
| FUS3_T180+Y182 |
| GPA1_S199 |
| GPD1_S24+S27 |
| HOG1_T174-Y176 |
| HOG1_T174+Y176 |
| HOT1_S153 |
| PBS2_S248 |
| PBS2_S269 |
| PBS2_S68 |
| PTP2_S258 |
| SKO1_S94+S108+T113 |

| |
|---|
| SKO1_T215 |
| SLN1_S833 |
| SSK1_S193+S195 |
| SSK1_S351 |
| SSK2_S53-S54+S57 |
| STE11_S323 |
| STE12_S400 |
| STE20_S192 |
| STE20_S195 |
| STE20_S196+T197 |
| STE20_T170+T172 |
| STE20_T203+T207 |
| STE20_T511 |
| STE20_T573 |
| STE2_S331 |
| STE50_S202 |

**Supplementary Table S5 -** The 33 measured phospho-peptides that were included in a model, derived from the signalling network shown in Figure 1, which also included 10 unmeasured signalling intermediates. In addition, to model the scaffolding and recruiting of STE11 and STE50, we included an “AND” node between these nodes within the network. Finally, we also included two nodes to represent salt and alpha stimulation, hence the total number of nodes in the network sums up to 46.

## Additional captions

**Supplementary Table S1 -** List of all the phospho-peptides discussed in this work, with their average normalized intensities, standard deviations, and number of non-zero biological replicates, for each stimulation condition of the Matrix (Figure 1B). For each phospho-peptide we also indicate whether they have an SP or a TP motif within their sequence, as these motifs are known to be targeted by MAP kinases such as Hog1 and Fus3.

**Supplementary Table S2 -** Dataset produced in the Mock 1' pheromone and in the Mock 1' NaCl stimulations, respectively. For each phosphorylated-peptide, we indicate the detected (or missing) normalized intensities.

**Supplementary Table S3 -** Specificity Matrices relative to all the measured phosphorylated-peptides belonging to the Hog or to the pheromone pathways.

**Supplementary Table S6** - Accuracy measures MSE and AIC for the 23 models (k: number of model parameters, n: number of data points). To determine the role of the phospho-peptides detected and the mechanisms proposed, we developed a model for each combination mechanisms. The model shown in Figure 8A shows the state of the art protein knowledge at the phospho-peptide (P-pep) level. The essentiality of the novel crosstalk mechanism shown in Figure 7K was tested by removing all Ste20 P-peps but Ste20_T511 (labelled STE20_T511). Analogously, Figure 7L was included as a double inhibition between Gpd1 and Hog1 (labelled HOG1-||-GPD1). Finally, the P-peps with no effect on the shape of the dynamic curves shown in Figure 4 were removed (labelled pruned edges). This revealed an important loss of model performance, therefore we removed the edges individually to identify that the reason for this. We found this in the exclusion of Ptp2-Hog1 regulation (links: 1: STEcomplex 1 PBS2_S269, 2: SSK2_S53-S54~S57 1: PBS2_S68, 3: HOG1_T174-Y176 1 PTP2_S258, 4: HOG1_T174^Y176 1 PTP2_S258).
