# Supplementary figures and images for: Phosphoproteomic analyses reveal novel cross-modulation mechanisms between two signaling pathways in yeast
Source: Mol Syst Biol. 2014 Dec 9;10(12):767. doi: 10.15252/msb.20145112 (PMC4300490; doi:10.15252/msb.20145112)

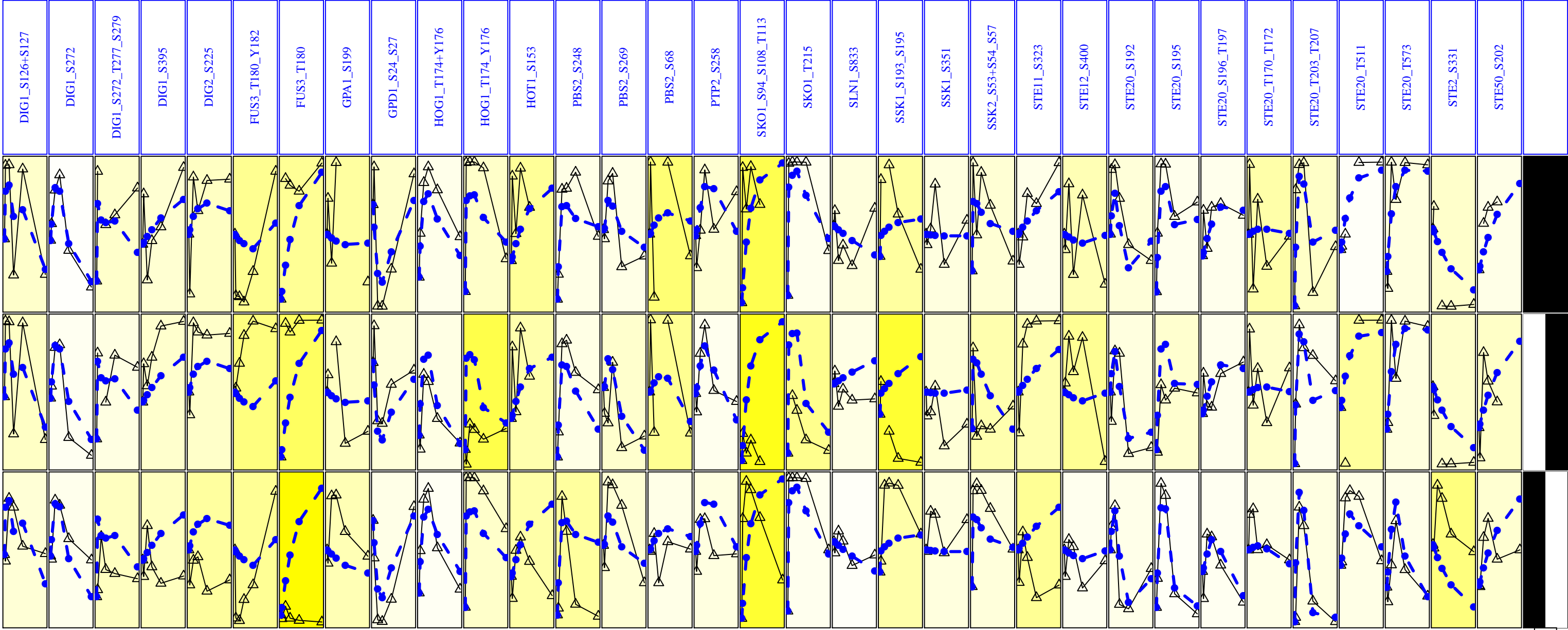

NaCl<sub>a</sub>

Error

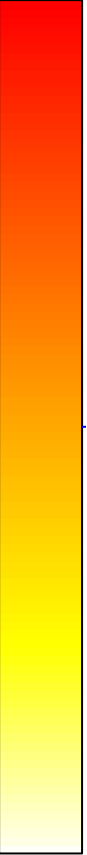

Supplement: Supplementary file 6 [file msb0010-0767-sd6.zip › Data Archive Models/STE20_All/Fitness.pdf]

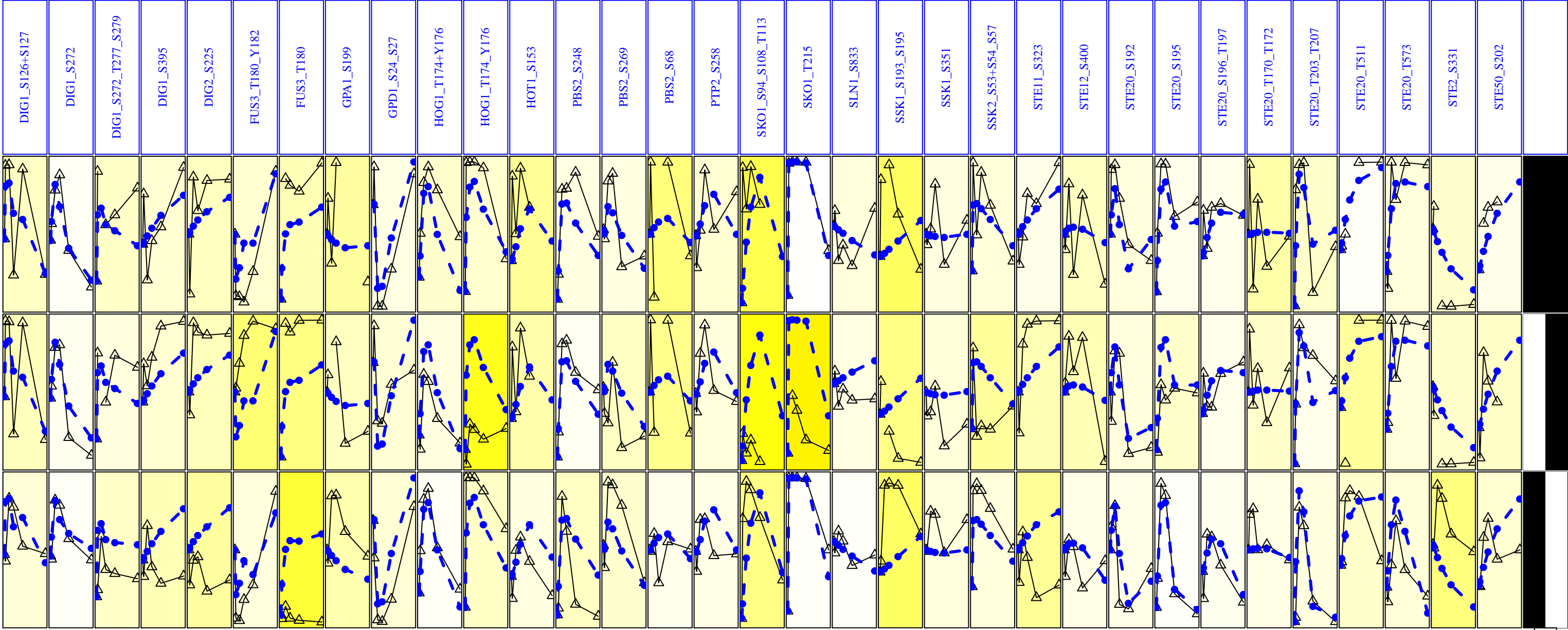

NaCl<sub>a</sub>

Error

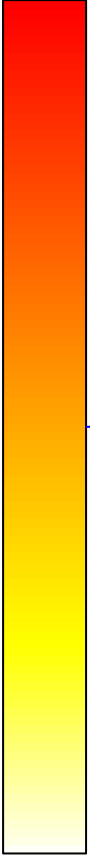

Supplement: Supplementary file 6 [file msb0010-0767-sd6.zip › Data Archive Models/STE20_All_HOG1_GPD1/Fitness.pdf]

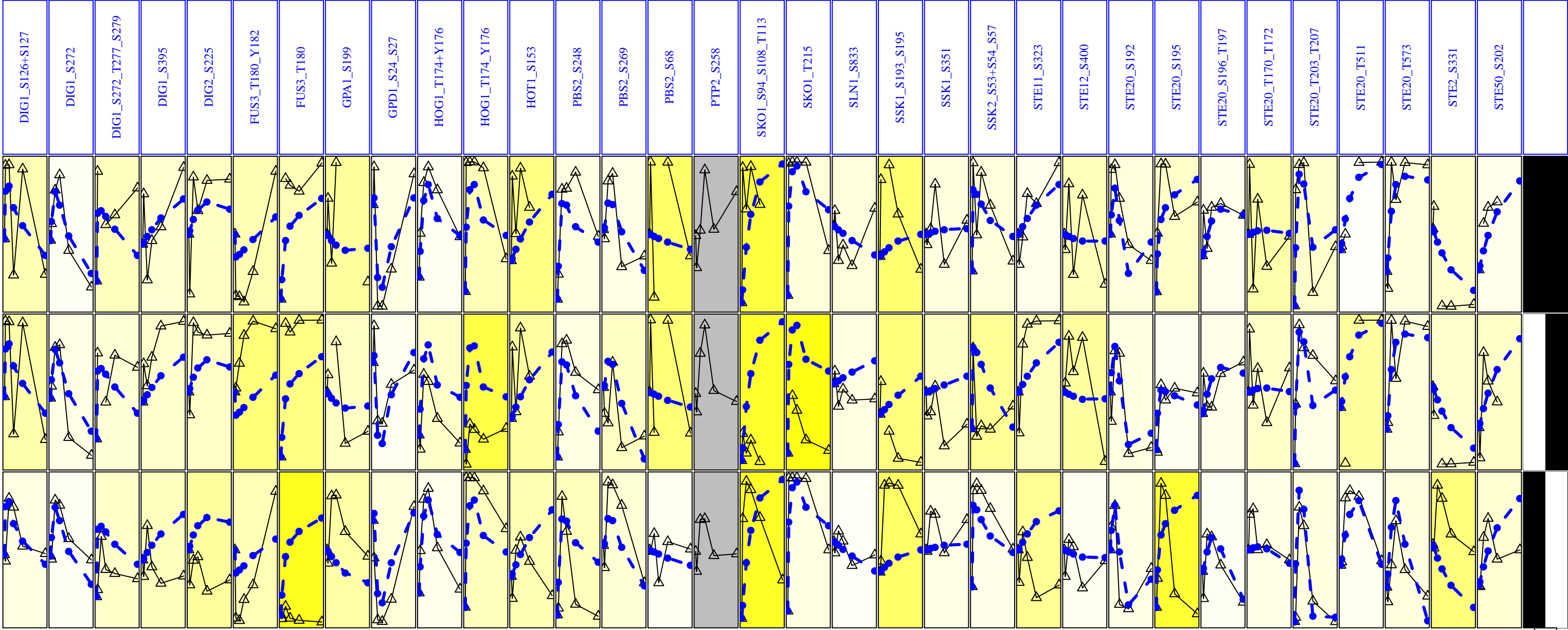

NaCl

a

Error

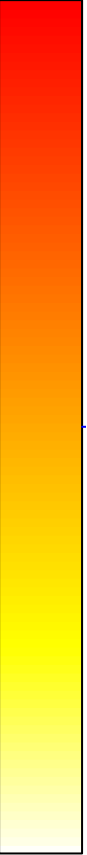

Supplement: Supplementary file 6 [file msb0010-0767-sd6.zip › Data Archive Models/STE20_All_HOG1_GPD1_pruned_edges/Fitness.pdf]

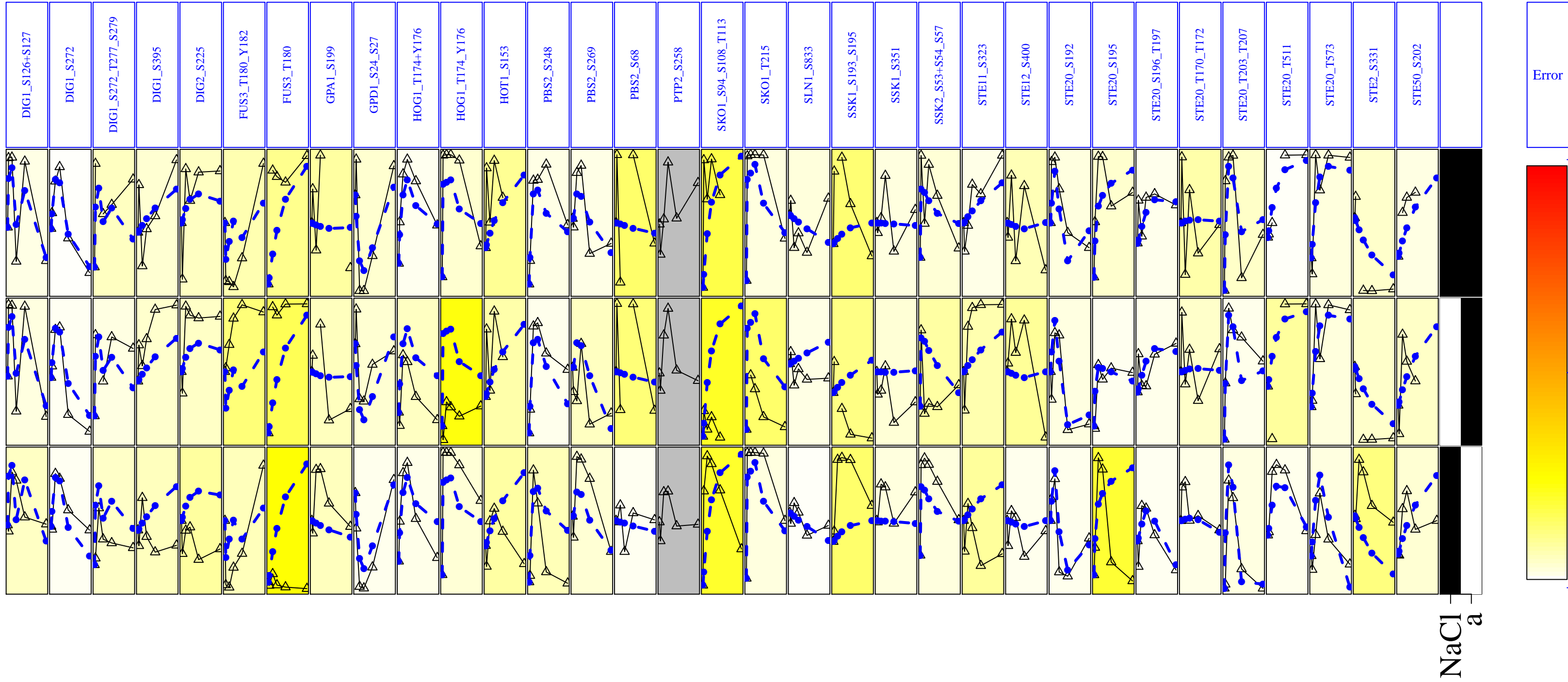
$$\text{NaCl}$$

Supplement: Supplementary file 6 [file msb0010-0767-sd6.zip › Data Archive Models/STE20_All_pruned_edges/Fitness.pdf]

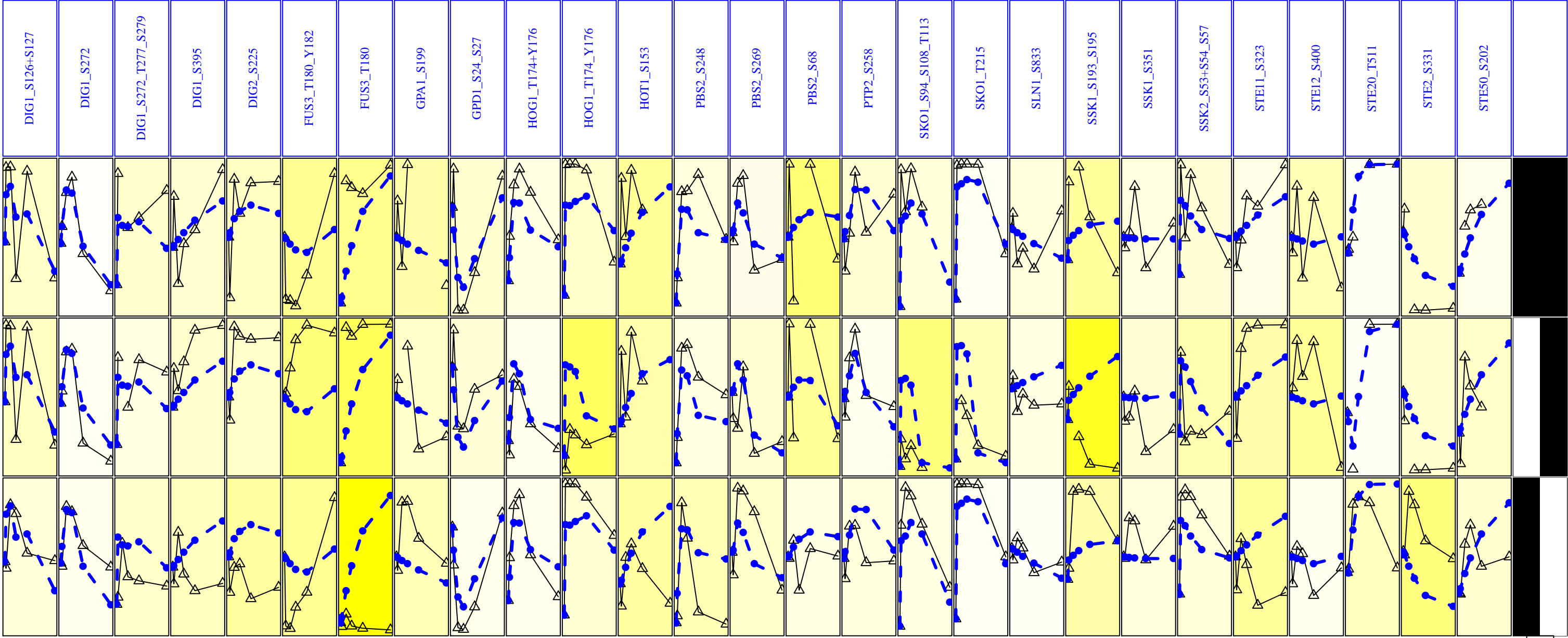

NaCl  
a

Error

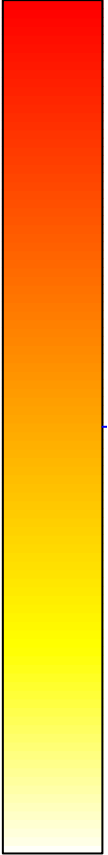

Supplement: Supplementary file 6 [file msb0010-0767-sd6.zip › Data Archive Models/STE20_T511/Fitness.pdf]

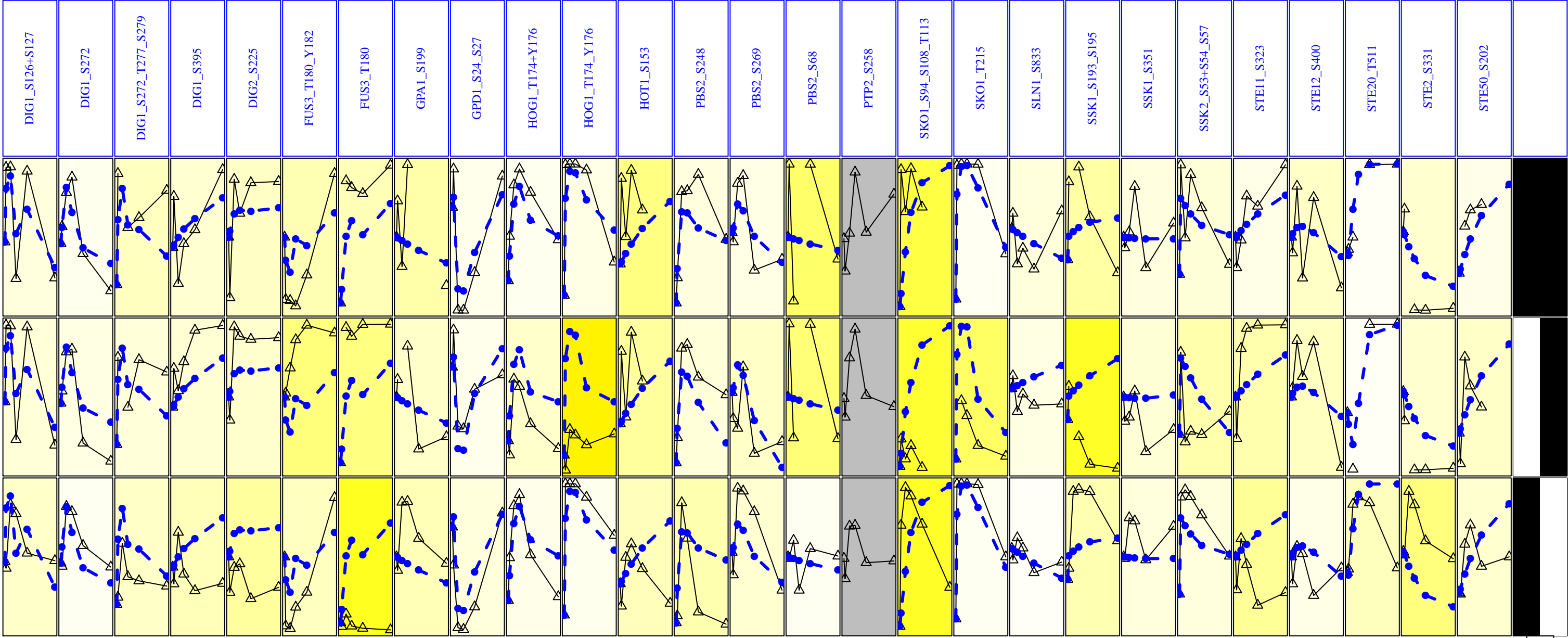

NaCl  
a

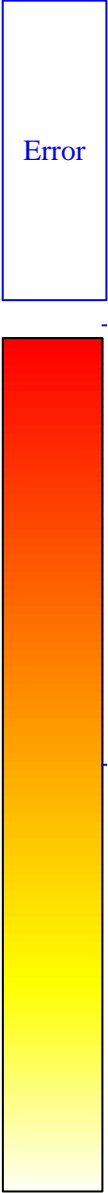

Supplement: Supplementary file 6 [file msb0010-0767-sd6.zip › Data Archive Models/STE20_T511_HOG1_GPD1_pruned_edges/Fitness.pdf]

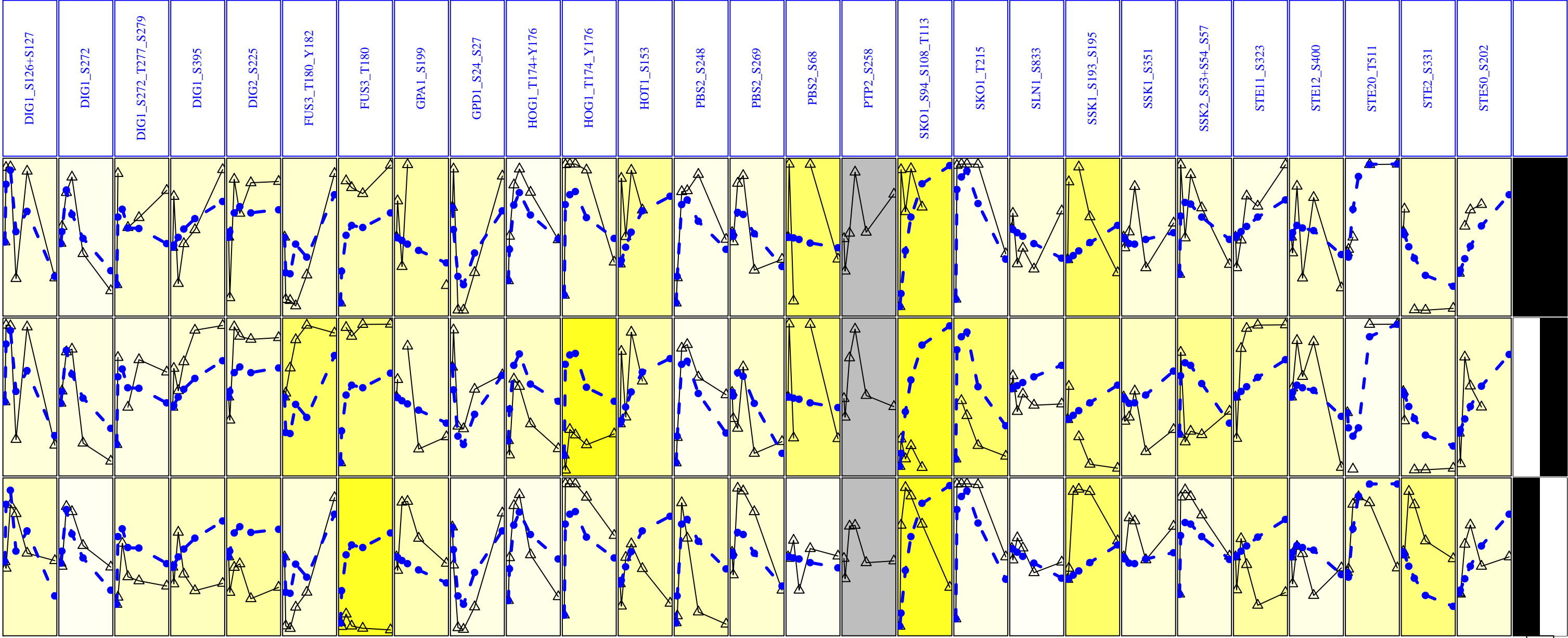

NaCl  
a

Error

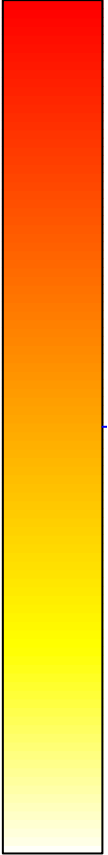

Supplement: Supplementary file 6 [file msb0010-0767-sd6.zip › Data Archive Models/STE20_T511_pruned_edges/Fitness.pdf]
