# Supplementary material for: Phosphoproteomic analyses reveal novel cross-modulation mechanisms between two signaling pathways in yeast
Source: Mol Syst Biol. 2014 Dec 9;10(12):767. doi: 10.15252/msb.20145112 (PMC4300490; doi:10.15252/msb.20145112)
Supplement: Supplementary file 7 [file msb0010-0767-sd7.pdf]

Phosphoproteomic analyses reveal novel cross-modulation mechanisms between two signaling pathways in yeast

Stefania Vaga, Marti Bernardo-Faura, Thomas Cokelaer, Alessio Maiolica, Christopher A Barnes, Ludovic C Gillet, Bjo rn Hegemann, Frank van Drogen, Hoda Sharifian, Edda Klipp, Matthias Peter, Julio Saez-Rodriguez, Ruedi Aebersold

Corresponding author: Ruedi Aebersold, ETH Zurich

Review timeline:

Submission date:	18 March 2014
Editorial Decision:	15 April 2014
Revision received:	08 August 2014
Editorial Decision:	10 September 2014
Revision received:	10 October 2014
Pre-acceptance letter:	17 October 2014
Accepted:	07 November 2014

Editor: Maria Polychronidou

Transaction Report:

(Note: With the exception of the correction of typographical or spelling errors that could be a source of ambiguity, letters and reports are not edited. The original formatting of letters and referee reports may not be reflected in this compilation.)

1st Editorial Decision 15 April 2014

Thank you again for submitting your work to Molecular Systems Biology. We have now heard back from the three referees who agreed to evaluate your manuscript. As you will see from the reports below, the referees raise a series of concerns, which should be carefully addressed in a revision of the manuscript.

Overall, the referees acknowledge the comprehensive datasets and appreciate that the reported cross-talk between the HOG and pheromone signal transduction pathways is potentially interesting. However, they point out that, in absence of follow-up investigations, the biological significance of the interactions between the two pathways remains unclear. As such, referees # 1 and #2 refer to the need to include further experimental or computational analyses, demonstrating the functional consequences of the pathway cross-talk and the observed phosphorylation dynamics. In terms of the more technical points raised by reviewer #1, we feel that the validation by Western blot might not be an absolute necessity. The issue regarding 'mock controls', especially for very early time points might be more important to address. Reviewer #1 also listed a very long list of additional issues, which are however all addressable by providing the appropriate clarifications.

If you feel you can satisfactorily deal with these points and those listed by the referees, you may wish to submit a revised version of your manuscript. Please attach a covering letter giving details of the way in which you have handled each of the points raised by the referees. A revised manuscript will be once again subject to review and you probably understand that we can give you no guarantee at this stage that the eventual outcome will be favorable.

© European Molecular Biology Organization

Reviewer #1:

The manuscript by Vaga et al present extensive data on the time course of protein phosphorylation of components of the pheromone and high osmolarity signal transduction pathways in yeast under a variety of combined pheromone and salt treatments. The data are comprehensive, although not exhaustive, encompassing most of the proteins of the two pathways. The data are also of high quality and carefully curated.

The main conclusion from this initial analysis of the data is that the two pathways exhibit extensive crosstalk, at a level much greater than anticipated by earlier experiments. Most of the proteins alter their phosphorylation state in response to both signaling pathways and co-stimulation of both pathways yield complex patterns that shift depending on the relative order and duration of the two stimuli. These results indicate a significant interplay of the two pathways, at least at the level of posttranslational modification, which may suggest a substantive biological interplay of the pathways.

What is lacking from this study is any sense of biology and causality. First, the outputs of the pathways are not measured under the various conditions tested. Accordingly, we do not know if different kinetic patterns result in different signaling outputs or are simply fluctuations without altering information flow. Second, the functional consequence of most of the phosphorylation sites is unknown, except for a few cases - such as Gpd1_S24_S27 or Hog_T174_Y176 - in which the functionality had been established from previous studies. Accordingly, whether the observed phosphorylations have biological consequences or are simply bystander modification isn't known. Third, the authors attempt to draw functional links between different components but do so only on the basis of similarities of phosphorylation kinetics. Less discussion of data patterns and more effort to establish functional connections would have been useful. Nonetheless, the data should prove useful in pointing subsequent studies to potentially informative molecular genetic experiments.

Minor points:

Treatment of cells with the Cdc28 inhibitor for only one hour would not lead to full synchronization, since the cell cycle is longer than that.

The results in Figure S1 are different than those in Figure 2B, even though the experiments are ostensibly identical. This raises some questions about the reproducibility of the experimental results.

The columns in Figure 3D need to be labeled. I assume they are in the same order as in 2C, but that is not stated. Moreover, each row should be labeled with the corresponding P-pep. The excerpted summary of the membership of the cluster is much less useful.

The statement that the members of Figure 3D cluster 2 "were affected by pheromone only 1' after pheromone stimulation" is difficult to appreciate from the figure. The statement makes sense only after one appreciates that the authors are referring to the fact that NaCl stimulates phosphorylation at all regimens of pheromone treatment except for the 1' time point. That could be explained more clearly.

Asserting that Ssk1_S110 and Pbs2_248 have the same kinetics on the basis of the data in Figure3D is not evident to the reader unless the rows for those two peptides are labeled.

In all the 2D representation of the time course of response, the data points are connected by a smoothed curve. This is an inappropriate extrapolation of the behavior of the system, since there is no way to know what the actual values are that lie in between the measured data points. Rather, the data points should simply be connected by straight lines to designate their grouping.

Reviewer #2:

Review of manuscript MSB14-5112, entitled "Phosphoproteomic analyses reveal novel cross-

modulation mechanisms between two signaling pathways in yeast" by Stefania Vaga et al.

In this manuscript, the authors tackle the general problem of cross-talk between signaling pathways. They chose the well-known case of the cross-talk between the mating pheromone response and the high osmolarity response (HOG) pathways in the yeast S.cerevisiae. These two responses use MAPK cascades that have the intriguing characteristic of sharing some of the upstream components, yet, in response to a single input (mating pheromone, for example) cells only activate/execute only the cognate response (induction of mating specific genes, for example). Thus, to a very first approximation, these pathways are isolated from each other. Several studies over the years have studied the basis for this functional isolation, employing various experimental regimes, including co-stimulation, and while some mechanisms that prevent cross-activation have been found, the overall picture is far from clear. There are even some studies that show that, under some conditions, one pathway may indeed stimulate/recruit the other. Cross-talk is a general problem, and the interaction between these two MAPK pathways serves as a prototypical case study for many such cascades operating in most eukaryotes, including humans. Thus, I think that it is of sufficient general interest for MSB.

The authors experimental approach, following their past demonstrated expertise, was to design a particular experimental setup of co-stimulation that would enable the manifestation of cross-talk, and then identify, using quantitative shot gun mass-spectroscopy, as many phosphopeptides as possible of pathway components. The idea was that they would be able to detect phosphopeptides and changes in their amounts, that would reveal previously unsuspected points of cross-talk between the pathways. Indeed, they found many phosphopeptides of proteins belonging to one pathway (including the MAPKs, Fus3 and Hog1) that change due to the activity of the other. That is, they found a much larger than previously suspected evidence of cross-talk. As I will detail below, I have some major concerns, plus numerous minor issues, that somehow prevent me from forming a complete picture of the significance of the findings.

Major concerns:
1) Experimental setup/sample collection of the data.
From reading the manuscript it seems that there were no control (mock) stimulation. That is, a given sample, such as that explained in detail in the legend to Figure 1b (20' NaCl - 5'pheromone), does not have its mock-stimulated counterpart. In this case, that would have been a 5' "stimulation" with medium without pheromone. This procedure would have controlled for culture handling effects (removing from the incubator, stimulation, placing again, removing again). To me, this is critical, especially for the 1' pheromone samples. Consider that in these particular cases, the sample is taken just 1' after pheromone stimulation. So, based on the explanation the authors give in the legend, the culture was first removed from the incubator at -20' relative to collection time), then at -19, then at 0:
"As an example, the cell cultures 3 biological replicates) relative to the square highlighted by an asterisk were stimulated as follows: NaCl was added to the cultures, which were then placed back in the incubator; 15 minutes after, pheromone was added to the same cultures, which were then placed again in the incubator; after another 5 minutes, the cells were harvested. Total duration of NaCl stimulation: 20'. Total duration of pheromone stimulation: 5'."
The importance for the overall assessment of the paper cannot be overestimated. Consider that some of the most important conclusions derive from the unsuspected behavior of key phosphopeptides in the 1'phe samples.
The analysis related to the 1'phe samples starts, if I am not mistaken, with in Fig 5b, where they show that the Hog1 activating peptide is profoundly downregulated in this 1'phe sample (independently from the time of NaCl addition). From then on, the authors try to track the origin of this downregulation.

2) Lack of validation of their results. The authors have not performed any validation of the behavior observed for their phosphopeptides using another technique. While for the vast majority of the phosphopeptides there are no commercial antibodies, for some, such as the activating phosphorylation in Hog1, there are very good ones. The authors should repeat some of the time courses, such as the critical 1'phe time-courses, and show, for example, that they can detect the strong downregultaion at 1' in Hog1 phosphorylation by Western blotting, or similar technique. In this case, the mock stimulation control should be included as well.

3) Lack of physiological/cell biological consequences demonstrated. The authors have not performed follow up experiments to assess the importance of the multiple cross-talk points they have uncovered. That is, are any of these points of cross-talk relevant for pathway performance? Or are they just a sort of "spill-over phosphorylation" that the system copes with no consequence? They should mutate selected phosphopeptides to their non-phopshorylated counterparts (for example, A/V to mimic no phosphorylation and D/E to mimic constitutive phosphorylation, for the cases of S/T, respectively). Then, in strains expressing these mutants, perform an experiment to show a change in pathway behavior (for example, speed of volume recovery for HOG, mating efficiency for the pheromone response, etc).
Without these, the overall importance of the findings remains in question.
This last critique is related to another characteristic of this work, which I would call uni-dimensionality: it consists of one single experiment of 36 samples, analyzed by a single (although powerful) technique. This is, in my opinion, an important weakness of this work.

Relatively minor critique:

Introduction
1) First paragraph. The last sentence, where it says "mechanistically investigate" cross-talk, needs more and broader references. The authors only present two references that concern the two pathways in question, which at this point have not even been introduced. At this stage in the intro, they also need more general references about mechanisms of cross-talk, etc.

2)In the second paragraph, the sentence
"while a small fraction of the active Hog1 remains in the cytoplasm and phosphorylates other" needs a reference.
Similarly, in the next paragraph the sentence "A fraction of the active Fus3 then relocates to the nucleus to affect the expression of several genes" needs a reference or rephrasing.
Is there hard evidence that a small fraction of Hog1 remains in the cytoplasm or that only a fraction of Fus3 relocates? Or is it more likely that there is constant shuttling and at any given time only a fraction is in the nucleus or the cytoplasm?

3)In the third paragraph, beginning with "The HOG and the pheromone pathways..." it says:
"Ste20 activates Ste11 in both pathways and the response specificity is achieved by means of scaffold proteins (Patterson et al., 2010), kinetic insulation (Behar et al., 2007) and protein relocation (Yamamoto et al., 2010). Further specificity is achieved by mutual inhibition (McClean et al., 2007).
As far as I know, there is no proof that scaffolds are the reason for the insulation. Certainly Patterson et al do not show that. As many before, they hypothesize that scaffolds insulate. Similarly, Behar et al do not show that kinetic insulation is at work here. Again, they suggest that such a mechanism might be at work. Finally, the experiments shown by McClean et al that lead to the notion of mutual inhibition, were utterly disproved by Patterson et al 2010.

4)In the same paragraph:
"while another set of enzymes, such as Ssk1, Sst2, Dig1, and Dig2 are known to inhibit the enzymatic activity of different components of the two pathways"
Ssk1, Dig1 and Dig2 have not, as far as I known, an associated enzymatic activity.

5)Then, in the same paragraph:
While it is well established that the hyperosmotic stress response inhibits the mating response (Westfall et al.,2008),
I think this is the incorrect reference to a paper from Thorner and colleagues. It should be Patterson et al 2010.

Results

6)First paragraph, it says:
"Accordingly, early time-points after stimulation were favored over later ones, as the activation events of the two MAPK cascades occur predominantly within 10 minutes upon stimulation Supplementary Figure S1"
Where do we see in Fig S1 that only the first 10 minutes are relevant to the pheromone response

© European Molecular Biology Organization

pathway?

7)In the same paragraph:
"Cells were first cell-cycle arrested by Cdc28 analog sensitive inhibition for 1 hour (Shokat and Velleca, 2002) to synchronize their responses to the subsequent stimulations"
Why the authors decided to inhibit Cdc28? Why they did it for one hour? To synchronize which reponse? Both the pheromone and the HOG pathways influence the cell cycle and they in turn are influenced by it. Please explain in more detail in the text the reasoning and associated references behind this experimental decision.

8) Second page, first paragraph, it says:
"To assess the reproducibility of the measured dynamic profiles and their agreement with published data, we performed NaCl-only and pheromone-only time-course experiments in duplicate."
What should we make of this statement? In the legend to Fig1b, it says that the actual co-stimulation experiment was done in triplicates. This control was only done twice? Why? Given that in Fig S1 one can appreciate variation between repetitions, it would be important for the authors to show the behavior of the three biological replicate time courses for at least some of the phosphopeptides in the co-stimulation experiment.

9)Second page, second paragraph, the authors present an experiment in which they monitor the activating peptide in Hog1 and Fus3, which they show in Fig S1. The stated purpose is to address reproducibility and agreement with published data.
As to reproducibility: What is the error bar presented? Do the authors consider that the two biological replicates are not statistically different? To me, using their definition, I see a "shape effect" between the repetitions for the Hog1 peptide. The statistical methods should be explicit. This comment applies to the rest of the manuscript as well.
As to agreement with published data, the authors do not present a conclusion. Does it resemble or not published data? What would be the papers they are using to compare?
Then, it says:
"The level of phosphorylation [of Fus3] peaked around 20' and then steadily decreased."
I cannot see this decrease in Fig S1. On the contrary, I see a steady increase.

10)In page three of Results, it says:
"The full time-course dataset of all the detected phosphopeptides relative to the HOG and pheromone pathways is available in Supplementary Table S1."
In table S1, the data corresponds to averages of the three biological replicates mentioned in the legend to Fig1b? If so, the authors should add the standard deviation of the measurements.

11)Related to the previous comment. In general, in all their figures, the data is presented as a single data point at a given time. Are these averages? Please include the standard deviation of the three biological replicates in the plots.

12)Next paragraph, it says:
"To investigate changes in phosphorylation, we represented P-pep dynamics profiles as a 3D graph (Figure 3B) and as a combination of 2D charts (Figure 3C).
If x-axis label on Fig3b is correct, then I think that those in 3c (up and down) are swapped.
In addition, I suggest change this graph into a 2D plot, maintaining the color code for intensity. In this way, the currently unobservable regions in the 3D will be observable.

13)The legends to Fig3D and Figure 3D itself are unacceptable. It is not possible to know what peptide is in each lane or what sample is in each column. Also, what similarity metric was used for the clustering? This information is not in the legend or in the methods section.

14)Regarding the "shape and intensity effects", I could not find in the manuscript (or supplement) an explanation of how the effects were classified, scored, the statistics were made, what p-values they obtained, etc. Or what happened when a peptide exhibited both effects? For the classification, shape was given priority over intensity?

15)Later on, it says:
"Surprisingly, ppHog1 underwent a Shape Effect displayed by a strong and short-lived down-

regulation 1' after pheromone stimulation (Figure 5A), before recovering its full intensity within the next 4 minutes (Figure 5B)".

16)If Figure 5B, this downregulation is labeled "intensity effect". Which is correct?

17)Regarding the phosphopeptides found, it would be important to determine which have MAPK consensus sites and which have docking sites for at least Fus3 or Hog1. Have the authors attempted to find this out?

18)The results in the manuscript suggest a rather poor coverage of phosphopeptides. I say this because many known phosphopeptides were not picked in this analysis. For example, it is well established that Ste11 is activated by phosphorylation, and it is well established that it is activated by both signals. Thus, the fact that Ste11 phopho-Ps do not increase in both conditions suggest to me poor coverage. The authors should comment on this.

19)In connection with the results shown in Fig6b, the authors should tell us what are the main results, instead of just showing them in a rather complex Figure.

Discussion

20)In page three it says:
"Fus3 phosphorylation by Ste20, Ste11, and Ste50 is triggered
Please rephrase, as far as we know, Ste7 phosphorylates Fus3.

21)In the same page, it says:
"Indeed, Patterson et al. 2010) showed that Hog1 inhibition allows for the up-regulation of ppFus3 even during 1 M sorbitol stimulation, supporting our hypothesis"
I could not find where in this reference the authors show Fus3 phosphorylation, let alone an upregulation of it after inhibition of Fus3. Is it in another paper?

22)In the section entitled: "Gpd1 and Hog1 promote their mutual inhibition"
It says:
"The primary and quickest negative feedback mechanism predicted by Schaber et al. 2012) involves the glycerol production machinery available in cells under normal conditions."

23)I think Schaber presents this feedback as the most effective, but certainly not the quickest, since its effect requires an increase in glycerol concentration, which is not that instantaneous. Thus, the hypothesis that an increase in glycerol is the reason for the very quick and transient drop in ppHog1 is very unlikely in my opinion.

24)In the next paragraph, it says:
"In its doubly-phosphorylated form, Gpd1 is incapable of catalyzing glycerol production, but it might be able to promote the Hog1 down-regulation instead."
Are the authors suggesting that Gpd1 has a role independent of its role in glycerol production? There is no evidence for this function. Why choose this particular protein for such a role? I don't follow the reasoning.

25)The authors do not provide the phophopeptide data for the Bit61_S139_S144 peptide. Given the importance it has for the authors argument, they should include it in the dataset. Same for the Ypk1 and Gpd1 peptides.

26)The authors should discuss the published evidence that Ptc1 is activated by pheromone see Malleshaiah et al, Nature 2010, which might add extra weight to their reasoning.

Concluding remarks:

27)It says:
"Since one of the first stages of the mating response to pheromone signaling is shmooing, which consists in the formation of a cellular bulge, the cell wall integrity pathway is consequently also activated (Baltanas et al., 2013) and the cytoskeleton needs to be thoroughly reorganized,"

Buehrer and Errede, 1997 first demonstrated that the CWI pathway is activated by mating pheromone, and that it happens during shmooing. Please add that reference.

Methods

28)I think the methods section needs to be expanded quite a bit to include descriptions of all methods used, and statistical treatment of the data post phosphopeptides quantification. That is, all subsequent analysis. As it is now, it only goes to any depth in connection to the protein extraction and mass-spec work.

Reviewer #3:

In this manuscript, the authors used label-free quantitative proteomics to profile the temporal changes in protein phosphorylation in yeast cell cultures co-stimulated by NaCl and pheromone over time periods ranging from 0 to 45 minutes. They quantified 2536 phosphopeptides (1015 proteins) across 36 conditions (time points/stimuli), and obtained unprecedented details of signal integration within and between these two signaling pathways. They regrouped phosphopeptides based on the shape and intensity of the temporal changes to classify patterns and facilitate the identification of substrates from specific pathways. They also used specificity metrics to quantify the extent of inhibition or amplification of NaCl and pheromone-induced effects on substrates from these two pathways. They showed that phosphopeptides reacted to the stimuli in different ways, and that phosphosites from the same protein can show strikingly different dynamics as described for Ste20, a kinase shared by the two pathways. Interestingly, this study highlighted that the TORC2 pathway modulates Hog1 activity in response to pheromone stimulation, and that phosphorylation of Gdp1 and Hog1 results in their mutual inhibition. Overall, this study is well executed and data are presented in a clear and logical manner. In my opinion, this manuscript should be published in Mol. Syst. Biol., pending minor revisions as described below.

1. On p. 8, last sentence, the authors focus exclusively on phosphopeptides that are derived from proteins associated to the HOG/pheromone pathways. Hence, a relatively small subset of the data was selected for further analysis. Table S1 actually presents data for 53 phosphosites from 20 proteins, a subset representing less than 2 % of the entire dataset. In the context of the present experiment, it would be pertinent to present a more comprehensive view of their results and discuss how many of all phosphosites quantified were found to be regulated in response to the stimuli. It would be interesting to extend the discussion to substrates other than those from the canonical HOG/pheromone pathway to uncover new biological insights.

2. On p.9 can the authors discuss how they selected dynamic profiles corresponding to regulated phosphopeptides? They indicated that they identified phosphopeptides on 82% of the proteins from both MAPK pathways. However, how many of these were affected by the treatment?

3. It is somewhat surprising that no GO terms enrichment analysis was performed to confirm cellular pathways regulated by NaCl and pheromone stimulation. This information would be a natural extension of the analysis of regulated sites (point 1 above) and would provide a more global view of the activated pathways such as morphogenesis and cytoskeleton reorganization as discussed in the conclusion section.
4. On p. 11, the definition of shape and intensity effects is not entirely intuitive and could be clarified further. Also, the authors should provide a clear rationale to evaluate the significance of phosphopeptides undergoing shape and intensity effects.

5. On p.15, the discussion on the down-regulation of Ptp2 S258 by pheromone is not entirely clear. For example, a change in phosphopeptide intensity only ~15% (10% with 1' salt) is observed for Ptp2 without salt, is this sufficient to induce the corresponding changes in Hog1 activity? There is no biochemical validation supporting this observation.
6. On p. 20, the authors discussed the mutual inhibition of Gdp1 and Hog1. The observation that ppGdp1 promotes the down regulation of ppHog1, and that activated Hog1 promotes the dephosphorylation of Gdp1 is interesting. While Ypk1 is known to phosphorylate Gdp1, there is no information available on how Gdp1 inhibits the activity of Hog1 or what phosphatase dephosphorylates Gdp1. Could the authors provide additional information on the potential

mechanism accounting for this regulation?

7. On p.20, while Gdp1 catalyses the production of glycerol in response to NaCl, the glycerol efflux receptor Fps1 is also known to be regulated following osmotic shock. A recent report indicated that upon osmotic shock Hog1 is recruited to a MAPK docking site within the N-terminal domain of Fps1 and phosphorylate a redundant pair of regulators, Rgc1 and Rgc2 to induce their eviction from the C-terminus region and closure of the Fps1 channel (Genes & development 27, 2590-2601, 2013). Can the authors discuss the changes in phosphorylation of Fps1 and Rgc1/2 in the contect of their experiments?

1st Revision - authors' response 08 August 2014

(see next page)

Editor comments:
Overall, the referees acknowledge the comprehensive datasets and appreciate that the reported cross-talk between the HOG and pheromone signal transduction pathways is potentially interesting. However, they point out that, in absence of follow-up investigations, the biological significance of the interactions between the two pathways remains unclear. As such, referees # 1 and #2 refer to the need to include further experimental or computational analyses, demonstrating the functional consequences of the pathway cross-talk and the observed phosphorylation dynamics. In terms of the more technical points raised by reviewer #1, we feel that the validation by Western blot might not be an absolute necessity. The issue regarding 'mock controls', especially for very early time points might be more important to address. Reviewer #1 also listed a very long list of additional issues, which are however all addressable by providing the appropriate clarifications.

Reviewers' comments

We thank the Editor and the Reviewers for their comments on our manuscript. In the following point-by-point responses, we have addressed their respective comments using italic font. We hope they will find that the revised manuscript more clearly explains the robustness of our measures, and the functional significance of the crosstalk we observed between the Hog and the pheromone pathways.

Reviewer #1

The manuscript by Vaga et al present extensive data on the time course of protein phosphorylation of components of the pheromone and high osmolarity signal transduction pathways in yeast under a variety of combined pheromone and salt treatments. The data are comprehensive, although not exhaustive, encompassing most of the proteins of the two pathways. The data are also of high quality and carefully curated.
The main conclusion from this initial analysis of the data is that the two pathways exhibit extensive crosstalk, at a level much greater than anticipated by earlier experiments. Most of the proteins alter their phosphorylation state in response to both signaling pathways and co-stimulation of both pathways yield complex patterns that shift depending on the relative order and duration of the two stimuli. These results indicate a significant interplay of the two pathways, at least at the level of posttranslational modification, which may suggest a substantive biological interplay of the pathways. What is lacking from this study is any sense of biology and causality.

We thank Reviewer 1 for the thorough analysis of the manuscript. In the following, we present our corrections and explanations to the specific raised issues.

1. First, the outputs of the pathways are not measured under the various conditions tested. Accordingly, we do not know if different kinetic patterns result in different signaling outputs or are simply fluctuations without altering information flow.

The aim of our work was to investigate the integration of NaCl and pheromone stimulation by two MAP kinase cascades. Our focus was on the signal integration that takes place in the very first stages of the response to the said stimuli, which does not encompass the resulting output of the integration. However, among the proven and commonly used indicators for the Hog and the pheromone pathways activation (Schüller et al., 1994; Yu et al., 2008; Muzzey et

al., 2009), there is the up-regulation of the doubly-phosphorylated MAP kinases (Hog1 and Fus3, respectively) at well-known phospho-sites (Hog1 at T174 Y176, and Fus3 at T180 Y182, respectively). It has been demonstrated that this double phosphorylation is required for the activation of the MAP kinases and for the onset of a response to the activating stimuli. The activation of the two MAP kinases can be therefore considered as the first output of the two pathways, which subsequently triggers a cascade of other responses and, as such, can be used as a measure of the response induced by the stimulation. We therefore believed it was not necessary to perform any further measurements of the cells response other than showing how Hog1 and Fus3 are being phosphorylated in each of the stimulation condition analyzed. For better clarity, we have inserted in the text one more reference that further proves how Hog1 needs to be phosphorylated at T174 and Y176 for the MAP kinase activation, relocation to the nucleus, and initiation of the pathway response (Schüller et al., EMBO J. 1994 Sep 15; 13(18): 4382-9). The already reported reference from Yu et al. already clearly proves that Fus3 phosphorylation at T180 and Y182 is required for Fus3 activation.

2. Second, the functional consequence of most of the phosphorylation sites is unknown, except for a few cases - such as Gpd1_S24_S27 or Hog_T174_Y176 - in which the functionality had been established from previous studies. Accordingly, whether the observed phosphorylations have biological consequences or are simply bystander modification isn't known.

In this study we have detected a large number of phosphorylation sites (79), within the components of the hyper-osmotic shock and of the pheromone pathways, most of which show significant changes under our tested conditions. This dataset is unprecedented, and supports a number of different lines of investigation. One of these is the line suggested by the Reviewer, i.e. to assign a function to some of the phospho-peptides showing an interesting behavior. Another line is to employ mathematical methods to investigate the dataset and extrapolating novel hypotheses, which could suggest novel lines of research. We chose this second line because the generated data supports such analyses at an unprecedented level, because this step should precede further experiments, and because the first line would exceed our resources.

Possibly, a fraction of the detected phospho-sites has no biological function. However, it is unlikely that phospho-peptides that fall, based on their measured dynamics, into clusters that contain functionally annotated phospho-peptides, are not themselves mediating any biological function. These phospho-sites are subject to a complex regulation that is not random. We therefore believe the phospho-peptides that consistently show specific patterns of behavior to be likely functional, although the determination of their biological function would exceed the purpose of the present paper.

3. Third, the authors attempt to draw functional links between different components but do so only on the basis of similarities of phosphorylation kinetics. Less discussion of data patterns and more effort to establish functional connections would have been useful.
Nonetheless, the data should prove useful in pointing subsequent studies to potentially informative molecular genetic experiments.

The purpose of the present work was indeed to identify patterns of behaviors that could be followed up in subsequent studies. The patterns observed in this study are quite novel because they resulted from a complex and unprecedented matrix of co-stimulation by NaCl and pheromone, and are thus considerably rich in contents. We therefore employed this complex dataset to generate novel hypotheses, which will prompt novel future lines of investigations.

Minor points:

4. Treatment of cells with the Cdc28 inhibitor for only one hour would not lead to full synchronization, since the cell cycle is longer than that.

It has been shown by Strickfaden et al (Cell 2007 Feb 9; 128(3): 519-31) that Cdc28 activity inhibits pheromone response and that, by preventing such inhibition, the pheromone pathway activates even outside G1. The aim of our Cdc28 inhibition was therefore to make cells immediately responsive to pheromone without synchronizing cell cycles.

To better explain our procedure and the reasons behind it, we have inserted the reference mentioned above in the manuscript. We have also reformulated the sentence where we describe why we use Cdc28 inhibition as follows:

"While budding yeast cells are immediately responsive to osmotic shock, this is not usually the case for pheromone stimulation. To also make cells immediately responsive to pheromone stimulation a 1 hour long Cdc28 analog sensitive inhibition (Shokat and Velleca, 2002) was employed (Strickfaden, 2007)."

5. The results in Figure S1 are different than those in Figure 2B, even though the experiments are ostensibly identical. This raises some questions about the reproducibility of the experimental results.

We thank the reviewer for this comment as it allowed us to detect a mistake in Supplementary Figure S1. As correctly observed, the shape of Fus3_T180_Y182 dynamic in Figure S1 differs from the one in Figure 2B. By reproducing the curves from the original dataset we realized that the results reported in Supplementary Figure S1 is not relative to Fus3_T180_Y182 but to Fus3_(T180)_(Y182), which is the singly phosphorylated form of the MAP kinase instead of the doubly phosphorylated one. We apologize for this mistake and we have corrected it.

Supplementary Figure S1 has been fixed. In the paragraph "Data validation", we also further commented on the behavior of Hog1_T174_Y176 and Fus3_T180_Y182 in the two experiments, highlighting both reproducibility and inter-experiment differences:

"While the shapes of the curves relative to the two experiments slightly differ in their shape (there is a secondary mild up-regulation of doubly phosphorylated Hog1), the main spikes are very reproducible, both in shape and intensity."

"Furthermore, whereas Fus3 activation curves in the two experiments have similar shapes, their overall intensities differ. This difference may be due to a dissimilar starting amount of either Fus3 or of the activating kinases upstream to Fus3 (such as Ste7)."

6. The columns in Figure 3D need to be labeled. I assume they are in the same order as in 2C, but that is not stated. Moreover, each row should be labeled with the corresponding P-pep. The excerpted summary of the membership of the cluster is much less useful.

We have labelled both rows and columns of Figure 3D.

7. The statement that the members of Figure 3D cluster 2 "were affected by pheromone only 1' after pheromone stimulation" is difficult to appreciate from the figure. The statement makes sense only

after one appreciates that the authors are referring to the fact that NaCl stimulates phosphorylation at all regimens of pheromone treatment except for the 1' time point. That could be explained more clearly.

In order to facilitate the interpretation of Figure 3D, we have inserted the information regarding the stimulation times for both stimuli on the x-axis on top of the clustering heatmap. We have also reformulated the sentence as follows:

"Cluster 2 contains the HOG pathway's P-peps that show cross stimulation dynamics similar to Hog1_T174_Y176 (ppHog1). All these P-peps appear to be affected by pheromone only 1' after pheromone stimulation."

8. Asserting that Ssk1_S110 and Pbs2_248 have the same kinetics on the basis of the data in Figure3D is not evident to the reader unless the rows for those two peptides are labeled.

We have labelled all the rows in Figure 3D with the relative phospho-peptide names.

9. In all the 2D representation of the time course of response, the data points are connected by a smoothed curve. This is an inappropriate extrapolation of the behavior of the system, since there is no way to know what the actual values are that lie in between the measured data points. Rather, the data points should simply be connected by straight lines to designate their grouping.

While the smoothing is certainly an extrapolation, it does not change the shape of the curves were they to be simply generated by linking the measured points. We thus believe that the smoothing does not affect the observations we derived from these images. We would therefore prefer not to perform this change.

We thank Reviewer 2 for the constructive and detailed analysis of the manuscript. Here we explain our positions and introduce corrections following the suggestions below.

Reviewer #2

Review of manuscript MSB14-5112, entitled "Phosphoproteomic analyses reveal novel cross-modulation mechanisms between two signaling pathways in yeast" by Stefania Vaga et al. In this manuscript, the authors tackle the general problem of cross-talk between signaling pathways. They chose the well-known case of the cross-talk between the mating pheromone response and the high osmolarity response (HOG) pathways in the yeast S.cerevisiae. These two responses use MAPK cascades that have the intriguing characteristic of sharing some of the upstream components, yet, in response to a single input (mating pheromone, for example) cells only activate/execute only the cognate response (induction of mating specific genes, for example). Thus, to a very first approximation, these pathways are isolated from each other. Several studies over the years have studied the basis for this functional isolation, employing various experimental regimes, including co-stimulation, and while some mechanisms that prevent cross-activation have been found, the overall picture is far from clear. There are even some studies that show that, under some conditions, one pathway may indeed stimulate/recruit the other. Cross-talk is a general problem, and the interaction between these two MAPK pathways serves as a prototypical case study for many such cascades operating in most eukaryotes, including humans. Thus, I think that it is of sufficient general interest for MSB.

The authors experimental approach, following their past demonstrated expertise, was to design a particular experimental setup of co-stimulation that would enable the manifestation of cross-talk, and then identify, using quantitative shot gun mass-spectroscopy, as many phosphopeptides as possible of pathway components. The idea was that they would be able to detect phosphopeptides and changes in their amounts, that would reveal previously unsuspected points of cross-talk between the pathways. Indeed, they found many phosphopeptides of proteins belonging to one pathway (including the MAPKs, Fus3 and Hog1) that change due to the activity of the other. That is, they found a much larger than previously suspected evidence of cross-talk. As I will detail below, I have some major concerns, plus numerous minor issues, that somehow prevent me from forming a complete picture of the significance of the findings.

Major concerns:

1. Experimental setup/sample collection of the data.
From reading the manuscript it seems that there were no control (mock) stimulation. That is, a given sample, such as that explained in detail in the legend to Figure 1b (20' NaCl - 5'pheromone), does not have its mock-stimulated counterpart. In this case, that would have been a 5' "stimulation" with medium without pheromone. This procedure would have controlled for culture handling effects (removing from the incubator, stimulation, placing again, removing again). To me, this is critical, especially for the 1' pheromone samples. Consider that in these particular cases, the sample is taken just 1' after pheromone stimulation. So, based on the explanation the authors give in the legend, the culture was first removed from the incubator at -20' relative to collection time), then at -19, then at 0:
"As an example, the cell cultures 3 biological replicates) relative to the square highlighted by an asterisk were stimulated as follows: NaCl was added to the cultures, which were then placed back in the incubator; 15 minutes after, pheromone was added to the same cultures, which were then placed again in the incubator; after another 5 minutes, the cells were harvested. Total duration of NaCl stimulation: 20'. Total duration of pheromone stimulation: 5'."
The importance for the overall assessment of the paper cannot be overestimated. Consider that some of the most important conclusions derive from the unsuspected behavior of key phosphopeptides in the 1'phe samples.

The analysis related to the 1'phe samples starts, if I am not mistaken, with in Fig 5b, where they show that the Hog1 activating peptide is profoundly downregulated in this 1'phe sample (independently from the time of NaCl addition). From then on, the authors try to track the origin of this downregulation.

We have performed the mock experiments for row 2 and column 2 of the Matrix, which correspond to the 1 minute pheromone and to the 1 minute salt stimulation time-course respectively. We have chosen these two time-courses since, as observed by Reviewer 2, the main conclusions we draw in our manuscript are based on the measurements relative to these two time-courses because the most striking responses, as it can be expected, happen very close to the stimulation itself (within the first 5'). Another reason for choosing these particular time-courses is that culture stimulation was, especially in the case of these time-courses, temporally much closer to the harvesting, thus possibly inducing further stress responses to the cells that would overlap with their response to the mere stimulation. The HOG pathway, in particular, may respond to other sources of stresses as well as to osmotic shock. The main questions we addressed with these mock experiments are therefore the following:

1. *Are the dynamics we measured a response to the NaCl/pheromone stimulation or are they caused by the stress induced by culture handling?*
2. *Is the response of Hog1_T174_Y176, in particular, a consequence of the stimulations? Or is Hog1's measured activation being affected by stress?*

The experiment that we named Mock_1'_Phe_time-course reproduces the time-course corresponding to the second row of the Matrix: the one where cell cultures were stimulated for 1' by pheromone before harvesting. In the mock experiment, we mock-stimulated the cells with 50 µl DMSO instead of 50 µl of DMSO dissolved α-factor. The experiment that we call Mock_1'_NaCl_time-course reproduces the time-course corresponding to the second column of the Matrix: the one where cell cultures were stimulated for 1' by NaCl before harvesting. In the mock experiment, we mock-stimulated the cells with 5.6 ml salt-free SD-medium instead of 4M SD-medium. We compared the mock_1'_Phe_time-course with the second row of the matrix, and mock_1'_NaCl_time-course with the second column. We considered the measured intensity of the matrix experiment as significantly different if it was at least 1.5 times superior/inferior to the intensity measured in the corresponding mock experiment. The choice of this threshold was manually curated: we visually decided which curves were more strongly differing from each other.

All the results of the Mock experiments are reported in Supplementary Table S2. Hog1's results are also represented in Supplementary Figures S3.

We have added a few paragraphs to the Results. To the section "A classification of NaCl or pheromone induced effects on dynamic P-pep patterns":

"As most of the Shape Effects and, to a certain extent, also the Intensity Effects, occurred in the earliest time points (within 5'), we wondered if these might be due to the culture handling which, in the case of very early time points, was temporally very close to the culture harvesting. We therefore performed two mock time-course experiments. The first was relative to row 2 of the Matrix: an equivalent volume PBS, instead of pheromone, was administered to the cultures 1 minute before harvesting (mock_1'_Phe_time-course). The second was relative to column 2 of the matrix (mock_1'_NaCl time-course) where, instead of 4M SD-medium, an equivalent volume of NaCl-free SD-medium was administered to the cells 1 minute before harvesting. We then compared the normalized intensities measured in the mock_1'_Phe_time-course experiment to those of the first row of the Matrix, and the intensities of mock_1'_NaCl_time-course to the first column of the Matrix. We considered the difference between the matrix time-course data and its corresponding mock experiment to be negligible if one value was less than 1.5 times higher/smaller than the other one. Within the HOG and pheromone pathways components, 83% of the detected P-peps did not exhibit a

significant differences between the 0’ Pheromone Matrix time-course and the mock_1’_Phe time-course, and 89% did not exhibit a significant differences between the 0’ NaCl Matrix time-course and the mock_1’_NaCl time-course. We can therefore conclude that the detected dynamics are generated by the stimulation rather than by culture handling. All the results of the mock experiments are reported in Supplementary Table S2.”

We added to the section “Stimuli crosstalk is causing Hog1 and Fus3 P-peps down-regulation”:

“ppHog1 maximum intensity was not reduced when cells were harvested 1 minute after mock pheromone stimulation (Supplementary Figure S3). The intensity reached by ppHog1 in the mock_1’_Phe time-course was, indeed, comparable to those measured for all the time-courses of our matrix experiments except the one relative to 1’ pheromone stimulation. When comparing the dynamics of the mock_1’_Phe_time-course to that of the first row of our Matrix, where no pheromone stimulation was applied, we observed similar curves both reaching comparable intensities (Supplementary Figure S3). These results suggest that the down-regulation of ppHog1 observed 1’ after pheromone stimulation is due to the stimulation itself rather than to a stress response induced by culture handling.”

2. Lack of validation of their results. The authors have not performed any validation of the behavior observed for their phosphopeptides using another technique. While for the vast majority of the phosphopeptides there are no commercial antibodies, for some, such as the activating phosphorylation in Hog1, there are very good ones. The authors should repeat some of the time courses, such as the critical 1'phe time-courses, and show, for example, that they can detect the strong downregulation at 1' in Hog1 phosphorylation by Western blotting, or similar technique. In this case, the mock stimulation control should be included as well.

To our best knowledge, there are no antibodies available for (most of) the HOG and pheromone pathways components or, especially, for their phosphorylated forms, except of Hog1(-PP) and Fus3(-PP). This is different in mammalian cells, where antibodies for Mek, Raf etc. allow at least for a semi-quantitative measurement of internal pathway activity via Western Blots. Besides the lack of antibodies for the majority of the measured phosphosites, we have observed that some of the available antibodies are not very specific. In particular, the anti-phospho-Hog1 antibody unspecifically enriches for both the singly and the doubly phosphorylated form of the protein, which have two different functional meanings, while shotgun mass spectrometry can clearly distinguish between these two forms. We would therefore prefer to avoid using phospho-antibodies to try to validate our results. Furthermore, we believe that the reproducibility of the results and the high specificity of identification of peptides by mass spectrometry obviate the need for the requested data.

3. Lack of physiological/cell biological consequences demonstrated. The authors have not performed follow up experiments to assess the importance of the multiple cross-talk points they have uncovered. That is, are any of these points of cross-talk relevant for pathway performance? Or are they just a sort of "spill-over phosphorylation" that the system copes with no consequence? They should mutate selected phosphopeptides to their non-phopshorylated counterparts (for example, A/V to mimic no phosphorylation and D/E to mimic constitutive phosphorylation, for the cases of S/T, respectively). Then, in strains expressing these mutants, perform an experiment to show a change in pathway behavior (for example, speed of volume recovery for HOG, mating efficiency for the pheromone response, etc).
Without these, the overall importance of the findings remains in question.

This last critique is related to another characteristic of this work, which I would call uni-dimensionality: it consists of one single experiment of 36 samples, analyzed by a single (although powerful) technique. This is, in my opinion, an important weakness of this work.

The reviewer raises an important point, that is, how essential the uncovered crosstalk mechanisms are for signaling. In the present study, we have collected a dataset that is unprecedented both for complexity and size. We have therefore set out to analyze it with the aim of identifying novel characteristics of the signaling integration machinery comprising the Hog and the pheromone pathways. By means of simple mathematical tools we have formulated several novel hypotheses on how the crosstalk and the integration of two separate signals take place. We have chosen not to perform any wet lab validation of these hypotheses as we believed that this would go beyond the purposes of this work and because it would have forced us to select, without clear scientific basis for this selection, one or maximally two of our novel findings. We nonetheless agree with the Reviewer regarding her/his concern that the functional consequences of the proposed mechanisms and their relation to the observed crosstalk remain unclear.

We have therefore decided to employ a mathematical modeling strategy instead. We have developed a set of logic-ODE models where we have integrated the available prior knowledge at the protein level of the Hog and pheromone pathways with the mechanisms we proposed in the manuscript at the phospho-peptide level. These models allowed us to study the observed crosstalk and the signals integration taking place within and among the two pathways. By challenging models with different mechanisms, and with the inclusion of different phosphorylation sites to explain the data, we could shed some light on which sites have functional relevance or are "spill-over phosphorylations".

The main findings of our modeling strategies were the following:

1. *Our data are in agreement with previous knowledge: the model built only on previous knowledge can reproduce the dynamics we have measured*
2. *Ste20_T511 was shown to be a key phospho-peptide, within all other Ste20 phospho-peptides, for the crosstalk between the two pathways*
3. *The mutual inhibition between Hog1_T174_Y176 and Gpd1_S24_S27 has also been shown to better explain signaling integration than previous knowledge allowed, although the simultaneous co-stimulation by NaCl and pheromone appears to partially reduce its effect.*
4. *The lack of response of certain phosphopeptides when challenged by NaCl, as described in Figure 4, was reproduced also by our models, except in the case of Ptp2, whose role in down-regulating Hog1 activity had already been shown. We therefore believe that the only phospho-site of Ptp2 that we could measure is not the one regulating Hog1's activity.*

We have therefore extended the manuscript, and we have added new figures and tables as described in the following.

We have added the following sentence to the Abstract:

"A set of logic models was then used to assess the role of measured phosphopeptides in the crosstalk."

We have added the following sentences to the Introduction:

"To put these multiple observations into a common framework, we developed a set of 23 logic models where each measured phosphopeptide (P-pep) was simulated based on the available prior knowledge of the respective phosphoprotein, and the MS measurements. The

model aided us in elucidation of the P-peps involved in the crosstalk between the HOG and the pheromone pathways, and assessed the importance for the new mechanisms proposed here."

We have added the following paragraph to the Results section:

"Mathematical modeling of the newly reported mechanisms captures signals integration dynamics

We next set out to investigate how the above described P-peps and crosstalk mechanisms are integrated in the global context of the HOG and pheromone pathways. We thus addressed the question whether the dynamic measures of the P-peps we detected are consistent both with the signaling network known to regulate the response to NaCl and pheromone (Figure 1A) and with the mechanisms proposed here.

We built a dynamic mathematical model of the pathways and variations thereof to include the mechanisms proposed above. We built our model as a set of Ordinary Differential Equations (ODEs). Because our mechanistic understanding of the P-sites measured in this study is very limited, we used a logic-based model rather than one based on the underlying biochemical reactions. The resulting model and its variations were trained to the P-pep intensities confidently detected here (Figure 2C, and Supplementary Table S1), and evaluated in terms of how well they explain the data summarized by its mean squared error (MSE). To test whether a more complex model fits the data better simply because of the higher number of parameters, we then computed the Akaike Information Criteria (AIC), which takes into account the performance of the model while penalizing the number of parameters (Burnham and Anderson, 2002).

The application of logic-based modeling to an MS dataset posed a number of challenges. Specifically, these are related to (i) the complexity of the dataset, (ii) the representation of peptides with unknown biological function, and (iii) the need to develop a model at the P-pep level instead of the more established protein level. To build the model, we first selected the P-peps with the most consistent behavior. We therefore computed the coefficient of variation of each P-pep across experiments, and we selected those with a coefficient below 25%. Subsequently, the P-peps for which 25% or more of the data points had not been detected were discarded. Next, we merged P-peps with very similar trajectories, which may have been wrongly resolved or have a redundant biological function, as they are indistinguishable to the purpose of modeling. Affinity propagation clustering revealed that a small number of P-peps indeed behaved similarly. In such cases, the P-pep with previously known function was selected. If a cluster of similar P-peps consisted exclusively of previously unknown members, a cluster representative was chosen. This filtering process rendered a final dataset of 33 P-peps. The full list of P-peps and the filtering criteria are summarized in Supplementary Table S4. Subsequently, the proteins of the starting signaling network (Figure 1A) were replaced by these P-peps. If two interacting proteins were replaced by multiple P-peps each, all the possible combinations of interactions were therefore implemented. We thus obtained a 'state of the art' logic model of 45 nodes, i.e. 33 measured P-peps (mostly with unknown function) and 12 proteins which could not be detected in a high enough number of time points, and 93 interactions (Supplementary Figure S7, A and B).

To test some of the novel mechanisms described above, we then developed a set of modified versions of our logic model either by implementing Ste20_T511 as the main Ste20 P-pep mediating crosstalk (Figure 7E), and/or by introducing the double negative inhibition between ppGpd1 and ppHog1. The model including both proposed mechanisms consisted of 39 nodes and 73 interactions (Figure 8A). Since a common formalism to build dynamic models are ODEs (Kholodenko et al., 2010), we next transformed our logic models into logic-ODEs (Wittmann et al., 2009) by means of CellNOpt (Terfve et al., 2012). We then trained all the resulting models, within CellNOpt, to the three time-course data corresponding to the

stimulation with NaCl only, pheromone only, and both stimuli at the same time, i.e. the first row, the first column, and the diagonal of the stimulation Matrix (Figure 1B), respectively.

Our results show that the model, extended with our proposed mechanisms, correctly captures the dynamics of the signal integration within the P-pep network (Figure 8B), and performs better than the one shown in Supplementary Figure S7. Specifically, with respect to the state of the art model (MSE=0.06, AIC=-837), by reducing the crosstalk mediators to only Ste20_T511 we observed no fitness loss (MSE=0.059) and a large improvement in AIC (AIC=-955). This result suggests that Ste20_T511 indeed mediates the crosstalk, while all the other P-peps of Ste20 are non-essential crosstalk mediators under these stimulatory conditions. The further addition of the ppGpd1-ppHog1 reciprocal inhibition mechanism also showed no significant increase of fitness (MSE=0.059) and, accordingly, a slight decrease in AIC (AIC=-937) due to the extra complexity. This suggests that this feedback loop might be enhancing the signaling integration at the data points which were excluded the models training (i.e. co-stimulation by both NaCl and pheromone, but not simultaneous).

Finally, we assessed whether certain co-stimulated P-peps do not affect the shape of the dynamic curves. We therefore generated models where the interactions labeled as "No Effect" upon a specific stimulation (Figure 4C), if present in the stimulated pathway according to literature, were removed. This amounts to removing from the model shown in Figure 8A the interactions that have No Effect upon NaCl stimulation, namely the interactions between Sho1 and Ste20_T573, Ssk2_S53_S57 and Pbs2_S68, the Ste11_Ste50_complex and Pbs2_S269, the singly and doubly phosphorylated forms of Hog1 and Ptp2_S258. We tested the effect of removing these 4 interactions in all possible combinations, with the model shown in Figure 8A, by developing 4 additional models. Compared to the model shown in Figure 8A, the model exhibited a loss of accuracy (MSE=0.099, AIC=-714), indicating that at least one of the removed interactions indeed played an important role in the network. We therefore generated a final set of 15 models by removing all single interactions, one at a time. We observed that in all the models with a loss of performance the interactions between Hog1 and Ptp2 had been removed. This indicates that, as previously known, the regulation of Hog1's phosphorylation by the phosphatase Ptp2 is essential also during NaCl stimulation. We therefore suggest that, according to our data, the interaction between Hog1 and Ptp2 is not mediated by the phosphorylation of Ptp2 at Ser258.

Altogether, we investigated an ensemble of 23 different models with all possible combinations of our proposed mechanisms. The ability of the model represented in Figure 8A to reproduce the data trend for most of the measured P-peps (Figure 8B) suggests that the signals propagation and crosstalk, upon NaCl and pheromone stimulation, is indeed mediated by an important number of P-peps. The performance of the full ensemble of models is summarized in Supplementary Figure S8 and in Supplementary Table S5."

We have added the following sentence to the Discussion:

"Based on this finding, we were therefore able to generate new hypotheses for how pathway component interactions allow faithful signal transmission and integration, which were supported by a mathematical model of the underlying pathways."

We have added the following paragraph to the Materials and Methods section:

*"**Phospho-peptide selection, data integration and logic modelling***

MS-DAS (https://pypi.python.org/pypi/msdas) was used to process the MS dataset and enable logic-based modelling with CellNOpt. For each P-pep, we used only the measurements acquired upon stimulation with NaCl, pheromone and both stimuli at the same time, for a total of 16 experiments out of the 36 (Figure 1B). Next, we calculated the coefficient of variation across replicates for each P-pep, selecting only those below a 0.25 threshold. P-peps for which 25% or more of the data points were missing were discarded. In the dataset used

for modelling, two single data points, i.e. FUS3_T180_Y182 and HOG1_T174_Y176 where both pheromone and NaCl were absent, were interpolated using a cubic spline as initial conditions are necessary for modelling. All P-peps belonging to the same protein were clustered to identify redundant trajectories using affinity propagation via the scikit python tool (http://scikit-learn.org/stable/), as described in the main text. To enable modeling using CellNOpt, the data was saved in MIDAS format (Saez-Rodriguez et al., 2008). Finally, proteins in the logic model corresponding to Figure 1A were replaced by the P-peps that passed the filtering process.

For logic modelling, data was normalized between 0 and 1. We used a non-linear normalization via a Hill function with a Hill coefficient of 4. The IC50 coefficient of the Hill function was determined by selecting the middle point of the cumulative distribution function using all data points for each P-pep. This normalization prevents very large values from biasing the model. Finally, each model described in the text was fit to the normalized data using the logic ODE formalism of CellNOpt embedded in the CNORode R package available in bioconductor. As a global optimization procedure, a scatter search algorithm was used, included in the R meigor package (Egea et al., 2014). Each optimization problem was run for 48 hours 50 times. Most cases converged on a very similar fit (Supplementary Figure S8).

For model selection, the AIC criterion (Burnham and Anderson, 2002), a measure where higher values indicate increased information loss, was computed using the MSE as accuracy signature. To enable comparison of models where selected P-peps were removed, the standard MSE computed by CNORode (Terfve et al., 2012) was corrected to be calculated only in the performance of the nodes present in all models. In order to account for model fit and number of data points while penalizing an increase in the number of parameters, the AIC was defined as shown in the following equation:

$$AIC = n * \log(MSE) + 2k$$

where k is the number of parameters and n the number of data points.

The models including the state of the art and the mechanisms crosstalk, ppGpd1-ppHog1 regulation and "No Effect", as well as the phospho-peptide measurements selected and normalised as MIDAS are provided in supplementary for modelling using the CellNOptR and CNOrode R packages."

Modeling results are represented in an additional Figure 8, as well as in Supplementary Figures S7 and S8, and in Supplementary Tables S4 and S5, which contain all the relevant detail of the modeling we have performed.

Relatively minor critiques:

Introduction

4. First paragraph. The last sentence, where it says "mechanistically investigate" cross-talk, needs more and broader references. The authors only present two references that concern the two pathways in question, which at this point have not even been introduced. At this stage in the intro, they also need more general references about mechanisms of cross-talk, etc.

We have included the following references to the first paragraph of the Introduction. These references further describe which attempts have been made at measuring and investigating signaling crosstalk:
Binder and Heinrich, 2004
Tisch et al., 2014

Dumont et al., 2001
Somsen et al., 2002
Waltermann and Klipp, 2010

5. In the second paragraph, the sentence
"while a small fraction of the active Hog1 remains in the cytoplasm and phosphorylates other" needs a reference.

We have added the following references:
Mollapour et al., 2007
Patterson et al., 2010
Westfall et al., 2008

6. Similarly, in the next paragraph the sentence "A fraction of the active Fus3 then relocates to the nucleus to affect the expression of several genes" needs a reference or rephrasing. Is there hard evidence that a small fraction of Hog1 remains in the cytoplasm or that only a fraction of Fus3 relocates? Or is it more likely that there is constant shuttling and at any given time only a fraction is in the nucleus or the cytoplasm?

While activated Hog1 and Fus3 are known to mostly relocate to the nucleus, a fraction of both have been shown to remains in the cytoplasm, where it phosphorylates cytoplasmic substrates.
We have added the following references to the text, which report observations regarding cytoplasmic location of active Fus3 (Fus3 that is phosphorylated at T180 and Y182) and the role of this MAP kinase in the regulation of cytoplasmic proteins by direct phosphorylation:
Choi et al., 1999
Elion et al., 2001
Parnell et al., 2005

7. In the third paragraph, beginning with "The HOG and the pheromone pathways..." it says: "Ste20 activates Ste11 in both pathways and the response specificity is achieved by means of scaffold proteins (Patterson et al., 2010), kinetic insulation (Behar et al., 2007) and protein relocation (Yamamoto et al., 2010). Further specificity is achieved by mutual inhibition (McClean et al., 2007). As far as I know, there is no proof that scaffolds are the reason for the insulation. Certainly Patterson et al do not show that. As many before, they hypothesize that scaffolds insulate. Similarly, Behar et al do not show that kinetic insulation is at work here. Again, they suggest that such a mechanism might be at work. Finally, the experiments shown by McClean et al that lead to the notion of mutual inhibition, were utterly disproved by Patterson et al 2010.

Indeed, all the mentioned mechanisms are still hypothetical. We have reformulated the text as follows:

"Ste20 activates Ste11 in both pathways. It has been hypothesized that response specificity may be achieved by means of scaffold proteins (Patterson et al., 2010), kinetic insulation (Behar et al., 2007), protein relocation (Yamamoto et al., 2010), and/or mutual inhibition (McClean et al., 2007)."

8. In the same paragraph:

"while another set of enzymes, such as Ssk1, Sst2, Dig1, and Dig2 are known to inhibit the enzymatic activity of different components of the two pathways"
Ssk1, Dig1 and Dig2 have not, as far as I known, an associated enzymatic activity.

We have reformulated the sentence as follows:

"A set of phosphatases are known to de-phosphorylate the two MAPKs in order to inhibit their activity, while another set of proteins, such as Ssk1, Sst2, Dig1, and Dig2 are known to inhibit the activity of different components of the two pathways in other ways."

9. Then, in the same paragraph:
While it is well established that the hyperosmotic stress response inhibits the mating response (Westfall et al.,2008),
I think this is the incorrect reference to a paper from Thorner and colleagues. It should be Patterson et al 2010.

The reference was indeed the wrong one. We have corrected it.

Results

10. First paragraph, it says:
"Accordingly, early time-points after stimulation were favored over later ones, as the activation events of the two MAPK cascades occur predominantly within 10 minutes upon stimulation Supplementary Figure S1"
Where do we see in Fig S1 that only the first 10 minutes are relevant to the pheromone response pathway?

Our focus throughout this work was on pure signaling integration. In particular, we aimed at measuring the transient state of the response induced by the co-stimulation, regardless of the fact that we might not cover the full activation and deactivation of the pheromone MAPK cascade as we measured it for the Hog pathway. By prioritizing the earliest time-points, we therefore wanted to:

a) *have a denser sampling of the* <u>*transient state*</u> *of the response, which would allow us to perform a better reconstruction of the activation dynamic curves*
b) *capture the main* <u>*activation*</u> *events, which take place within the first 20 minutes also for the pheromone pathway*
c) *reduce the influence of* <u>*protein expression*</u> *on the phospho-peptide intensities measured.*

For better clarity, the relative sentence has been reformulated as follows:

"Accordingly, early time-points after stimulation were favored over later ones, as the transient state of the activation of the two MAPK cascades occur within 0-10 (salt) or 10-20 (pheromone) minutes upon stimulation (Supplementary Figure S1)."

11. In the same paragraph:
"Cells were first cell-cycle arrested by Cdc28 analog sensitive inhibition for 1 hour (Shokat and Velleca, 2002) to synchronize their responses to the subsequent stimulations"
Why the authors decided to inhibit Cdc28? Why they did it for one hour? To synchronize which reponse? Both the pheromone and the HOG pathways influence the cell cycle and they in turn are

influenced by it. Please explain in more detail in the text the reasoning and associated references behind this experimental decision.

It has been shown by Strickfaden et al (Cell 2007 Feb 9; 128(3): 519-31) that Cdc28 activity inhibits pheromone response and that, by preventing such inhibition, the pheromone pathway activates even outside G1. The aim of our Cdc28 inhibition was therefore to make cells immediately responsive to pheromone without synchronizing cell cycles.

To better explain our procedure and the reasons behind it, we have inserted the reference above mentioned in the manuscript, and we have reformulated the sentence where we describe why we use Cdc28 inhibition as follows:

"While budding yeast cells are immediately responsive to osmotic shock, a 1 hour long Cdc28 analog sensitive inhibition (Shokat and Velleca, 2002) was employed to make cells immediately responsive to pheromone stimulation (Strickfaden, 2007)."

12. Second page, first paragraph, it says:
"To assess the reproducibility of the measured dynamic profiles and their agreement with published data, we performed NaCl-only and pheromone-only time-course experiments in duplicate." What should we make of this statement? In the legend to Fig1b, it says that the actual co-stimulation experiment was done in triplicates. This control was only done twice? Why? Given that in Fig S1 one can appreciate variation between repetitions, it would be important for the authors to show the behavior of the three biological replicate time courses for at least some of the phosphopeptides in the co-stimulation experiment.

We have performed the NaCl-only and the pheromone-only time-courses by means of 3 biological replicates for each time-point, as we did for each square of the matrix in the co-stimulation experiment. As we have performed these NaCl-only and pheromone-only time-courses twice, each curve refers to a different experiment, not to a different replicate. For better clarity, we have changed the caption to Supplementary Figure S1 as follows:

"Supplementary Figure S1 Cross-experiments reproducibility of the workflow used in this study. We show here the dynamic curves obtained, in two separate but identical time-course experiments, for Hog1_T174_Y176 after NaCl stimulation only (top), and Fus3_T180_Y182 after pheromone stimulation only (bottom). The error bars indicate the variability between the biological triplicates produced for each time-point."

In order to provide more complete information regarding the reproducibility of the data collected for the main co-stimulation Matrix experiment, we have remade Supplementary Table S1 by including, for each phospho-peptide, the following values:

1. *the average intensity (which was already included in the table originally submitted)*
2. *the standard deviation for the intensities of the 3 biological replicates*
3. *the number of replicates that were actually measured (0, 1, 2 or 3)*

In the new table, we have left the values for undetected P-peps blank. These missing values have been subsequently estimated by means of a spline-fitting algorithm, as explained in the manuscript.

In the third paragraph, we have also reformulated the sentence that introduces Supplementary Table S1 as follows:

"The full time-course dataset of all the confidently detected P-peps relative to the HOG and pheromone pathways is available in Supplementary Table S1 where, for each P-pep and for each NaCl- pheromone co-stimulation periods, we indicate the number of detected biological replicates, their average intensity, and their standard deviation."

13. Second page, second paragraph, the authors present an experiment in which they monitor the activating peptide in Hog1 and Fus3, which they show in Fig S1. The stated purpose is to address reproducibility and agreement with published data.
As to reproducibility: What is the error bar presented? Do the authors consider that the two biological replicates are not statistically different? To me, using their definition, I see a "shape effect" between the repetitions for the Hog1 peptide. The statistical methods should be explicit. This comment applies to the rest of the manuscript as well.
As to agreement with published data, the authors do not present a conclusion. Does it resemble or not published data? What would be the papers they are using to compare?
Then, it says:
"The level of phosphorylation [of Fus3] peaked around 20' and then steadily decreased." I cannot see this decrease in Fig S1. On the contrary, I see a steady increase.

We thank the reviewer for this comment as it allowed us to detect a mistake in Supplementary Figure S1. As correctly observed, the shape of Fus3_T180_Y182 dynamic in Figure S1 differs from the one in Figure 2B, even though both graphs represent Fus3_T189_Y182 dynamic. Specifically, in Supplementary Figure S1 the curves continue to increase, while in Figure 2B it reaches a maximum and then starts to decrease. By trying to reproduce the curves from the original dataset, we have realized that the results reported in Supplementary Figure S1 is not relative to Fus3_T180_Y182 but to Fus3_(T180)_(Y182), which is the singly phosphorylated form of the MAP kinase instead of the doubly phosphorylated one. We apologize for this mistake. Furthermore, the dataset used to make Supplementary Figure S1 had not been normalized by Total Ion Current (the normalization strategy used for the main co-stimulation Matrix experiment, as described in the manuscript).

We have normalized the data for Supplementary Figure S1, and the figure has been fixed. In the paragraph "Data validation", we further commented on the behavior of Hog1_T174_Y176 and Fus3_T180_Y182 in the preliminary experiments, highlighting both reproducibility and inter-experiment differences:

"While the curves relative to the two experiments slightly differ in their shape (there is a secondary mild up-regulation of doubly phosphorylated Hog1), the main spikes are very reproducible both in shape and intensity."

"Furthermore, whereas Fus3 activation curves in the two experiments have similar shapes, their overall intensities differ. This difference may be due to a dissimilar starting amount of either Fus3 or of the activating kinases upstream to Fus3 (such as Ste7)."

The error bars in Supplementary Figure S1 represents the standard deviation between biological replicates. For more explanations, please see the answer to question nr. 12.

We have provided the following references that show how the activation of Hog1 and Fus3 takes place, with specific reference to the phospho-peptides Hog1_T174_Y176 and Fus3_T180_Y182:

Muzzey et al., 2009
Schüller et al., 1994
Yu et al., 2008

14. In page three of Results, it says:
"The full time-course dataset of all the detected phosphopeptides relative to the HOG and pheromone pathways is available in Supplementary Table S1."
In table S1, the data corresponds to averages of the three biological replicates mentioned in the legend to Fig1b? If so, the authors should add the standard deviation of the measurements.

The data corresponds to the average of the detected biological replicates. We have added the standard deviation of all the measurements as required, together with the number of effectively detected biological replicates for each co-stimulation times pairs (for each square of the Matrix).

15. Related to the previous comment. In general, in all their figures, the data is presented as a single data point at a given time. Are these averages? Please include the standard deviation of the three biological replicates in the plots.

The data points in the graphs are the averages of the intensities of the detected biological replicates. As the graphs are already quite dense with information, we would prefer not to add the standard deviation, which would make the graphs unreadable. We have however added this information to the Supplementary Table S1, for all of the measured phospho-peptides. Please refer to the answer to question nr. 12 for a better explanation on the new structure of Supplementary Table S1.

16. Next paragraph, it says:
"To investigate changes in phosphorylation, we represented P-pep dynamics profiles as a 3D graph (Figure 3B) and as a combination of 2D charts (Figure 3C).
If x-axis label on Fig3b is correct, then I think that those in 3c (up and down) are swapped. In addition, I suggest change this graph into a 2D plot, maintaining the color code for intensity. In this way, the currently unobservable regions in the 3D will be observable.

The labels in Figure 3b were swapped. We have fixed them.

We would prefer not to change the 3D representation into a 2D plot, as Figure 3c is already a 2D plot and it would be redundant. The 3D representation is just meant to show the complexity of the behavior of a phospho-peptide when two stimuli are being applied together. In order to eviscerate the characteristics of this behavior, it is better to work with the 2D representations given in Figure 3c, which retains all of the information within the 3D plot.

17. The legends to Fig3D and Figure 3D itself are unacceptable. It is not possible to know what peptide is in each lane or what sample is in each column. Also, what similarity metric was used for the clustering? This information is not in the legend or in the methods section.

We have labelled all the rows with the corresponding phospho-peptide name. Also, in order to facilitate the interpretation of Figure 3D, we have inserted the information regarding the stimulation times for both stimuli on the x-axis, on top of the clustering heatmap.

The hierarchical clustering was performed by using the Minkowski distance as the similarity metric. We have added this information both in the caption to the figure and in the Methods section:

“The P-peps belonging to the HOG and the pheromone pathways have been classified by a hierarchical clustering (Fraley and Raftery, 2002), using the Minkowski distance (Karakoc et al., 2006). This clustering analysis was performed by means of the software R (www.r-project.org)...”

18. Regarding the "shape and intensity effects", I could not find in the manuscript (or supplement) an explanation of how the effects were classified, scored, the statistics were made, what p-values they obtained, etc. Or what happened when a peptide exhibited both effects? For the classification, shape was given priority over intensity?

We have added a paragraph to the Materials and Methods section explaining the whole procedure in detail:

“The P-peps belonging to the HOG and the pheromone pathways have been classified by a hierarchical clustering (Fraley and Raftery, 2002), using the Minkowski distance (Karakoc et al., 2006). This clustering analysis was performed by means of the software R (www.r-project.org), while all of the analyses described in the next section as well as any data (2D and 3D) representation were performed by means of MatLab version R2013 (www.mathworks.com).”

We have also added the following paragraph, again to the Materials and Methods section:

“Shape and Intensity Effects

The P-peps NaCl-time-curves and pheromone-time-curves were clustered in two separate sessions. We used K-means clustering, with the Euclidean distance, in order to keep the number of clusters to a minimum, which was 6 for the NaCl-time-curves, and 8 for the pheromone-time-curves. For each P-pep we then observed how many different clusters were assigned to its NaCl- and to its pheromone-time-curves. When these numbers were equal or exceeding 3, then we classified the relative behaviours as Shape effects.

All of the P-peps whose curves belonged to less than 3 clusters were further analyzed as follows. As their curves were very similar, they were averaged: for each NaCl (and pheromone, but separately) time point, the average intensity was computed. Each P-pep was then scored by subtracting the resulting minimum average intensity from the maximum one, and by dividing the result by the average of all the intensities. The behaviour of P-peps that scored above or equal to 0.7 was classified as an Intensity Effect.”

19. Later on, it says:
"Surprisingly, ppHog1 underwent a Shape Effect displayed by a strong and short-lived down-regulation 1' after pheromone stimulation (Figure 5A), before recovering its full intensity within the next 4 minutes (Figure 5B)".
In Figure 5B, this downregulation is labeled "intensity effect". Which is correct?

The mistake is in the text: ppHog1 underwent an Intensity Effect. We have corrected the sentence.

20. Regarding the phosphopeptides found, it would be important to determine which have MAPK consensus sites and which have docking sites for at least Fus3 or Hog1. Have the authors attempted to find this out?

We have observed that 26 out of the 60 (43%) peptides backbone sequences have the "SP" motif, while 12 out of 60 (20%) have the "TP" motif. Both the SP and TP motifs have been indicated as the recognition motifs of the MAPKs, Hog1, Fus3, Kss1, Slt2, as well as of Cdc28 and Pho85.

We have introduced this information in Supplementary Table S1 where, for each phospho-peptide, we have indicated if the SP or the TP motif can be found within its sequence.

We have also introduced the following paragraph to the section "Computation of the observed phosphopeptide dynamics profiles and qualitative exploration of the HOG and pheromone pathways dataset" (page 3 of Results):

"Interestingly, 58% of the naked sequences (that is, without phosphorylations) that we have measured have either the SP or the TP motifs (Supplementary Table S1), which have been identified as the recognition motifs of all the yeast MAP kinases, as well as of a few other proteins (Cdc28, which we have inhibited, and Pho85) (Mok et al, 2010). This indicates that many of the detected P-sites may be direct targets of Hog1 and Fus3."

21. The results in the manuscript suggest a rather poor coverage of phosphopeptides. I say this because many known phosphopeptides were not picked in this analysis. For example, it is well established that Ste11 is activated by phosphorylation, and it is well established that it is activated by both signals. Thus, the fact that Ste11 phopho-Ps do not increase in both conditions suggest to me poor coverage. The authors should comment on this.

The limited number of detected phospho-peptides was partially due to the employment of a shotgun approach and to the limitations of the mass spectrometer's dynamic range. Additional phospho-peptides, other than those reported in the manuscript and in Supplementary Table S1, could still be measured and identified, with our approach, with a false discovery rate (FDR) <1%. These were, however, not considered for the subsequent analyses as they did not match our stringent criteria: they were not detected in at least 4 of the 6 NaCl and pheromone time-points (as stated in the Results section).

We have added the following explanation to the first paragraph of the discussion:

"A fraction of the P-sites of the HOG and pheromone pathways that was identified by previous studies was not detected in the present study. One reason for this could be that the protein digestion employed for sample preparation generated peptides that were either too low or too high in mass-to-charge ratio to be detected by the mass spectrometer. Also, the very nature of a shotgun approach is such that only the most strongly detected peptide ions can be sequenced and therefore annotated. Such shortcoming of this approach was however largely compensated by the measurement of a large number of novel P-sites that we then proved to be involved in the signaling integration."

22. In connection with the results shown in Fig6b, the authors should tell us what are the main results, instead of just showing them in a rather complex Figure.

The aim of Figure 6b is simply to explain with an example how the Specificity Matrices and Vectors are computed. Given the complexity of the resulting Matrices, and the high number of phospho-peptides for which these Matrices were computed, we decided to show the main results by means of the Specificity Vectors in Figure 6C. The Vectors are simpler and more compact, and generally retain the main trends observed in the Matrices (this observation is based on manual curation of the results). The complete Specificity Matrix dataset is available in Supplementary Table S3, while Figure 6c shows the main Specificity Vector results.

We have chosen to describe the results shown in Figure 6C by highlighting the behavior of several phospho-peptides that may help explain the down-regulation of Hog1_T174_Y176 after pheromone stimulation. The latter was the starting point that drove the description reported in the last paragraph of the Results section, which involves several of the major proteins of the two pathways: Ptp2, Gpd1, Ste50, Ste11, Ste11, Ste20 and Pbs2. We believe this description to be sufficient to introduce the reader into the ways the Specificity Measures can be used to investigate the dynamics within the two pathways. Once the method is understood, the reader will be able to look for behaviors of interest that can help him/her formulate novel hypotheses based on his/her own knowledge and specific experience.

For better clarity, we now explain our choices in the Results section as follows:

"As Specificity Vectors retain the main information provided by the Specificity Matrices, we chose to report all the most significant results in Figure 6C as Specificity Vectors, while all Specificity Matrices are reported in Supplementary Table S3."

We also better explained Figure 6b in the relative caption:

"(A) Implementation of the Specificity measure (Schaber et al., 2006) for the quantification of the NaCl- and the pheromone-induced effects over the HOG and pheromone pathways phosphopeptides (P-peps). We named the resulting Specificity measures S_NaCl and S_Phe respectively. For each P-pep, we computed both the Matrix (B.1) and the Vector (B.2) of both S_NaCl and S_Phe. Each value of the Matrix is computed as explained in panel A. Each value of a Vector corresponds to the average of the values of the corresponding Matrix taken column-wise: each value of the Vector S_NaCl, for instance, is the average of all the Matrix values corresponding to a single time after NaCl stimulation. (C) Representation of the most significant Specificity Vectors for the HOG and pheromone pathways. On top we show S_NaCl, on the bottom S_Phe."

Discussion

23. In page three it says:
"Fus3 phosphorylation by Ste20, Ste11, and Ste50 is triggered
Please rephrase, as far as we know, Ste7 phosphorylates Fus3.

We have reformulated the sentence as follows:

"Once the pheromone pathway's machinery has been assembled and recruited to the membrane by pheromone pre-stimulation, NaCl stimulation further activates Ste20, Ste11 and Ste50, thus boosting Fus3 phosphorylation before the NaCl-induced ppFus3 down-regulation is triggered."

24. In the same page, it says:
"Indeed, Patterson et al. 2010) showed that Hog1 inhibition allows for the up-regulation of ppFus3 even during 1 M sorbitol stimulation, supporting our hypothesis"
I could not find where in this reference the authors show Fus3 phosphorylation, let alone an upregulation of it after inhibition of Fus3. Is it in another paper?

The paper is correctly cited. The result we refer to in our manuscript is reported in figure 5, at page 7, where the authors show that Hog1 inhibition, following a strong osmotic shock, induces the activation of the pheromone pathway by crosstalk. They comment in the caption: "Sustained Hog1 catalytic activity is required to prevent cross talk to the mating pathway".

Although they measure the recruitment of Ste5 to the plasma membrane, which is associated with the activation of the mating pathway, they do not measure ppFus3. We therefore reformulated the sentence as follows:

"Indeed, Patterson et al. (2010) showed that Hog1 inhibition, following osmotic shock by 1 M sorbitol stimulation, induces the activation of the pheromone pathway by crosstalk, which supports our hypothesis."

25. In the section entitled: "Gpd1 and Hog1 promote their mutual inhibition"
It says:
"The primary and quickest negative feedback mechanism predicted by Schaber et al. 2012) involves the glycerol production machinery available in cells under normal conditions."
I think Schaber presents this feedback as the most effective, but certainly not the quickest, since its effect requires an increase in glycerol concentration, which is not that instantaneous. Thus, the hypothesis that an increase in glycerol is the reason for the very quick and transient drop in ppHog1 is very unlikely in my opinion.

Schaber et al. (2012) observed in their manuscript that: "the model suggested that the main adaptation mechanism is not via a feedback involving transcription of glycerol-producing enzymes, but rather a fast, possibly posttranslational, Hog1-mediated feedback on the glycerol production machinery". According to their results, the main feedback mechanism does not require glycerol accumulation (which indeed takes time) but simply the "already available glycerol producing machinery", which is mainly Gpd1, as they also mention. This mechanism would therefore reduce Hog1's activation in a measure that is inversely related to the already available Gpd1: if Gpd1 is already highly concentrated in the cytosol, there is no need for Hog1 to be activated and to consequently promote the transcription of more Gpd1.
We have reformulated the sentence as follows:

"The primary and quickest negative feedback mechanism predicted by Schaber et al. (2012) involves the glycerol production machinery that is available in cells before osmotic shock, which they believe to be regulated at posttranslational level. This mechanism would promote a down-regulation of Hog1's activity that is inversely proportional to the amount of the already available glycerol producing machinery. Gpd1, whose transcription is promoted by active Hog1, catalyzes glycerol production in response to osmotic stress and it is inactivated by phosphorylation at S24 and S27 (Oliveira et al., 2012)."

26. In the next paragraph, it says:
"In its doubly-phosphorylated form, Gpd1 is incapable of catalyzing glycerol production, but it might be able to promote the Hog1 down-regulation instead."
Are the authors suggesting that Gpd1 has a role independent of its role in glycerol production? There is no evidence for this function. Why choose this particular protein for such a role? I don't follow the reasoning.

We choose Gpd1 together with a few other proteins (Ptp2, Ste50, Ste11, Ste20, and Pbs2) as they all have a similar S_phe (Specificity due to Pheromone stimulation) pattern (Figure 7). Our special interest in Gpd1 was due also to the fact that Schaber's paper (2012) shows that Gpd1 is strongly involved in the down-regulation of Hog1 activation (please see response to question 25). It is possible that Gpd1 performs other functions other than catalyzing glycerol production, either directly or indirectly. We do not know the mechanism. A follow up investigation focused on Gpd1, and its relation to the TORC2 pathway and to pheromone and

salt stimulation, will be necessary to verify our hypotheses and to assess the mechanism that brings about the described behavior.

27. The authors do not provide the phophopeptide data for the Bit61_S139_S144 peptide. Given the importance it has for the author's argument, they should include it in the dataset. Same for the Ypk1 and Gpd1 peptides.

We have included the mass spectrometry intensity data to the Supplementary Table S1 for both Bit61_S139_S144 and Ypk1_S644_S653. Gpd1 data was already in the table.

28. The authors should discuss the published evidence that Ptc1 is activated by pheromone see Malleshaiah et al, Nature 2010, which might add extra weight to their reasoning.

We have added the suggested reference to the text:

"Ptc1 is a phospho-Ser/Thr-specific phosphatase which is known to bind Pbs2 through the adaptor protein Nbp2 to down-regulate ppHog1, and to be regulated by pheromone stimulation (Malleshaiah et al., 2010)."

Concluding remarks:

29. It says:
"Since one of the first stages of the mating response to pheromone signaling is shmooing, which consists in the formation of a cellular bulge, the cell wall integrity pathway is consequently also activated (Baltanas et al., 2013) and the cytoskeleton needs to be thoroughly reorganized," Buehrer and Errede, 1997 first demonstrated that the CWI pathway is activated by mating pheromone, and that it happens during shmooing. Please add that reference.

We have added the suggested reference.

Methods

30. I think the methods section needs to be expanded quite a bit to include descriptions of all methods used, and statistical treatment of the data post phosphopeptides quantification. That is, all subsequent analysis. As it is now, it only goes to any depth in connection to the protein extraction and mass-spec work.

We have expanded the Methods section to include all the missing information regarding our statistical analyses:

"Search results were evaluated with the Trans Proteomic Pipeline (Keller et al., 2005) using the Peptide Prophet version 4.5.2 (Keller et al., 2002)."

"Probability scores from analysis of peptides by Peptide Prophet were used to filter OpenMS results at a false discovery rate threshold less than 1%."

"The P-peps belonging to the HOG and the pheromone pathways have been classified by a hierarchical clustering (Fraley and Raftery, 2002), using the Minkowski distance (Karakoc et al., 2006). This clustering analysis was performed by means of the software R (www.r-

project.org), while all of the analyses described in the next section as well as any data (2D and 3D) representation were performed by means of MatLab version R2013 (www.mathworks.com)."

"Shape and Intensity Effects

The P-peps NaCl-time-curves and pheromone-time-curves were clustered in two separate sessions. We used K-means clustering, with the Euclidean distance, in order to keep the number of clusters to a minimum, which was 6 for the NaCl-time-curves, and 8 for the pheromone-time-curves. For each P-pep we then observed how many different clusters were assigned to its NaCl- and to its pheromone-time-curves. When these numbers were equal or exceeding 3, then we classified the relative behaviours as Shape effects.

All of the P-peps whose curves belonged to less than 3 clusters were further analyzed as follows. As their curves were very similar, they were averaged: for each NaCl (and pheromone, but separately) time point, the average intensity was computed. Each P-pep was then scored by subtracting the resulting minimum average intensity from the maximum one, and by dividing the result by the average of all the intensities. The behaviour of P-peps that scored above or equal to 0.7 was classified as an Intensity Effect."

We thank Reviewer 3 for the appreciation, the constructive comments and the suggestions on the manuscript. In the following, we present the resulting improvements.

Reviewer #3

In this manuscript, the authors used label-free quantitative proteomics to profile the temporal changes in protein phosphorylation in yeast cell cultures co-stimulated by NaCl and pheromone over time periods ranging from 0 to 45 minutes. They quantified 2536 phosphopeptides (1015 proteins) across 36 conditions (time points/stimuli), and obtained unprecedented details of signal integration within and between these two signaling pathways. They regrouped phosphopeptides based on the shape and intensity of the temporal changes to classify patterns and facilitate the identification of substrates from specific pathways. They also used specificity metrics to quantify the extent of inhibition or amplification of NaCl and pheromone-induced effects on substrates from these two pathways. They showed that phosphopeptides reacted to the stimuli in different ways, and that phosphosites from the same protein can show strikingly different dynamics as described for Ste20, a kinase shared by the two pathways. Interestingly, this study highlighted that the TORC2 pathway modulates Hog1 activity in response to pheromone stimulation, and that phosphorylation of Gdp1 and Hog1 results in their mutual inhibition. Overall, this study is well executed and data are presented in a clear and logical manner. In my opinion, this manuscript should be published in Mol. Syst. Biol., pending minor revisions as described below.

1. On p. 8, last sentence, the authors focus exclusively on phosphopeptides that are derived from proteins associated to the HOG/pheromone pathways. Hence, a relatively small subset of the data was selected for further analysis. Table S1 actually presents data for 53 phosphosites from 20 proteins, a subset representing less than 2 % of the entire dataset. In the context of the present experiment, it would be pertinent to present a more comprehensive view of their results and discuss how many of all phosphosites quantified were found to be regulated in response to the stimuli. It would be interesting to extend the discussion to substrates other than those from the canonical HOG/pheromone pathway to uncover new biological insights.

> *The aim of our study was to investigate the crosstalk between two signaling pathways. By using a shotgun approach, we were able to measure several components of the whole phospho-proteome. Our purpose in using this approach was to attempt the measurement of as many phospho-peptides as possible within the two signaling pathways. Even though we also collected a lot of data relative to several other proteins, we chose to begin our investigation by analyzing the sub-dataset relative two the Hog and the pheromone pathways only. This investigation allowed us to understand which are the advantages and strengths in the approach we used, to assess the data quality, to identify the challenges of data analysis, and to employ modeling tools that are easier to control and bring to better results when applied to smaller datasets. We will certainly extend our analysis on the complete dataset, but we will do so in a different paper.*

2. On p.9 can the authors discuss how they selected dynamic profiles corresponding to regulated phosphopeptides? They indicated that they identified phosphopeptides on 82% of the proteins from both MAPK pathways. However, how many of these were affected by the treatment?

> *The aim of paragraph "Computation of the observed phosphopeptide dynamics profiles and qualitative exploration of the HOG and pheromone pathways dataset" (at p.9) is to provide a description of the dataset relative to the two MAP kinases pathways as well as some simple but meaningful representations of the phospho-peptides dynamics. At that stage, we did not discuss whether the phospho-peptides are regulated or not, as this topic*

was addressed in the next sections of the paper, where we introduce some tools for the investigation of the behavior of regulated peptides.

For our own interest we did however measure how many phospho-peptides are significantly regulated by computing the standard deviation divided by the maximum intensity measured in each time-course. By setting an arbitrary threshold, defined from our visual analysis of the curves representations, we could see that 68% of the phospho-peptides are significantly regulated by NaCl, while 83% are significantly regulated by pheromone.

3. It is somewhat surprising that no GO terms enrichment analysis was performed to confirm cellular pathways regulated by NaCl and pheromone stimulation. This information would be a natural extension of the analysis of regulated sites (point 1 above) and would provide a more global view of the activated pathways such as morphogenesis and cytoskeleton reorganization as discussed in the conclusion section.

We did not perform a Gene Ontology analysis because of two reasons. First, because in this manuscript we have restricted our investigation to the proteins belonging to the Hog and the pheromone pathways (for the reasons outlined above, in response to the Reviewer's question number 1). A GO enrichment analysis would be therefore strongly biased and less meaningful in this particular context. Second, because we did not study the behavior of proteins but of their phospho-peptides and, in many cases, a phospho-peptide is regulated by both stimuli but in different ways, or different phospho-peptides within the same protein are regulated by either NaCl or pheromone. All these information are lost to a GO analysis, which only looks at the protein itself.

While a GO analysis would not provide any additional information in the context of this paper, we have performed it on the complete dataset which, as explained above (question 1), will be the object of another study and another manuscript. From this analysis, we saw that, indeed, cytoskeleton organization is the third most enriched cellular process in the case of proteins that have at least one phospho-peptide that is significantly affected by pheromone, but not by NaCl.

4. On p. 11, the definition of shape and intensity effects is not entirely intuitive and could be clarified further. Also, the authors should provide a clear rationale to evaluate the significance of phosphopeptides undergoing shape and intensity effects.

We have reformulated the paragraph defining Shape and Intensity effects, and their functional meaning, as follows:

"To better understand how the co-stimulation affected the dynamic P-pep patterns, we manually investigated their 2D representations (Figure 3C) along the time-axes for both stimuli. This analysis showed that, in the case of some P-peps, the length of the application of Stimulus_2 (for instance) significantly changed the shape of the curves plotted against the time following the application of Stimulus_1. In the following, we call this the Shape Effect of Stimulus_2 (Figure 4A). Most of the P-pep changes following this pattern occurred in the first 5' following Stimulus_1 application, and they mostly appeared as changes in curve concavity, as an increase/decrease in the number of maximums and minimums of the curves (e.g. a biphasic curve becomes triphasic), or as a change in curve shape with earlier or later onset. All these patterns suggest that Stimulus_2 significantly affected the dynamics of these P-peps by altering their behavior along the Stimulus_1 time-axis.

The dynamic of a second group of P-peps, once plotted against the Stimulus_1 time-axis (for instance), while displaying unvarying curve shapes, exhibited overall significant intensity variability modulated by Stimulus_2 (Figure 4B). Here we call this the Intensity Effect of

Stimulus_2, which does not alter the behavior of the P-peps but it significantly increases or decreases its overall phosphorylation. Each P-pep was thus classified by a Shape Effect, an Intensity Effect, or No Effect after co-stimulation with either NaCl, pheromone, or both (Figure 4C)."

5. On p.15, the discussion on the down-regulation of Ptp2 S258 by pheromone is not entirely clear. For example, a change in phosphopeptide intensity only ~15% (10% with 1' salt) is observed for Ptp2 without salt, is this sufficient to induce the corresponding changes in Hog1 activity? There is no biochemical validation supporting this observation.

We have reformulated the sentence as follows:

"Ptp2 is a nuclear tyrosine-phosphatase known to down-regulate Hog1 (Wurgler-Murphy et al., 1997). However, Ptp2 regulation is unknown. We find that NaCl has little if any influence on Ptp2_S258 phosphorylation, while pheromone down-regulates this site (Figure 4C, Figure 7B). In particular, a 1' long pheromone stimulation down-regulates Ptp_S258, following a pattern that is similar to the one observed for ppHog1. This suggests that Ptp2_S258 is not targeted by Hog1 but rather by a pheromone-dependent signal. It is thus possible that the observed pheromone-induced down-regulation of Ptp2_S258 activates Ptp2, thus leading to Hog1 dephosphorylation. Among the other P-peps that mimicked the ppHog1 Specificity pattern, Ste50_S202 is most strongly affected by the 1' pheromone treatment."

All the predictions reported in this manuscript are based on our observations, and constitute novel hypotheses that may explain the mechanics of the NaCl and the pheromone signals integration. All these hypotheses certainly require specific biochemical validations. We feel, however, that this goes beyond the scope of the present manuscript. As our investigation was aimed at investigating signaling integration, we have focused our analyses on the signals themselves. We hope the predictions we have made will inspire novel research lines in the context of the Hog and the pheromone pathways.

6. On p. 20, the authors discussed the mutual inhibition of Gdp1 and Hog1. The observation that ppGdp1 promotes the down regulation of ppHog1, and that activated Hog1 promotes the dephosphorylation of Gdp1 is interesting. While Ypk1 is known to phosphorylate Gdp1, there is no information available on how Gdp1 inhibits the activity of Hog1 or what phosphatase dephosphorylates Gdp1. Could the authors provide additional information on the potential mechanism accounting for this regulation?

This is an interesting problem. Wherever we predict that protein A down-regulates a certain phosphorylation within protein B, we can only assume it will either do it directly, in the case of phosphatases, or indirectly through the involvement of a phosphatase. This is the case of Gpd1. Gpd1 is not known to have any phosphatase activity and, to our knowledge, it is not yet known to activate or otherwise indirectly affect the activity of any phosphatase. This needs to be addressed in a follow-up experiment focused on the interaction between Hog1, Gpd1 and the TORC2 pathway, which is, in itself, still poorly understood.
For better clarity, we have added the following sentence to the paragraph "Gpd1 and Hog1 promote their mutual inhibition" (pp 20-21) mentioned by the Reviewer:

"The mechanisms through which this is achieved (which phosphatase performs the actual dephosphorylation) needs to be further investigated."

7. On p.20, while Gdp1 catalyzes the production of glycerol in response to NaCl, the glycerol efflux receptor Fps1 is also known to be regulated following osmotic shock. A recent report indicated that upon osmotic shock Hog1 is recruited to a MAPK docking site within the N-terminal domain of Fps1 and phosphorylate a redundant pair of regulators, Rgc1 and Rgc2 to induce their eviction from the C-terminus region and closure of the Fps1 channel (Genes & development 27, 2590-2601, 2013). Can the authors discuss the changes in phosphorylation of Fps1 and Rgc1/2 in the context of their experiments?

Hog1 indeed also controls the activity of Fps1, Rgc1 and Rgc2. In our analysis, we have observed that several phospho-sites within these proteins, some of which also reported in the paper mentioned by Reviewer 3, are phosphorylated immediately upon osmotic shock. Pheromone does not appear to exert a strong influence on most of these phospho-sites, even though we have observed a mild or more pronounced de-phosphorylation of some of them, and an oscillatory behavior in the case of other phospho-peptides. One exception is a singly phosphorylated peptide of Rck1, Rck1_(S966)_(S969)_(S975), which is down-regulated by salt and up-regulated by pheromone.

We did not discuss these results because we thought they would not fit into the current storyline. However, as these proteins are also closely connected with the Hog pathway, we have now included the measured mass spectrometry intensities and the specificity results relative to these proteins to the Supplementary Tables S1 and S3 respectively.

2nd Editorial Decision 10 September 2014

Thank you again for submitting your work to Molecular Systems Biology. We have now heard back from the two referees who were asked to evaluate your manuscript. As you will see below, reviewer #1 thinks that most of their main concerns have been satisfactorily addressed. However, referee #2 lists a number of issues, which we would ask you to address in a revision of the manuscript.

Without repeating all the points listed below, some of the more substantial concerns are the following:
- Importantly, reviewer #2 requests a clearer description of how the mock experiments were performed, to ensure that the results of the mock experiments can be directly compared with those of the pathway-stimulation experiments.
- The reviewer thinks that Western blot analyses are required for validating the main results. While we have no reason to doubt the MS data and we do not feel that the Western blots are essential, we would not be opposed to the inclusion of such data, since they would indeed represent an independent validation of some of the results.
- During the first round of review, both reviewers #1 and #2 were concerned that the functional relevance of (some of) the phosphopeptides is not validated by follow-up experiments. Reviewer #2 is still not convinced that this point has been addressed. From our point of view, we think that while the inclusion of such experiments is not mandatory for publication of the study, it would significantly enhance the conclusiveness of the work.
- Finally, some of the comments of reviewer #2 refer to the need to provide additional information and clarifications regarding the modeling analysis and the related results.

Additionally, we would like to draw your attention to point 9 raised by reviewer#1 during the first round of review. We tend to agree with reviewer #1 that extrapolating the curves (in all figures showing the response time course) does not seem entirely justified. As an example, in Figure 5A, the "20' Phe" curve indicates a value slightly higher than 2.0E-03 as the maximum phosphorylation between 0 and 5 min, but without that this maximum value has been observed experimentally. As such, we would think that simply connecting the measured data points might seem more appropriate.

Thank you for submitting this paper to Molecular Systems Biology.

\---

Reviewer #1:

As I noted previously, the manuscript by Vaga et al presents extensive data on the time course of protein phosphorylation of components of the pheromone and high osmolarity signal transduction pathways in yeast under a variety of combined pheromone and salt treatments. The data are comprehensive, although not exhaustive, encompassing most of the proteins of the two pathways. The data are also of high quality, carefully curated and elicit a number of testable hypotheses.

I and the other reviewers encouraged the investigators to test some of the hypotheses prompted by their data and to assess the biological relevance of some of the phosphorylation sites they identified. In the revised manuscript, the investigators addressed the latter issue by citing additional references that established the significance of some of the phosphorylation sites. However, rather than explore the implications of their observations, the investigators added a section on modeling to the manuscript. This modeling section explored causal relationships among the most robust phosphopeptides and yielded a model that recapitulated the dynamics of the system reasonably well. Moreover, this modeling effort highlighted some known interactions, such as Hog1 and Ptp2, while suggesting some novel interactions.

While the authors have not expanded the manuscript in the direction suggested by the reviewers, the data in the manuscript document a level of crosstalk between the two signaling pathways well beyond that anticipated by previous work. Moreover, the work suggests a number of lines of investigation that should prove quite informative in future experiments. Finally, the data generated will be useful to other investigators in subsequent studies of these pathways.

Minor points:

Figure 3: Fus3_S180_Y182 should be Fus3_T180_Y182

Figure 4: Is there any significance to the topology of the map in (C)? It looks like a phylogenetic tree but if the distances between components are arbitrary perhaps that should be stated in the legend.

Figure 6A: As I understand the terms, S_Phe in the upper right should be S_NaCl and S_NaCl in the lower right should be S_Phe.

Reviewer #2:

In this revised version the authors responded to most of my concerns satisfactorily. There are some outstanding issues that I feel are important to solve.

Main concern:
The authors have decided not to verify by Western blot (or another technique) one of the main results in the paper: the drop in the abundance of the doubly phosphorylated form of the activation loop peptide of Hog1 in the 1' pheromone exposure samples. They argue that the antibodies do not distinguish the doubly phosphorylated form from the singly phosphorylated version. However, there is commonly used antibody claimed to recognize only the doubly phosphorylated form of p38 (CellSignal #9216). If still concerned, the authors may include a simple control, such as treating the protein extract with tyrosine or Ser/Thr phosphatases to remove only one of the phosphates at a time. Alternatively, they could run a sample obtained from strains with mutations in the activation loop.
Running a Western blot is a rather simple experiment (especially in comparison with the sophisticated mass-spec experiments performed by the group) that will strengthen the results significantly. It involves only a few time points, comparing "real" with "mock" stimulation for the 1' pheromone case.
Other concerns:
1) The authors added a whole new section with a modeling effort, in order to investigate to some extent the potential roles of the dynamics of the phosphopeptides they measured. I like the idea, the approach and the findings. However,
a. Could the authors include the actual ODEs used in the simulations? Otherwise, it is not possible to evaluate the model.
b. It is not clear if the best model (Fig8) performs really well, as the authors state. I see that among the peptides that have a worst match between data and model are those belonging to the activating loops of Fus3 and Hog1. Or am I reading Fig8b incorrectly?
c. The authors simulated only three experiments, each stimulus alone, and the simultaneous addition of NaCl and pheromone. I think it would be important to show that the model also captures the more interesting (and potentially more challenging) time combinations, such as the 1' pheromone case.
d. In the Results section dealing with the modeling results. In the paragraph beginning with "Our results show that the model..." I don't understand how the authors draw this conclusion:
"This suggests that this feedback loop might be enhancing the signaling integration at the data points which were excluded the models training" given this result:
"The further addition of the ppGpd1-ppHog1 reciprocal inhibition mechanism also showed no significant increase of fitness (MSE=0.059) and, accordingly, a slight decrease in AIC (AIC=- 937) due to the extra complexity".
To me it only suggests that the reciprocal inhibition is not involved in the stimulation regime simulated.
e. Could the authors expand on the explanation of this conclusion: "We therefore suggest that, according to our data, the interaction between Hog1 and Ptp2 is not mediated by the phosphorylation of Ptp2 at Ser258." ?

2) Regarding the mock experiment. It is not clear to me from reading the text if the mock experiment was done "alone" or in parallel with a new "real" stimulation. If it was done "alone", then it doesn't fully serve the intended purpose, since it is conceivable that in this particular case, whatever technically derived stress the yeast suffered in the original experiment wasn't present

during the mock experiment.

3) Regarding their response to my point 11.
To be fair, it has been shown way before that Strickfaden 2007 that Cdc28 activity inhibits pheromone response, and that it does so using Cln1 or Cln2. See Oehlen and Cross 1994. Strickfaden et al showed us that the target of such inhibition is Ste5.
Also to be fair, the first use of Cdc28 analog sensitive mutants to "chemically synchronize" cells while studying pheromone response seems to be in Colman-Lerner et al 2005.
These two references should be added.

Regarding their response to my point 13.
The references they present for the Hog1 phosphorylation dynamics seem incorrect to me:
In Muzzey et al, there appears to be no western blots or any other measure of Hog1 phosphorylation.
In Shuller et al, they don't have Hog1 phospho-specific antibodies.

So, these references are not useful to support the claim that the pattern observed by Mass-Spec matches previous published data.
As a side point, if there were such references, it would mean that the authors do trust to some extent at least previous measures of Hog1 activation done by western blots to compare with their mass-spec. This is in relation to the requested Western blot to cross validate the mass-spec for the drop on Hog1-pp after 1' pheromone stimulation.
Please add appropriate references.

4) In the Results section. The labeling in Fig3D seems to have a mistake for Fus3. It says in one of the peptides S180, but it is T180.
5) Supplementary table 4 does not have the "full list of peptides" I only see a short list of 8 peptides.

Comments on the rebuttal to Reviewer 1:

Regarding R1 point 1:
I think that to address the reviewer's concern, the authors could show that the different dynamics obtained via the combination of both stimuli actually lead to different behavior, in the longer term. It is not clear that a drop in Hog1 phosphorylation in the first minute post pheromone addition affects in a measurable way the ability of the HOG pathway to adapt after a shock, for example. So, maybe an experiment measuring and comparing recovery times (for example) in the various stimulation regimes would be useful.
Thus, I do not think the authors have responded satisfactorily to this critique.

Regarding R1 point 2:
I had a similar concern. I do not feel the authors have responded to my satisfaction. However, I do agree with the authors a large value of the paper comes for the "unprecedented dataset" that it generated and that it will be for the future to test the relevance of the dynamics found. Having said that, I don't see why they refuse to test if at least one new phosphopeptides is important, for example by making a non-phosphorylatable mutant and measure an altered response (volume recovery time for HOG for example). The paper makes a good case for the Ste20-T511 being important.

As far as I can see, the authors have responded well to Reviewer 3.

2nd Revision - authors' response 10 October 2014

(see next page)

© European Molecular Biology Organization

We thank the Editor and the Reviewers for their comments on our manuscript. In the following point-by-point responses, we have addressed their respective comments using italic font.

Reviewer #1

As I noted previously, the manuscript by Vaga et al presents extensive data on the time course of protein phosphorylation of components of the pheromone and high osmolarity signal transduction pathways in yeast under a variety of combined pheromone and salt treatments. The data are comprehensive, although not exhaustive, encompassing most of the proteins of the two pathways. The data are also of high quality, carefully curated and elicit a number of testable hypotheses.

I and the other reviewers encouraged the investigators to test some of the hypotheses prompted by their data and to assess the biological relevance of some of the phosphorylation sites they identified. In the revised manuscript, the investigators addressed the latter issue by citing additional references that established the significance of some of the phosphorylation sites. However, rather than explore the implications of their observations, the investigators added a section on modelling to the manuscript. This modelling section explored causal relationships among the most robust phosphopeptides and yielded a model that recapitulated the dynamics of the system reasonably well. Moreover, this modelling effort highlighted some known interactions, such as Hog1 and Ptp2, while suggesting some novel interactions.

While the authors have not expanded the manuscript in the direction suggested by the reviewers, the data in the manuscript document a level of crosstalk between the two signaling pathways well beyond that anticipated by previous work. Moreover, the work suggests a number of lines of investigation that should prove quite informative in future experiments. Finally, the data generated will be useful to other investigators in subsequent studies of these pathways.

We thank Reviewer 1 for the appreciation and critical analysis of our manuscript. In the following, we address his remaining concerns.

Minor points:

1. Figure 3: Fus3_S180_Y182 should be Fus3_T180_Y182

We have performed the correction to Figure 3.

2. Figure 4: Is there any significance to the topology of the map in (C)? It looks like a phylogenetic tree but if the distances between components are arbitrary perhaps that should be stated in the legend.

The topology reported in Figure 4C is entirely derived from previous knowledge, as at this stage of our analysis we were not attempting to infer any new functional connections. For better clarity, we have changed the caption to panel C of Figure 4 as follows:

(C) The most significant results of the Shape and Intensity Effects classification are here reported, for the HOG and pheromone pathways P-peps, using a protein topology derived from previous knowledge (Figure 1A). For each P-pep we display two color-coded squares: the top one shows the effect of NaCl on the P-pep pheromone dynamics, and the bottom one shows the effect of pheromone on the P-pep NaCl dynamics.

3. Figure 6A: As I understand the terms, S_Phe in the upper right should be S_NaCl and S_NaCl in the lower right should be S_Phe.

We have performed the correction to Figure 6A.

Reviewer #2

Review of revised version of MSB#56635 by Aebersold and collaborators. In this revised version the authors responded to most of my concerns satisfactorily. There are some outstanding issues that I feel are important to solve.

Main concern:

The authors have decided not to verify by Western blot (or another technique) one of the main results in the paper: the drop in the abundance of the doubly phosphorylated form of the activation loop peptide of Hog1 in the 1' pheromone exposure samples. They argue that the antibodies do not distinguish the doubly phosphorylated form from the singly phosphorylated version. However, there is commonly used antibody claimed to recognize only the doubly phosphorylated form of p38 (CellSignal #9216). If still concerned, the authors may include a simple control, such as treating the protein extract with tyrosine or Ser/Thr phosphatases to remove only one of the phosphates at a time.
Alternatively, they could run a sample obtained from strains with mutations in the activation loop. Running a Western blot is a rather simple experiment (especially in comparison with the sophisticated mass-spec experiments performed by the group) that will strengthen the results significantly. It involves only a few time points, comparing "real" with "mock" stimulation for the 1' pheromone case.

We have been reluctant to employ western blot as a validation technique for two reasons. First because, to our repeated past experience, the antibody that supposedly recognizes the doubly phosphorylated form of Hog1 (the one used in CellSignal #9216) appeared to be less efficient at doing so than mass spectrometry has been. We have anyway tried to repeat the western blot validation (already before the 1st submission), but we were not able to produce conclusive results. Second, because the quantitative resolution provided by western blot is not good enough to resolve the differences observed by mass spectrometry. As a side note, we would like to observe that, among all the phospho-peptides that we have measured across several experiments, for this and for other projects as well, Hog1_T174_Y176's signal has always been remarkably clean and reproducible, constantly detected by any mass spectrometer employed (unlike many other peptides), with a clear and reproducible pike maximum intensity - with minor differences caused by the different experimental conditions. We therefore particularly trust our measures of Hog1_T174_Y176's behavior.

Other concerns:

1. The authors added a whole new section with a modelling effort, in order to investigate to some extent the potential roles of the dynamics of the phosphopeptides they measured. I like the idea, the approach and the findings. However:

1.a Could the authors include the actual ODEs used in the simulations? Otherwise, it is not possible to evaluate the model.

The Reviewer refers to the crucial issue to ease the understanding of the modelling approach. To address it, we have added the following to Materials and Methods section:

"Finally, proteins in the logic model corresponding to Figure 1A were replaced by the P-peps that passed the filtering process, and thereby a model of the state-of-the-art role of the measured P-peps within the HOG and the pheromone pathways was assembled.

Next, we implemented a system of equations where each equation represents the level of one signaling intermediate in the model. To that end, the logic-ODE approach (Wittmann et al., 2009), allows us to express the change over time in the normalized abundance of each P-pep as a function of its regulatory P-peps, i.e. its inputs. Consider for example that Hot1 is phosphorylated at S153 by Hog1 doubly phosphorylated at T174 and Y176. The change over time in abundance of Hot1_S153 can be therefore represented as:

$$H\dot{o}t1\_S153 = \left[\left(1 - \frac{Hog1\_T174\_Y176^n \big/ \left(k^n + Hog1\_T174\_Y176^n \right)}{1 \big/ \left(k^n + 1 \right)} \right) - Hot1\_S153 \right] \times \tau_{Hot1\_S153}$$

where the level of Hot1_S153 depends on the abundance of Hog1_T174_Y176, and on a degradation rate that assumes that dephosphorylation is proportional to the abundance of Hot1_S153. The parameter τ is a time-scale of the activation of Hot1_S153, and both n and k are the parameters of a Hill function for normalization."

Furthermore, the main models, processed and filtered data, estimated parameters and a documented script are available online at the link provided in the Results (http://www.cellnopt.org/data/yeast/).

1.b It is not clear if the best model (Fig8) performs really well, as the authors state. I see that among the peptides that have a worst match between data and model are those belonging to the activating loops of Fus3 and Hog1. Or am I reading Fig8b incorrectly?

The Reviewer is correct. While the model seems to capture the behavior of most phospho-peptides, some are badly represented. It is therefore not adequate to state that the model performs well. We exclusively used the model to compare topological variants in the light of the data. Hence, we rephrased our conclusion, removed the statement claiming that the model correctly captures the trend in the data, and emphasized that the purpose of the modelling effort was to discriminate between model variants representing different mechanisms:

"We used our models to compare the likelihood of the proposed novel mechanisms based on the experimental data. Our results show that the model, extended with our proposed mechanisms (Figure 8B), performs better than the prior knowledge-based one shown in Supplementary Figure S7."

1.c The authors simulated only three experiments, each stimulus alone, and the simultaneous addition of NaCl and pheromone. I think it would be important to show that the model also captures the more interesting (and potentially more challenging) time combinations, such as the 1' pheromone case.

We fully agree that it would be highly interesting to further extend this modelling approach in order to understand how the architecture involved in signaling changes, at the phospho-peptide level, to adapt the cellular response to varying stimulation durations. Unfortunately, it was not possible to extend the method presented here or develop a new approach in the time given for this response. We therefore now mention in the Discussion that we believe it would be crucial to pursue this:

"In this study, we included a modeling effort to assess how the mechanisms here presented could integrate the responses to NaCl and pheromone stimulation. While this proved to be informative when comparing models representing different variants of those mechanisms, we anticipate that further insight will be gained by extending the optimization procedure to include the data measured upon varying combinations of length in NaCl and pheromone stimulation."

1.d In the Results section dealing with the modelling results. In the paragraph beginning with "Our results show that the model..." I don't understand how the authors draw this conclusion: "This suggests that this feedback loop might be enhancing the signaling integration at the data points which were excluded the models training" given this result: "The further addition of the ppGpd1-ppHog1 reciprocal inhibition mechanism also showed no significant increase of fitness (MSE=0.059) and, accordingly, a slight decrease in AIC (AIC=- 937) due to the extra complexity". To me it only suggests that the reciprocal inhibition is not involved in the stimulation regime simulated.

The conclusion indeed appears wrong because of the unclear explanation of the results it was drawn from. We have modified it as follows:

"The further addition of the ppGpd1-ppHog1 reciprocal inhibition mechanism also showed no significant increase of fitness (MSE=0.059) and, accordingly, a slight decrease in AIC (AIC=-937) due to the extra complexity. Since the analysis of the specificity matrices (Figure 7F) indicates that ppGpd1 and ppHog1 are involved in each other's down-regulation, these two observations suggest that this feedback loop might be enhancing signal integration at the data points excluded from model training (i.e. co-stimulation by both NaCl and pheromone, but not simultaneous).

1.e Could the authors expand on the explanation of this conclusion: "We therefore suggest that,

according to our data, the interaction between Hog1 and Ptp2 is not mediated by the phosphorylation of Ptp2 at Ser258." ?

The Reviewer refers to a sentence that was indeed confusing because of a typo. We have corrected as follows:

"This indicates that, as previously known, the regulation of Hog1's phosphorylation by the phosphatase Ptp2 is essential also during NaCl stimulation. We therefore suggest that, according to our data, the interaction between Hog1 and Ptp2 is mediated by the phosphorylation of Ptp2 at Ser258."

2. Regarding the mock experiment. It is not clear to me from reading the text if the mock experiment was done "alone" or in parallel with a new "real" stimulation. If it was done "alone", then it doesn't fully serve the intended purpose, since it is conceivable that in this particular case, whatever technically derived stress the yeast suffered in the original experiment wasn't present during the mock experiment.

We did not repeat the "real" stimulation experiments for two reasons. First, because all the matrix experiments were performed across several months, as it was technically not possible to do otherwise. Given this fact, we reasoned that it was unnecessary to repeat the "real" stimulation time-course, as we would have had to anyway perform the two experiments in two distinct days. Second, and most important, because we have been able to consistently obtain comparable results when repeating the same time-course experiment after months or even after years. In Supplementary Figure S1, for instance, we have reported some representative results from two time-course experiments performed with a six months' time-difference. Had we observed a significant difference between the results of the mock_pheromone and of the 0'_pheromone, we would have investigated causes of such difference, but that was not the case (Supplementary Figure S3). It was indeed interesting to observe that, in Supplementary Figure S3, the 0'_pheromone and the mock_pheromone gave comparable results both in shape and intensity, although the two experiments were performed with a 2 years difference. Please note that the intensities in Supplementary Figure S1 are considerably different from all the others, as these preliminary experiments were performed on a different mass spectrometer (LTQ-FT instead of LTQ-Orbitrap XL).

3. Regarding their response to my point 11. To be fair, it has been shown way before that Strickfaden 2007 that Cdc28 activity inhibits pheromone response, and that it does so using Cln1 or Cln2. See Oehlen and Cross 1994. Strickfaden et al showed us that the target of such inhibition is Ste5.
Also to be fair, the first use of Cdc28 analog sensitive mutants to "chemically synchronize" cells while studying pheromone response seems to be in Colman-Lerner et al 2005. These two references should be added.

We have added the suggested references.

4. Regarding their response to my point 13. The references they present for the Hog1 phosphorylation dynamics seem incorrect to me:

In Muzzey et al, there appears to be no western blots or any other measure of Hog1 phosphorylation.
In Shuller et al, they don't have Hog1 phospho-specific antibodies.

So, these references are not useful to support the claim that the pattern observed by Mass-Spec matches previous published data.

As a side point, if there were such references, it would mean that the authors do trust to some extent at least previous measures of Hog1 activation done by western blots to compare with their mass-spec. This is in relation to the requested Western blot to cross validate the mass-spec for the drop on Hog1-pp after 1' pheromone stimulation. Please add appropriate references.

Muzzey et al. showed the dynamic of Hog1 enrichment after a 0.4M NaCl stimulation, which had been previously demonstrated to be the result of Hog1 double phosphorylation at T174 and Y176. We specifically referred to Figures 2 and 3 in Muzzey et al. (2009), where they show the dynamics of Hog1_T174_Y176 enrichment within the nucleus, which is indeed comparable to the dynamics of Hog1 phosphorylation as we have measured it in our study. We have however removed Shuller et al. from our references since, as the Reviewer correctly observed, they studied the functionality of Hog1 after T174 and Y176 mutation without any dynamic investigation.

5. In the Results section. The labeling in Fig3D seems to have a mistake for Fus3. It says in one of the peptides S180, but it is T180.

We have corrected the mistake in Figure 3D.

6. Supplementary table 4 does not have the "full list of peptides" I only see a short list of 8 peptides.

We have updated Supplementary Table S4 with additional and more detailed information (the phospho-peptides discarded and the filtering criterion on which this rejection was based), and we have included a new Supplementary Table S5 (see Supplementary material), which lists all the 33 measured phospho-peptides that were included in the model.

Comments on the rebuttal to Reviewer 1:

Regarding R1 point 1:

I think that to address the reviewer's concern, the authors could show that the different dynamics obtained via the combination of both stimuli actually lead to different behavior, in the longer term. It is not clear that a drop in Hog1 phosphorylation in the first minute post pheromone addition affects in a measurable way the ability of the HOG pathway to adapt after a shock, for example. So, maybe an experiment measuring and comparing recovery times (for example) in the various stimulation regimes would be useful. Thus, I do not think the authors have responded satisfactorily to this critique.

We agree with the Reviewers. Further experiments aimed at identifying the effect of different types of co-stimulation on the cellular response would indeed provide a more complete understanding of the input/output relation within the signaling network, whereas in this work we have focused our efforts in investigating the signal processing occurring between input and output. We have therefore attempted to make new experiments aimed at assessing the cells response to NaCl-pheromone co-stimulation. Unfortunately, we did not manage to obtain satisfactory and complete results within the 1 month time allowed for the response. We will therefore simply report here our attempts, and the results we have got.

It is known that Hog1, once activated by phosphorylation at T174 and Y176, relocates to the nucleus, Hog1 relocation has thus been used in other studies as readout for Hog1 activity and for osmotic shock response. Relocation and phosphorylation should follow almost the same dynamics but, when the time-scale is in the range of very few minutes, a microscopy assay may give imprecise information. For instance, to our experience, Hog1 relocation dynamic is slightly different in a microfluidic chip than in the well-slide where you pipette NaCl to the medium. In this assay, we have used a strain harboring Hta2-CFP as a nuclear marker, pRPS2-mCherry as a cytoplasmic marker, while Hog1 was endogenously tagged with YFP. Using cytoplasmic and nuclear markers we could segment and track the cells, and look at the amount of Hog1 in the nucleus. We quantified the ratio between Hog1 in the nucleus and in the cytosol (the ratio is reported in the figure below on the y-axis).

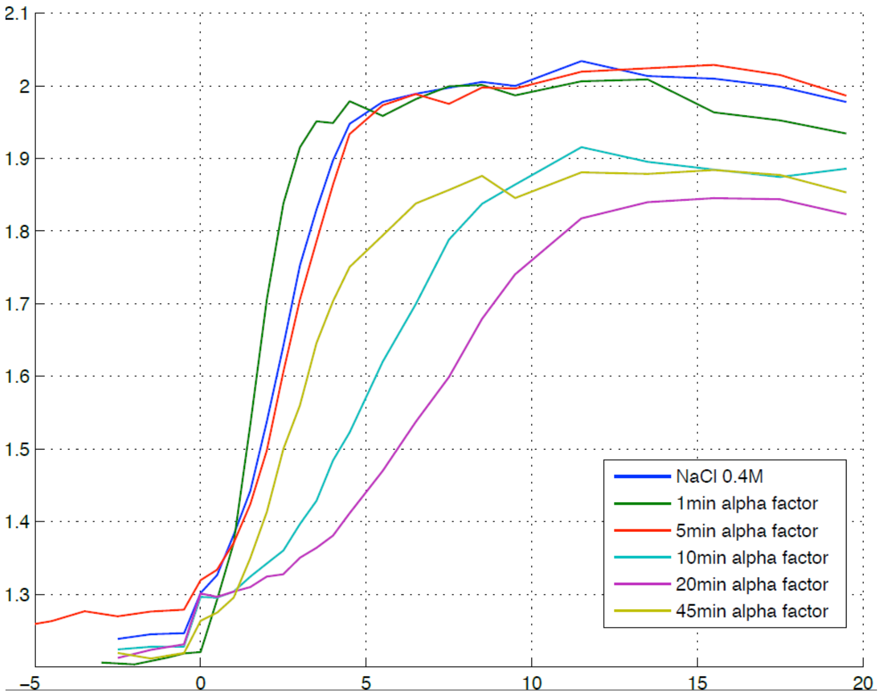

Our results show that pheromone stimulation has an effect on Hog1 relocation to the nucleus. Hog1 relocation is delayed and reduced in amount, and such an effect is more pronounced for longer pheromone pre-stimulations, although the 45' pre-stimulation showed a partial recovery. Unfortunately, from these results we cannot draw any significant conclusion, except observe that pheromone is exerting a clear effect on Hog1 nuclear relocation. We do not believe this to be due to the short and strong down-regulation of Hog1_T174_Y176, as this particular phenomenon is immediately recovered, as we have shown. It may however be related to the combined action of other negative feedback regulation, some of which we have observed, while other were predicted and described by Baltanas et al., 2013. We wish to look into this matter more closely, but we cannot perform any more experiments within the time frame of the present work.

Regarding R1 point 2:

I had a similar concern. I do not feel the authors have responded to my satisfaction. However, I do agree with the authors a large value of the paper comes for the "unprecedented dataset" that it generated and that it will be for the future to test the relevance of the dynamics found. Having said that, I don't see why they refuse to test if at least one new phosphopeptides is important, for example by making a non-phosphorylatable mutant and measure an altered response (volume recovery time for HOG for example). The paper makes a good case for the Ste20-T511 being important.

We have chosen not to perform any validation assay in the current work as that was, indeed, not the purpose of our study. Also, given the potentially large amount of time required to perform satisfactory validations, we wish this interesting investigation to be fully addressed in a separate study of its own.

As far as I can see, the authors have responded well to Reviewer 3.

3rd Pre-acceptance letter 17 October 2014

Thank you again for submitting your revised work to Molecular Systems Biology. We are now satisfied with the modifications made and we think that, pending some minor editorial issues, the study is suitable for publication.

© European Molecular Biology Organization
